# Nucleotide Substitution Biases in Related Cancer Driver Genes

**DOI:** 10.3390/ijms262411903

**Published:** 2025-12-10

**Authors:** Adam Khadre, Yifan Dou, Golrokh C. Mirzaei, Ruben C. Petreaca

**Affiliations:** 1 Department of Computer Science and Engineering, The Ohio State University, Columbus, OH 43210, USA; khadre.3@buckeyemail.osu.edu (A.K.); dou.81@buckeyemail.osu.edu (Y.D.); 2 Department of Molecular Genetics, The Ohio State University, Marion, OH 43302, USA; 3 The Ohio State University James Comprehensive Cancer Center, Columbus, OH 43210, USA

**Keywords:** mutation bias, nucleotide substitution, purifying selection, cancer

## Abstract

Nucleotide substitutions are common in cancer cells, and they occur in both protein coding regions and non-coding regions (5′ and 3′ UTRs and introns). Although substitutions in non-coding regions have the potential to alter gene expression, it is the alteration of coding regions that affects protein function and has the most drastic effect on cellular transformation. Mutations in certain genes (e.g., *TP53*, *KRAS*) are common to nearly all cancers, but most cancers are characterized by specific gene mutation signatures. In this report, we investigated nucleotide substitution signatures in coding regions of the top 25 most frequently mutated genes in multiple human cancers. The goal was to examine whether unique nucleotide substitution biases are associated with various cancers. A pan-cancer analysis showed that the most altered nucleotide is guanine, which is biased towards G->A transitions. A per-cancer analysis identified ten cancers with biased substitutions in certain genes. Some of these biases were expected (e.g., *KRAS* in gastrointestinal cancers or *JAK2* in blood cancers). Our analysis revealed biased signature substitutions in 17 genes, of which 14 were characterized as drivers and constituted a closely related set of cell cycle regulators. We conclude that nucleotide substitution biases contribute to specific alterations in cancer genes that produce cellular transformation. Principle component analysis of nucleotide substitutions shows that most cancers cluster together, meaning that they have similar nucleotide changes. However, certain cancers, most notably lung, pancreas, and blood cancers, can be differentiated from each other based on specific nucleotide signatures. Thus, nucleotide substitution patterns can be used to differentiate between some cancers.

## 1. Introduction

Mutation is the primary driver of genetic alteration that produces both evolutionary change and cellular transformation which causes cancer [1,2]. Mutations can be generated by endogenous processes such as DNA replication or may be caused by exogeneous factors like mutagens and carcinogens. Recent comprehensive analyses of cancer genomes have shown that endogenous and exogeneous factors can cause distinct patterns of mutation signatures [3,4,5,6,7,8]. Although many forms of genomic alterations are observed in cancer cells (e.g., nucleotide substitutions, deletions, insertions, translocations, and other chromosomal re-arrangements), substitutions are the most common [9].

Not all nucleotide substitutions have a similar effect on cellular transformation. For example, synonymous mutations do not alter the protein sequence and are considered less deleterious than non-synonymous mutations. However, new evidence has shown that even synonymous mutations can act as driver of cellular transformation as they may affect transcription, translation, and mRNA stability among other factors [10,11,12,13,14,15]. Non-synonymous mutations have a greater effect and can significantly alter or even inactivate gene function. However, of the 20,000+ genes in the human genome, mutations in only a few have been shown to significantly contribute to cancer. Those genes that have the greatest potential to affect cellular transformation and immortalization have been called “driver” genes [16], while genes that do not significantly contribute to cancer are known as “passenger” genes [17].

Mutation hotspots have been identified in certain genes. A hotspot is defined as a residue or a region of a gene that is mutated more often than would be expected (e.g., found in multiple cancer patients) [18]. For example, the *KRAS* proto-oncogene accumulates primarily the G12D/V/C mutation [19], and *IDH1* has a high-frequency signature at R132H [20]. Numerous other examples exist [21]. The accumulation of these hotspots in cancer genomes is driven by so-called “purifying selection”, which is defined as removal of harmful mutations and selection of mutations that promote fitness [22,23,24,25]. Purifying selection acts both at the evolutionary level and in cancer [18,26].

Although mutations in certain key cell cycle regulators permeate nearly every cancer (e.g., *TP53* [27]), most cancers are characterized by mutations in a distinct set of genes that promote transformation and metastasis [28,29,30]. This important cancer classification has allowed development of targeted therapies that are cancer-specific [31,32] as opposed to broad spectrum chemotherapy [33]. Unlike chemotherapy, targeted therapy has the advantage of reducing side effects, though it often leads to acquired resistance [34,35]. Targeted therapy also requires a deeper molecular understanding of specific mutation that can be actionable (e.g., single molecule inhibitors can be developed) [36,37]. Thus, an understanding of the nature of somatic mutations acquired by cancer cells is important for the development of increasingly effective therapies.

With technological advances in high-throughput genome sequencing, there has been a push to identify cancer mutation signatures. Genome data have been deposited in repositories such as the Catalogue of Somatic Mutations in Cancers (COSMIC) [38], and investigators have used these data to identify cancer signature profiles. For example, an elegant study by Alexandrov et al. [9] has identified and catalogued several single-nucleotide mutation signatures associated with various cancer etiologies. Several other studies using the same COSMIC data have revealed more complex signatures such as DNA double-strand breaks patterns and have also illuminated some of the molecular processes by which they occur [21,39,40]. Other repositories, such as the cBioPortal [41,42] or The Cancer Genome Atlas [43], have also been used to identify cancer signatures [7]. These studies have laid the groundwork distinguishing cancers based on mutational profiles as well as the development of clinical therapies [8,44].

In this report, we built on these previous studies and used the COSMIC database [38] to identify signatures in the 25 most frequently mutated genes in every human cancer. Our reasoning was that while previous studies characterized genome-wide mutations, most cancers are characterized by alterations in a few key cell cycle regulators. The goal was to see if certain nucleotide substitutions are more likely to occur than others within the most frequently mutated genes rather than looking at entire genomes.

## 2. Results and Discussion

### 2.1. Pan-Cancer Nucleotide Substitution Patterns in 25 Most Frequently Mutated Genes

**Figure 1:**
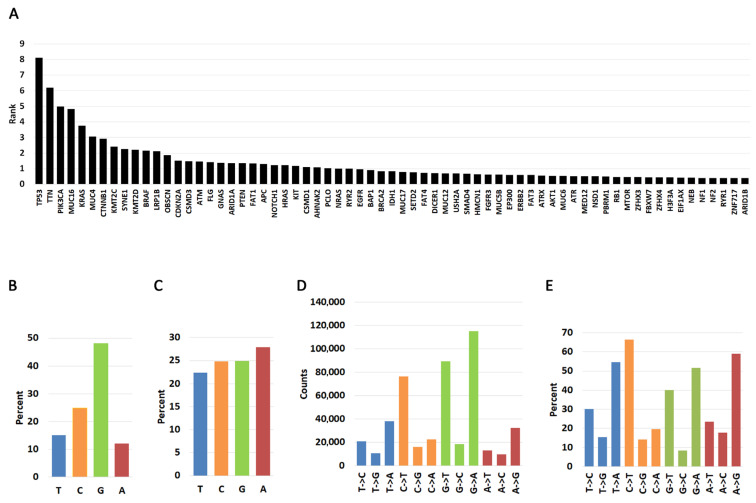
Pan-cancer distribution of nucleotide changes in the 25 most frequently mutated genes in human cancers. (**A**). Pan-cancer analysis of most frequently mutated genes. The graph shows the cumulative rank of most mutated genes. Please see Material and Methods for description. (**B**). Pan-cancer percent occurrence of mutations by T, C, G, and A nucleotide types. The graph shows how likely it is for a mutation to occur in each of the four nucleotides in the 25 most frequently mutated genes. (**C**). Pan-cancer percent nucleotide occurrence in the 25 most frequently mutated genes. (**D**). Frequency of nucleotide change occurring for each of the four nucleotides. (**E**). Percent of nucleotide change type occurring for each of the four nucleotides. Transitions and transversions from each nucleotide are represented as a fraction of 100% (e.g., the three T->C, T->G, T->A changes should add up to 100%).

We first performed a pan-cancer analysis of nucleotide substitutions. Using the COSMIC database, we downloaded nucleotide substitutions data for 43 cancers and an additional file where cancers were not specified (NS). For the analysis presented in this report, we only interrogated mutation signatures in the 25 most frequently mutated genes, which represents 7,035,115 total mutations. Note that this includes multiple samples with the same mutation (e.g., hotspots). This pan-cancer parsing of the most frequently mutated genes in each cancer revealed an expected trend: *TP53* was the most mutated gene followed by several other cell cycle regulators (Figure 1A). Although for *TTN* and *MUC16* there is evidence that relevant mutations have been detected in cancer cells [45,46], the data needs to be taken with a caveat because it can lead to false positives due to the length of the genes (104,301 and 46,191 nucleotides, respectively) [47]. Longer genes are more likely to register mutations that may not be as significant to cancer development. For example, *KRAS* has only 5417 nucleotides, and its cancer frequency of mutations and their driver potential are well established, while the role of *TTN* and *MUC16* mutations in driving cancers is debatable. Integrative Oncogenomics lists *KRAS* as a driver gene but not *MUC16* and *TTN* [16], clearly indicating that gene function matters more than length when registering mutation frequency and cellular transformation potential.

We next investigated a pan-cancer nucleotide substitution bias within the 25 most frequently mutated genes (Figure 1B, Supplementary Table S1B). By far most substitutions involve guanine (48.13%), followed by cytosine (24.18%), thymine (15.09%), and adenine (11.95%). The high frequency of guanine substitutions is not due to a higher percentage of guanines because the occurrence of the four bases is roughly equal in the coding regions of the genes studied here (Figure 1C). Thus, we interpret these findings to mean that substitutions from guanine are more likely than those from any other base. When we investigated the nature of the mutation, we discovered that mutations from thymine are biased primarily towards transversions to A (T->A) and secondarily to C (T->C) (Figure 1D,E, Supplementary Table S1B). However, cytidine and adenine mutations are biased towards transitions (C->T and A->G) while guanine mutations can occur with equal probability towards transversions (G->T, G->C) and transitions (G->A).

### 2.2. Cancer-Specific Biased Substitutions

**Figure 2:**
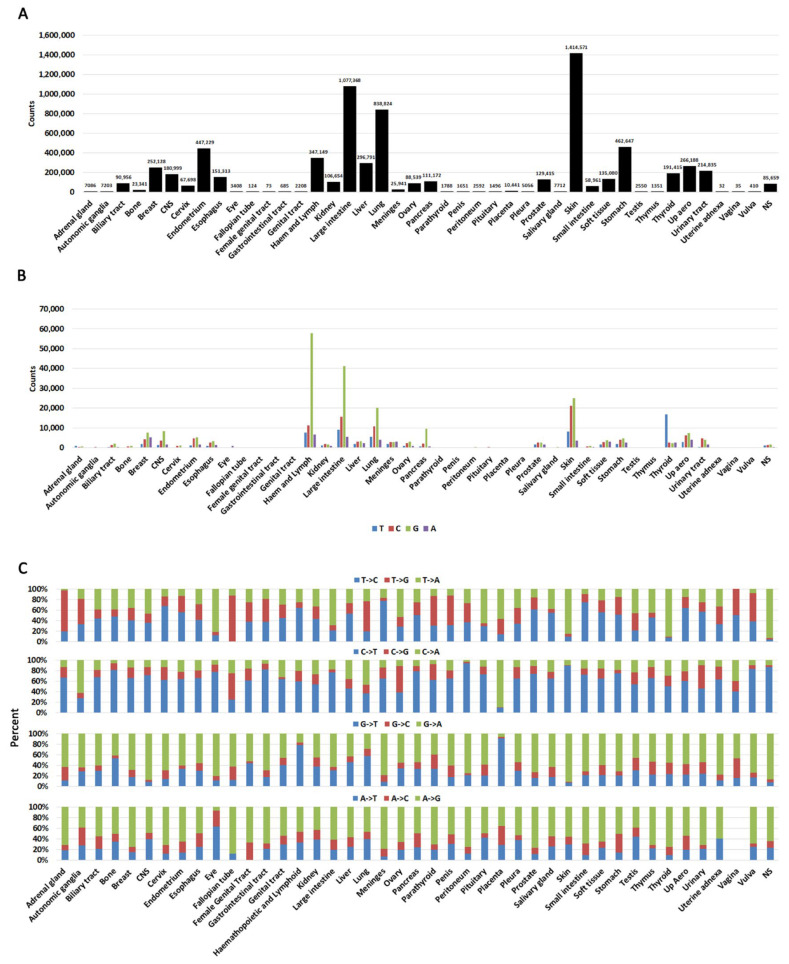
Cancer-specific distributions of mutations. (**A**). A histogram showing mutation counts queried in each cancer. Numbers on top of each bar represent the exact mutations queried in each cancer. Note that repeated mutations in multiple samples (hotspots) are also included in this graph. (**B**). The likelihood of a nucleotide being mutated in each cancer. (**C**). Percent substitutions from each nucleotide in each cancer. NS = not specified.

Although a pan-cancer analysis reveals general trends, it has a major caveat in that not all cancers are represented equally. More data has been deposited for some cancers than others, and this can skew the analysis in favor of more represented cancers. Additionally, each cancer may acquire mutation signatures by different mechanisms. We, therefore, parsed the data by cancer type. Indeed, this analysis shows that some cancers (e.g., large intestine, and skin) are much more represented than others (e.g., uterine adnexa) (Figure 2A, Supplementary Table S2A).

When we analyzed substitutions arising at any of the four nucleotides, we identified that G is the most mutated base with C being the second most likely (Figure 2B, Supplementary Table S2B). This is not unexpected because genes are GC rich. We next analyzed substitution type at each nucleotide (e.g., G->A, G->T, G->C, etc.) (Figure 2C, Supplementary Table S2C). This analysis shows that changes are not complementary (e.g., an equal percentage of G->A and C->T). An incorporation of complementary changes on both strands would be expected to result from replication errors. Conversely, processes such as transcriptional strand bias can result in higher levels of mutation on the transcribed strand compared to the non-transcribed strand [48].

Other strand bias mechanisms can also operate in cancer cells. For example, C->T transition chemical mechanisms have been well documented: they can occur by deamination of CpG islands [9,49], clock-like mutation signatures [39], and decreased polymerase delta processivity [50]. A specific analysis of nucleotide changes revealed some minor discrepancies between different cancer types (Figure 2C, Supplementary Table S2C), suggesting that some of these mechanisms may operate in some cancers. Certain cancers may also appear skewed towards specific mutations (e.g., autonomic ganglia, fallopian tube), but this is due to low sample number (Supplementary Table S2A). Vaginal cancer did not have any A->G transitions, but any meaningful interpretation of this observation should be taken with a caveat because only 35 samples were reported.

**Figure 3:**
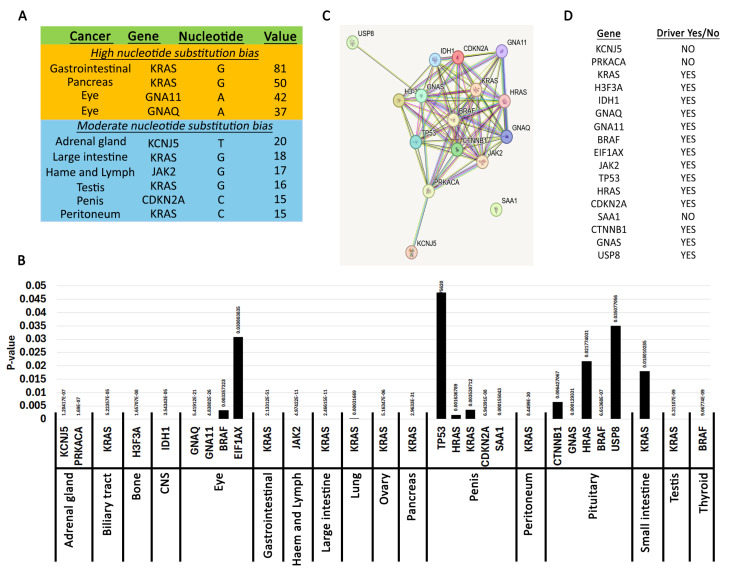
Pan-cancer biased nucleotide substitutions. (**A**). Genes with high and moderate nucleotide bias. Most significant nucleotide changes. The table shows high nucleotide substitution bias (over 30) and moderate (over 15) extracted using the per-cancer matrix analysis. Cancers with fewer than 500 samples were excluded from the table. Also, please see Materials and Methods for description of these analyses. (**B**). Genes with statistically significant nucleotide skews. The graph shows the genes in each cancer with a chi-square *p*-value below 0.05. Please see Materials and Methods for description of analysis. (**C**). Connections between the genes in A generated using the STRING database. (**D**). Driver status of genes identified in A as reported using Integrative Oncogenomics.

To investigate whether there are cancer-specific biased substitutions, we created heatmaps (Supplementary Figure S1, Supplementary Table S3). In the interest of consistency and accurate comparison across all cancer types, we implemented a normalization process that adjusted mutation counts based on reference bases and sample sizes. This approach allowed us to capture significant mutation patterns while minimizing the impact of lower-priority variations. We created matrices for each cancer from normalized data (see Materials and Methods and Supplementary Figure S1). This identified ten cancers with high and moderate biased nucleotide substitutions in certain genes (Figure 3A). As expected for *KRAS* there is a bias for substitutions to occur predominantly at the guanine nucleotide because of the signature G12D/V/R/C/A and G13D. In the gastrointestinal tract (exact location not specified), G12D accounted for 28%, G12V for 14.7%, and G13D for 30.7%. Substitution at G12 to either A, C, R, and S comprised 26% of the mutations. Only one sample had a different substitution (K117N). In samples where the site was specified (large intestine and small intestine), there was a similar, albeit more moderate, bias toward *KRAS* G12 mutations, indicating that other *KRAS* mutations were present. The pancreas also showed a mutation distribution bias towards G12 (46.2% G12D, 31.3% G12V, 12.5% G12R, 2.6%.

G12C, 1.2% G12A, 6.2% other substitutions at any nucleotide. There were no substitutions at the G13 residue in the pancreas. This data agrees with previous observations [51,52]. Moderate G12 *KRAS* nucleotide skews were also identified in the peritoneum and testis (Figure 3A, Supplementary Figure S1).

We also identified moderate bias substitutions in *KCNJ5* (adrenal gland), *JAK2* (hematopoietic and lymphoid), and *CDKN2A* (penis) (Figure 3A, Supplementary Figure S1). For *KCNJ5*, 53% of mutations were L168R (c.503T>G) and 42.8% were G151R (c.451G>C). *KCNJ5* encodes a potassium channel with high expression in the adrenal gland, and these two mutations have been identified in aldosterone-producing adenomas. Remarkably, the L168R mutation inhibits cell proliferation while the G151R has no effect [53]. In fact, they both appeared to induce apoptosis. Nevertheless, these are signature *KCNJ5* mutations in adrenal glands [54]. For *JAK2*, the bias is caused by the signature oncogenic V617F (c.1849G>T), which causes ligand-independent constitutive phosphorylation of the tyrosine kinase [55]. In the penis, two *CDKN2A* truncations (R58* c.172C>T and R80* c.238C>T) account for 44.2% and 20.9% of mutations, respectively. Another 20.9% is due to the H83Y (c.247C>T) mutation [56,57,58,59,60,61,62]. To our knowledge, these truncations in *CDKN2A* were not reported before. However, these penile mutations should be taken with a caveat because the sample size for penis cancer is low. Thus, it is hard to conclude whether a *CDKN2A* mutational bias exists in penile cancers.

### 2.3. Statistically Significant Mutations Yielding Biased Substitutions

We next performed a more comprehensive analysis to identify biased nucleotide substitutions. We use the chi-square test to identify statistically significant nucleotide substitution skews. The null hypothesis was that mutations should occur randomly throughout the region of a gene (Supplementary Table S4). This analysis immediately revealed the genes identified in Supplementary Figure S1 using heatmaps (Figure 3A). However, it also identified additional patterns in other cancers (Figure 3B). Guanine is the most frequently mutated nucleotide in these statistically significant skews (Supplementary Figure S2A). Guanine is substituted primarily to adenine and thymine (Supplementary Figure S2B,C). Mutations at cytidine did not occur with equal frequency with guanine but were similar in frequency with adenine. Substitutions involving the thymidine base were the rarest. Thus, it appears that nucleotide substitution skews are primarily generated by G->A transitions. An analysis of the coordinates of the statistically significant skews revealed precisely that (Table 1).

The guanine biases are primarily caused by mutations in Ras family GTPases involved in cell cycle-regulating signal transduction (Figure 3B). We used the STRING database to understand the relationship between the other genes identified and Ras family GTPases (Figure 3C). This analysis shows that most of the identified genes constitute a closely related set of cell cycle regulators. There were three “outliers” that were more distantly related. *USP8* was identified in the pituitary by having statistically significant enriched mutations at two residues (S718P/Y, P720Q/R) [63,64,65,66]. *USP8* is a ubiquitin ligase involved in degrading tyrosine kinase receptors including *EGFR* [67]. Thus, inactivating *USP8* mutations causes a gain of function phenotype by increasing or prolonging signaling from cell cycle-regulating receptors. *KCNJ5* was identified in the adrenal gland and is described in the previous section. *SAA1* had a pronounced mutation skew in penile cancers (c.230C>G, p.T77S). *SAA1* codes for a protein within the amyloid A family of apolipoproteins involved in fat metabolism, inflammation, and tissue injury [68,69]. Not unexpectedly, the gene is highly expressed in fat and liver tissues [70]. *SAA1* mutations have been identified in various cancers [69], but to our knowledge selection of the T77S mutation in penis cancers has not been well characterized. It was identified from characterization of several penis cancer cell lines [57], and remarkably all T77S substitutions were homozygous. *SAA1* is characterized by four alpha helixes and a C-terminal tail. The mutation was identified in the ENST00000405158.2 isoform, which has 122 residues and is the longest transcript. Single nucleotide polymorphisms have been identified at the T77 position (rs1671926), but the clinical significance of these mutations is uncertain. ClinVar reports that the alternate G allele at position c.230 is more predominant in Asian populations, and the penile cancer cell lines were of Asian origin [57]. However, as discussed above for *CDKN2A* mutations, it is hard to conclude whether *SAA1* mutations predispose patients to penile cancers because the sample size is too small. Thus, although this finding may be intriguing, more data is necessary to make any solid conclusions about the role of *SAA1*.

We used Integrative Oncogenomics to interrogate whether the nucleotide skews identified in this analysis occur within driver genes [16]. Indeed, we find that 14 out of the 17 genes identified are characterized as drivers (Figure 3D). Thus, we conclude that nucleotide substitution biases act as a purifying selection for mutations in driver cell cycle regulators. 

### 2.4. Genetic Correlations Between Cancers

**Figure 4:**
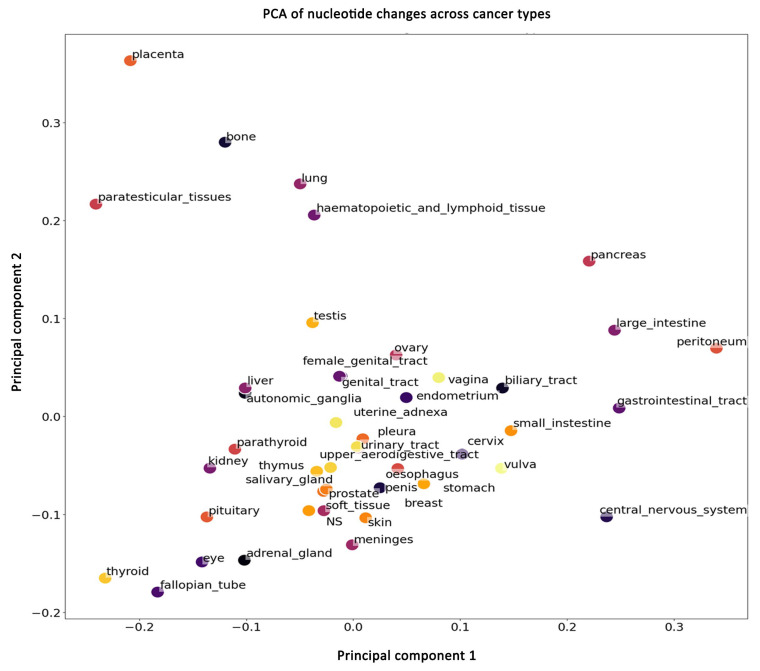
Principal component analysis (PCA) plot of nucleotide change patterns across various cancer types. The plot displays the first two principal components (PC1 and PC2), capturing the most significant variance in the mutation data. Each point represents a different cancer type, with proximity between points indicating similarity in mutation profiles.

To understand whether mutations at the nucleotide level can be actionable to identify correlations between different cancers, we use principal component analysis (PCA) (Figure 4). By applying PCA, the complex, high-dimensional data is reduced to just two principal components (PC1 and PC2), which capture the most significant variations in the mutation data. This reduction simplifies the visualization and analysis of the data, allowing us to observe patterns and clusters that might indicate similarities or differences in the genetic mutation profiles of different cancers.

We find that most cancers form a cluster, indicating that the nucleotide substitution changes are similar among these cancers. However, some cancers (placenta, bone, paratesticular, lung, hematopoietic, lymphoid, pancreas, large intestine, peritoneum, gastrointestinal, and central nervous system (CNS)) separate from this cluster, suggesting that these cancers have unique nucleotide substitution skews. The analysis presented here considers nucleotide substitutions in the 25 most frequently mutated genes and includes all substitutions, including rare mutations. Taken together, these data show that although most cancers display a similar nucleotide mutational spectrum, some could be differentiated even at nucleotide resolution. Generally, cancers have been classified by differences in the mutated genes, but here we show that nucleotide substitution differences can also be used to classify cancers. This suggests that for some cancers unique genomic mutational processes are at work.

## 3. Materials and Methods

### 3.1. Data Accession and Processing

Excel files with mutation data for 43 different cancer types and one non-specified were downloaded from the Catalogue of Somatic Mutations in Human Cancers (COSMIC) [38]. All files were first sorted by GENE_NAME. Then, non-substitution samples were removed according to the MUTATION_CDS column, and alternative transcripts of each gene were removed by filtering out trailing _ENST in the gene names. Finally, only those mutations occurring within coding regions were retained based on the MUTATION_AA column.

### 3.2. Nucleotide Substitution Parsing

For each cancer type, we first categorized the samples into 12 nucleotide mutation groups based on the ‘MUTATION_CDS’. Next, we used the value_counts function from the Pandas library in Python (version 3.0) to tally the occurrences of each gene name, identifying the top 25 most frequently mutated genes. We then iterated through these top 25 genes, counting their occurrences within each of the 12 mutation groups. The results were compiled into a matrix with rows representing the top 25 frequently mutated genes, columns representing the 12 nucleotide mutations, and the values indicating the count of each gene within each mutation group.

### 3.3. Gene Frequency Rank

To quantify mutation prevalence across cancers, the top 25 most frequently mutated genes in each cancer type were assigned rank values from 25 (highest frequency) to 1 (lowest frequency). These ranks were consolidated into a gene-by-cancer table and grouped by gene name. For each gene, rank values across cancers were summed to generate a cumulative pan-cancer rank representing its overall mutation frequency. The full ranked list parsed by cancer type is provided in Supplementary Table S1A, and the cumulative ranks (scaled by dividing by 10 for visualization only) are shown in Figure 1A.

To summarize nucleotide usage across these frequently mutated genes, we calculated pan-cancer nucleotide alteration frequencies by dividing the number of mutations at each reference base (T, C, G, A) by the total number of mutations across all bases (463,968). For example, 70,030 mutations at T correspond to 15.9% of all observed mutations. These distributions are shown in Figure 1B. The total counts of each substitution type from each reference base (e.g., T->C, T->G, T->A) are reported in Figure 1C, and their percentages relative to all substitutions from each reference base are summarized in Figure 1D.

### 3.4. Normalization

To correct for differences in cohort size and nucleotide abundance, mutation counts were normalized in two steps. First, for each cancer type, counts of each substitution (e.g., T->C, T->G, T->A) were divided by the total number of mutations observed at that reference nucleotide within that cancer. This adjusts for unequal frequencies of T, C, G, and A in coding regions. Second, these values were divided by the total number of samples in that cancer type, so that cancers with very large cohorts do not artificially appear to have stronger mutation biases. The distribution of sample sizes used in this normalization is shown in Supplementary Figure S2A.

### 3.5. Heatmaps

To analyze mutation patterns in both individual cancers (per-cancer) and across multiple cancer types (pan-cancer), we created heatmaps—visual representations that illustrate the intensity of mutations for each gene. Cancers with fewer than 500 samples were excluded from this analysis because small cohorts produce unstable mutation-rate estimates with high variance and sparse substitution counts, making heatmap comparison unreliable. A Python code was developed to process and visualize the heatmaps, focusing on identifying mutations that exceed a specified threshold.

The analysis begins by importing normalized mutation data, with each dataset corresponding to a different cancer type. For each dataset, a heatmap is generated to visualize the mutation patterns, with genes on the *y*-axis and nucleotide changes on the *x*-axis. Heatmaps were created for each cancer type separately (Supplementary Figure S2). To identify meaningful mutation enrichments in the heatmaps (Supplementary Figure S1) without overwhelming visual noise, we evaluated multiple cutoffs (1, 3, 5, 8, 10). A threshold of 5 produced the clearest and most biologically interpretable patterns across all cancers. Thus, the threshold of 5 was selected empirically to optimize signal-to-noise representation. 

### 3.6. Chi-Square Calculations of Nucleotide Bias

A chi-square test was used to calculate statistically significant nucleotide biases. The calculations were conducted on normalized data (Supplementary Table S4, columns J–M). Because the chi-square test does not work well with small values, the normalized values were multiplied by 10^6^ to make them larger (see columns N, O, P, Q, Supplementary Table S4). These adjusted larger values are the observed values. The expected values were calculated based on the observed values (see columns R, S, T, U). The null hypothesis here would be that each nucleotide should be hit equally (e.g., mutation should occur with equal probability in every residue within the coding region). The chi-square value was calculated (column W), and the chi-square probability value was extracted using an online calculator from here: https://www.socscistatistics.com/pvalues/chidistribution.aspx (accessed on 1 September 2025). The *p*-values were extracted at 3 degrees of freedom since there are four values (Supplementary Table S4, column X). Statistically significant *p*-values (<0.05) are presented in Supplementary Table S3A. Supplementary Table S3B lists coordinates of genes, and mutations are in Supplementary Table S3A.

### 3.7. STRING Database

The STRING database (https://string-db.org) (accessed on 23 October 2024) was used to extract connections between the statistically significant genes in Figure 3C. The parameters used were network type-full STRING; meaning of network edges-evidence; active interaction sources-textmining, experiments, databases, co-expression, neighborhood, gene fusion, co-occurrence.

### 3.8. Gene Driver Potential

Integrative oncogenomics (https://www.intogen.org/search) (23 October 2024) [16] was used to determine whether the gene was classified as a driver.

### 3.9. Principal Component Analysis 

We aimed to plot the cancers in a single figure to compare the distribution of mutation types among frequently mutated genes. To achieve this, we aggregated the top 25 frequently mutated genes from each cancer type, resulting in a list of 373 identical genes. We then counted the mutation distribution for these 373 genes following the same procedure as described in the “Nucleotide Substitution Parsing” section. This produced a matrix for each cancer, where the rows represent the 373 genes, the columns represent 12 mutation groups, and the values are the mutation counts. To mitigate biases arising from the unequal distribution of T, C, G, and A in gene sequences and the varying number of samples across cancer types, we normalized the counts by dividing them by the corresponding gene name and cancer type counts. One gene was removed due to a lack of sequence information. Before plotting, we aggregated all the matrices into a single matrix by summing the counts from all the frequently mutated genes into a vector representing each cancer. Each vector contained 12 values corresponding to the mutation groups, and all vectors were compiled into a large matrix.

Finally, we standardized each row using L1 normalization and applied principal component analysis (PCA). PCA is a statistical technique that simplifies complex datasets by reducing their dimensions while preserving as much information as possible. In this case, it allowed us to compress the data into two dimensions, making it easier to visualize and compare the mutation patterns across different cancer types. The PCA plot was generated using Python and is shown in Figure 4.

## 4. Conclusions

Here we wanted to know whether nucleotide substitution patterns could be used to differentiate between cancers. We decided to analyze only the 25 most frequently mutated genes to eliminate noise from rare mutations. Our analysis shows that certain cancers are clearly biased by key signatures (e.g., *KRAS* G12 substitutions). Most importantly, we show that this analysis can quickly identify a close knit of key driver cell cycle regulators. Therefore, nucleotide substitution bias can be used in high-throughput genomics as a preliminary diagnostic tool and to classify certain cancers.

We are cognizant of the limitations of this study, including the fact that certain cancers do not have enough data to analyze. As genome sequencing becomes less expensive and more common, our data show that it may be possible to quickly diagnose cancers using raw sequencing data.

## Figures and Tables

**Table 1:** Coordinates of statistically significant mutations producing nucleotide skews.

Cancer	Gene	Mutation AA ^1^	Mutation CDS ^2^	Cancer	Gene	Mutation AA	Mutation CDS	Cancer	Gene	Mutation AA	Mutation CDS
Adrenal gland	KCNJ5	G151R	c.451G>A	Large intestine	KRAS	G12A/D/V	c.35G>C/A/T	Penis	TP53	P72R	c.215C>G
		L168R	c.503T>G			G12C/R/S	c.34G>T/C/A			R175H	c.524G>A
		E145Q	c.433G>C			G13A/D/V	c.38G>C/A/T			R248Q	c.743G>A
		T158A	c.472A>G			G13C/R/S	c.37G>T/C/A			R273H	c.818G>A
	PRKACA	L206R	c.617T>G			K117N	c.351A>T		HRAS	G12D	c.35G>A
Biliary tract	KRAS	G12A/D/V	c.35G>C/A/T			K117R	c.350A>G			G12S	c.34G>A
		G12C/R/S	c.34G>T/C/A			L19F	c.57G>C			G13R/S	c.37G>C/A
		G13C/R/S	c.37G>T/C/A			Q22K	c.64C>A			G13V	c.38G>T
		G13D	c.38G>A			Q22R	c.65A>G		KRAS	G12D	c.35G>A
		Q61H	c.183A>C			Q61E/K	c.181C>G/A			G12C/S	c.34G>T/A
Bone	H3F3A	G35W	c.103G>T			Q61L/P/R	c.182A>T/C/G		CDKN2A	H83Y	c.247C>T
CNS ^3^	IDH1	R132H/L/S	c.395G>A/T			Q61H	c.183A>C		SAA1	R58*	c.172C>T
		R132C/G/S	c.394C>T/G/A			R68S	c.204G>C			R80*	c.238C>T
		V178I	c.532G>A			V14I	c.40G>A			T77S	c.230C>G
Eye	GNAQ	Q209K	c.625C>A	Lung	KRAS	G12A/D/V	c.35G>C/A/T	Pituitary	CTNNB1	D32H/V/Y	c.95A>G/T
		Q209L/P/R	c.626A>T/C/G			G12C/R/S	c.34G>T/C/A			D32H/N/Y	c.94G>C/A/T
		Q209H	c.627A>C			G13A/D/V	c.38G>C/A/T			G34R	c.100G>C
		R183Q	c.548G>A			G13C/R/S	c.37G>T/C/A			G34V	c.101G>T
	GNA11	R209L/P/R	c.626A>T/C/G			L19F	c.57G>C			S33A/F/P	c.97T>G/C
		R209H	c.627G>T			Q61K	c.181C>A			S33C/F/Y	c.98C>G/T/A
		R183C	c.547C>T			Q61L/P/R	c.182A>T/C/G			T41I	c.122C>T
	BRAF	V600E	c.1799T>A			Q61H	c.183A>C		GNAS	Q870L/R	c.2609A>T/G
Gastro-intestinal tract	KRAS	G12A/D/V	c.35G>C/A/T	Ovary	KRAS	G12A/D/V	c.35G>C/A/T			R844C/S	c.2530C>T/A
		G12C/R/S	c.34G>T/C/A			G12C/R/S	c.34G>T/C/A			R844H	c.2531G>A
		G13D	c.38G>A			G13A/D/V	c.38G>C/A/T		HRAS	G12V	c.35G>T
Haem and lymph ^4^	JAK2	V617F/I	c.1849G>T/A			G13C/R/S	c.37G>T/C/A			G12R	c.34G>C
		R683G	c.2047A>G			Q61L	c.182A>T		BRAF	V600E	c.1799T>A
		R683K	c.2048G>A			Q61H	c.183A>C		USP8	P720Q/R	c.2159C>A/G
		R683S	c.2049A>T	Pancreas	KRAS	G12A/D/V	c.35G>C/A/T			S718P	c.2152T>C
		R867Q	c.2600G>A			G12C/R/S	c.34G>T/C/A			S718Y	c.2153C>A
		R867W	c.2599C>T			G13A/D/V	c.38G>C/A/T	Testis	KRAS	G12A/D/V	c.35G>C/A/T
		R938Q	c.2813G>A			G13C/R/S	c.37G>T/C/A			G12C/R/S	c.34G>T/C/A
		T875N	c.2624C>A			Q61K	c.181C>A	Thyroid	BRAF	V600E	c.1799T>A
		G571S	c.1711G>A			Q61L/P/R	c.182A>T/C/G			E26A	c.77A>C
		I682F	c.2044A>T			Q61H	c.183A>C			K601E	c.1801A>G
Small intestine	KRAS	G12A/D/V	c.35G>C/A/T	Peritoneum	KRAS	G12A/D/V	c.35G>C/A/T			S36A	c.106T>G
		G12C/R/S	c.34G>T/C/A			G12C/R/S	c.34G>T/C/A				
		G13D/V	c.38G>A/T			G13D	c.38G>A				
		G13C	c.37G>T			G13C/R	c.37G>T/C				

^1^ AA = amino acid; ^2^ CDS = coding DNA sequence; ^3^ CNS = central nervous system ^4^ Haem and Lymph = hematopoietic and lymphoid.

## Data Availability

All data presented here are available in public databases, COSMIC (https://cancer.sanger.ac.uk/cosmic) and cBioPortal (https://www.cbioportal.org/) (both accessed on 24 October 2024). These repositories anonymize all patient data and make it freely available to investigators. No consent is required to access or use the data.

## Supplementary Materials

The following supporting information can be downloaded at: https://www.mdpi.com/article/10.3390/ijms262411903/s1.
