# Supplementary material for: Nucleotide Substitution Biases in Related Cancer Driver Genes
Source: Int J Mol Sci. 2025 Dec 10;26(24):11903. doi: 10.3390/ijms262411903 (PMC12732676; doi:10.3390/ijms262411903)
Supplement: Supplementary file 1 [file ijms-26-11903-s001.zip › Supplementary Figure S1.pdf]

Supplementary Figure S1. Nucleotide mutation patterns separated by cancer type. The figures show matrices for the likelihood of changes at the four nucleotides in each cancer type. See Materials and Methods for calculations

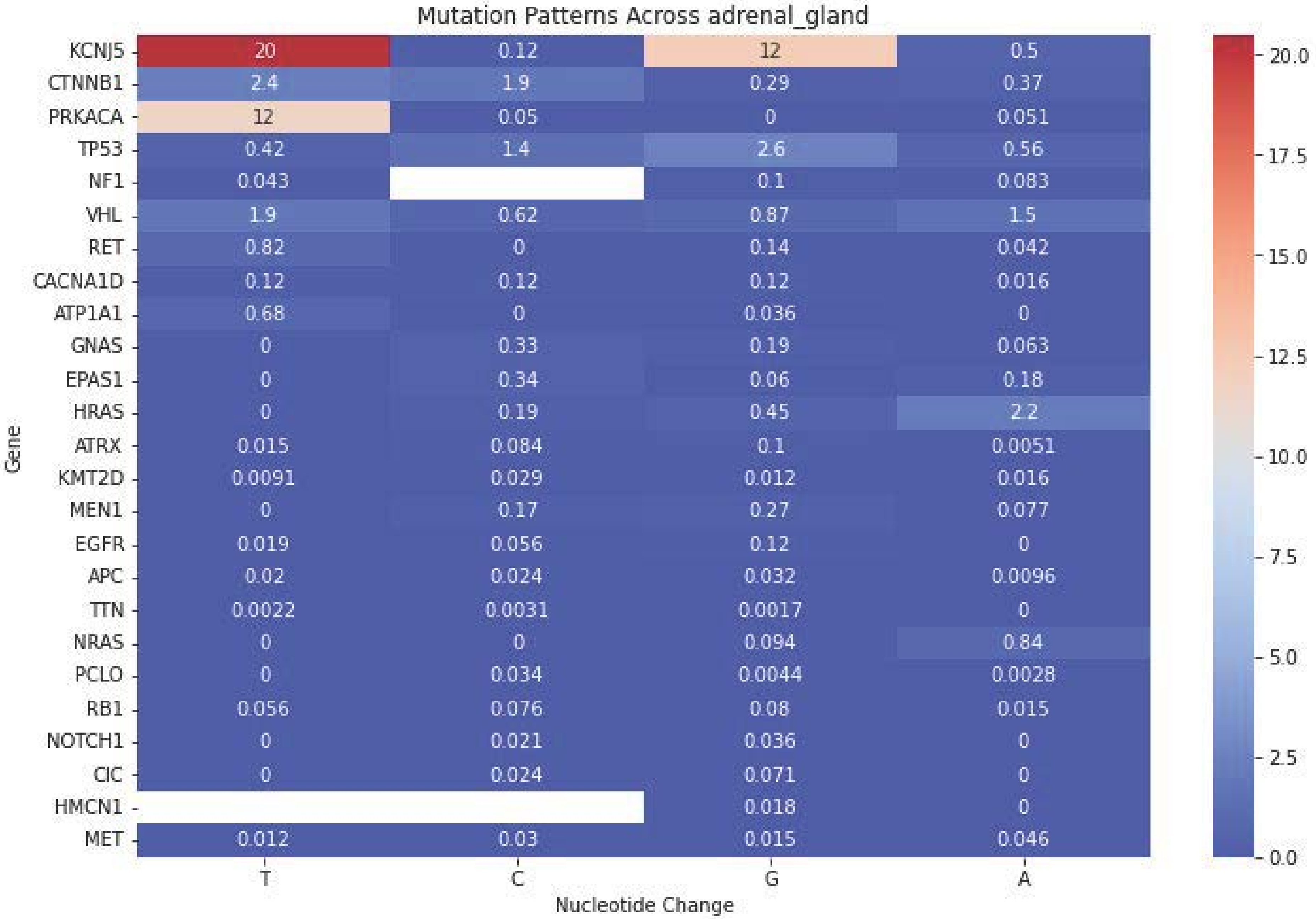

Mutation Patterns Across adrenal_gland
Gene
Nucleotide Change
T
C
G
A
KCNJ5
CTNNB1
PRKACA
TP53
NF1
VHL
RET
CACNA1D
ATP1A1
GNAS
EPAS1
HRAS
ATRX
KMT2D
MEN1
EGFR
APC
TTN
NRAS
PCLO
RB1
NOTCH1
CIC
HMCN1
MET
20 0.12 12 0.5
2.4 1.9 0.29 0.37
12 0.05 0 0.051
0.42 1.4 2.6 0.56
0.043 0.1 0.083
1.9 0.62 0.87 1.5
0.82 0 0.14 0.042
0.12 0.12 0.12 0.016
0.68 0 0.036 0
0 0.33 0.19 0.063
0 0.34 0.06 0.18
0 0.19 0.45 2.2
0.015 0.084 0.1 0.0051
0.0091 0.029 0.012 0.016
0 0.17 0.27 0.077
0.019 0.056 0.12 0
0.02 0.024 0.032 0.0096
0.0022 0.0031 0.0017 0
0 0 0.094 0.84
0 0.034 0.0044 0.0028
0.056 0.076 0.08 0.015
0 0.021 0.036 0
0 0.024 0.071 0
0.018 0
0.012 0.03 0.015 0.046
20.0
17.5
15.0
12.5
10.0
7.5
5.0
2.5
0.0

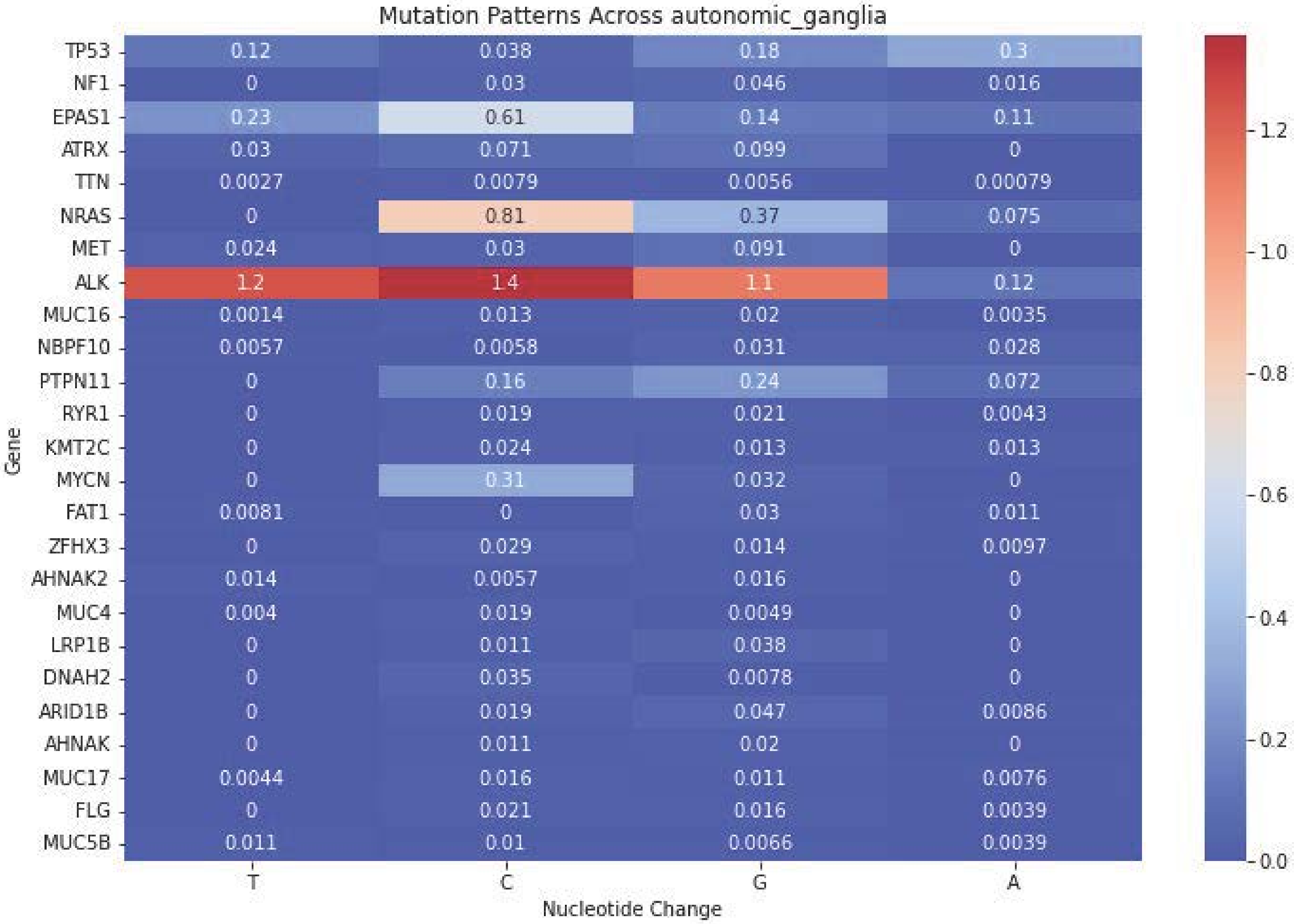

Mutation Patterns Across autonomic_ganglia
Gene
Nucleotide Change
T
C
G
A
TP53 0.12 0.038 0.18 0.3
NF1 0 0.03 0.046 0.016
EPAS1 0.23 0.61 0.14 0.11
ATRX 0.03 0.071 0.099 0
TTN 0.0027 0.0079 0.0056 0.00079
NRAS 0 0.81 0.37 0.075
MET 0.024 0.03 0.091 0
ALK 1.2 1.4 1.1 0.12
MUC16 0.0014 0.013 0.02 0.0035
NBPF10 0.0057 0.0058 0.031 0.028
PTPN11 0 0.16 0.24 0.072
RYR1 0 0.019 0.021 0.0043
KMT2C 0 0.024 0.013 0.013
MYCN 0 0.31 0.032 0
FAT1 0.0081 0 0.03 0.011
ZFHX3 0 0.029 0.014 0.0097
AHNAK2 0.014 0.0057 0.016 0
MUC4 0.004 0.019 0.0049 0
LRP1B 0 0.011 0.038 0
DNAH2 0 0.035 0.0078 0
ARID1B 0 0.019 0.047 0.0086
AHNAK 0 0.011 0.02 0
MUC17 0.0044 0.016 0.011 0.0076
FLG 0 0.021 0.016 0.0039
MUC5B 0.011 0.01 0.0066 0.0039
0.0
0.2
0.4
0.6
0.8
1.0
1.2

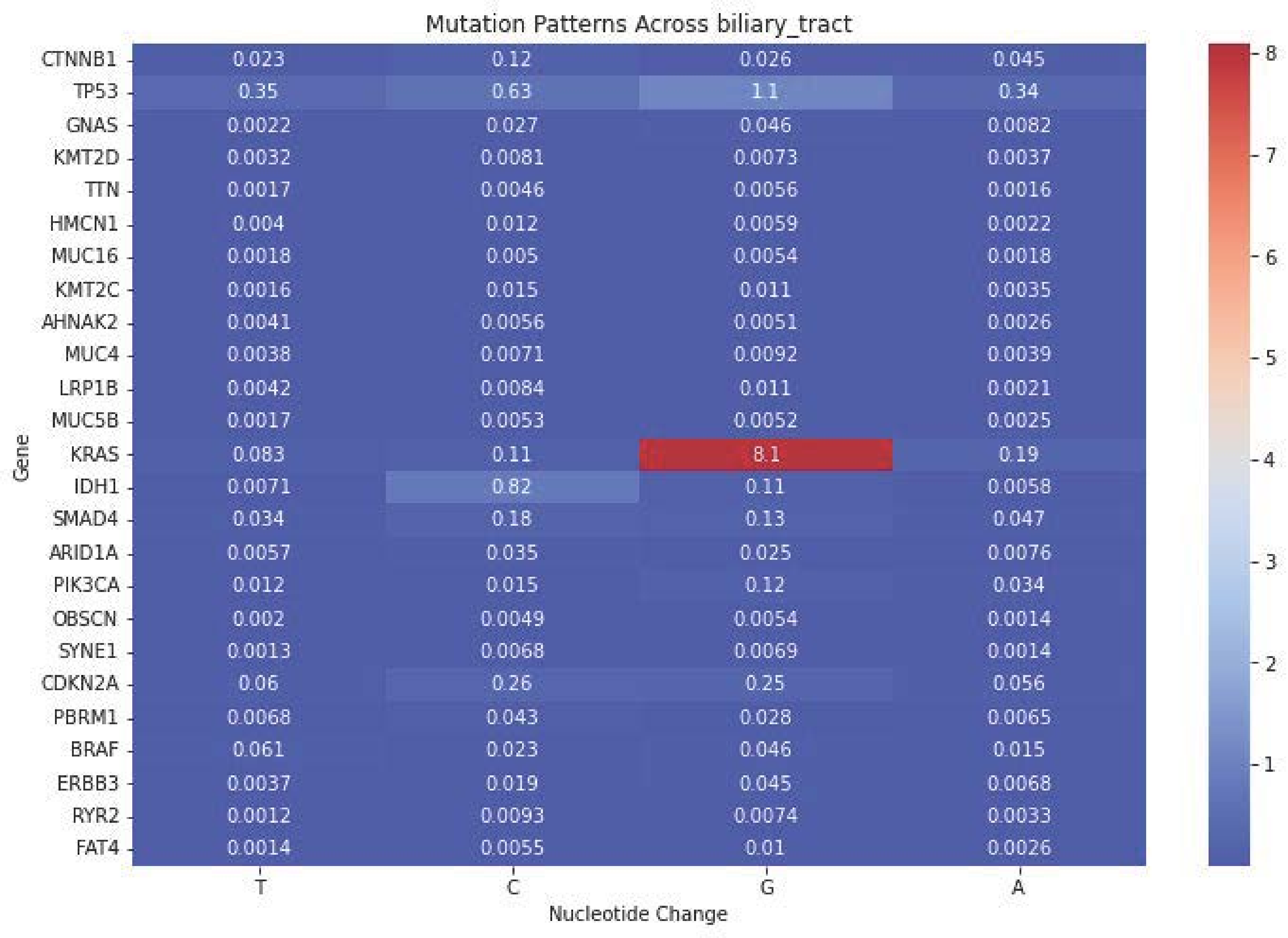

Mutation Patterns Across biliary_tract
Gene
Nucleotide Change
T
C
G
A
CTNNB1
TP53
GNAS
KMT2D
TTN
HMCN1
MUC16
KMT2C
AHNAK2
MUC4
LRP1B
MUC5B
KRAS
IDH1
SMAD4
ARID1A
PIK3CA
OBSCN
SYNE1
CDKN2A
PBRM1
BRAF
ERBB3
RYR2
FAT4
0.023 0.12 0.026 0.045
0.35 0.63 1.1 0.34
0.0022 0.027 0.046 0.0082
0.0032 0.0081 0.0073 0.0037
0.0017 0.0046 0.0056 0.0016
0.004 0.012 0.0059 0.0022
0.0018 0.005 0.0054 0.0018
0.0016 0.015 0.011 0.0035
0.0041 0.0056 0.0051 0.0026
0.0038 0.0071 0.0092 0.0039
0.0042 0.0084 0.011 0.0021
0.0017 0.0053 0.0052 0.0025
0.083 0.11 8.1 0.19
0.0071 0.82 0.11 0.0058
0.034 0.18 0.13 0.047
0.0057 0.035 0.025 0.0076
0.012 0.015 0.12 0.034
0.002 0.0049 0.0054 0.0014
0.0013 0.0068 0.0069 0.0014
0.06 0.26 0.25 0.056
0.0068 0.043 0.028 0.0065
0.061 0.023 0.046 0.015
0.0037 0.019 0.045 0.0068
0.0012 0.0093 0.0074 0.0033
0.0014 0.0055 0.01 0.0026
8
7
6
5
4
3
2
1

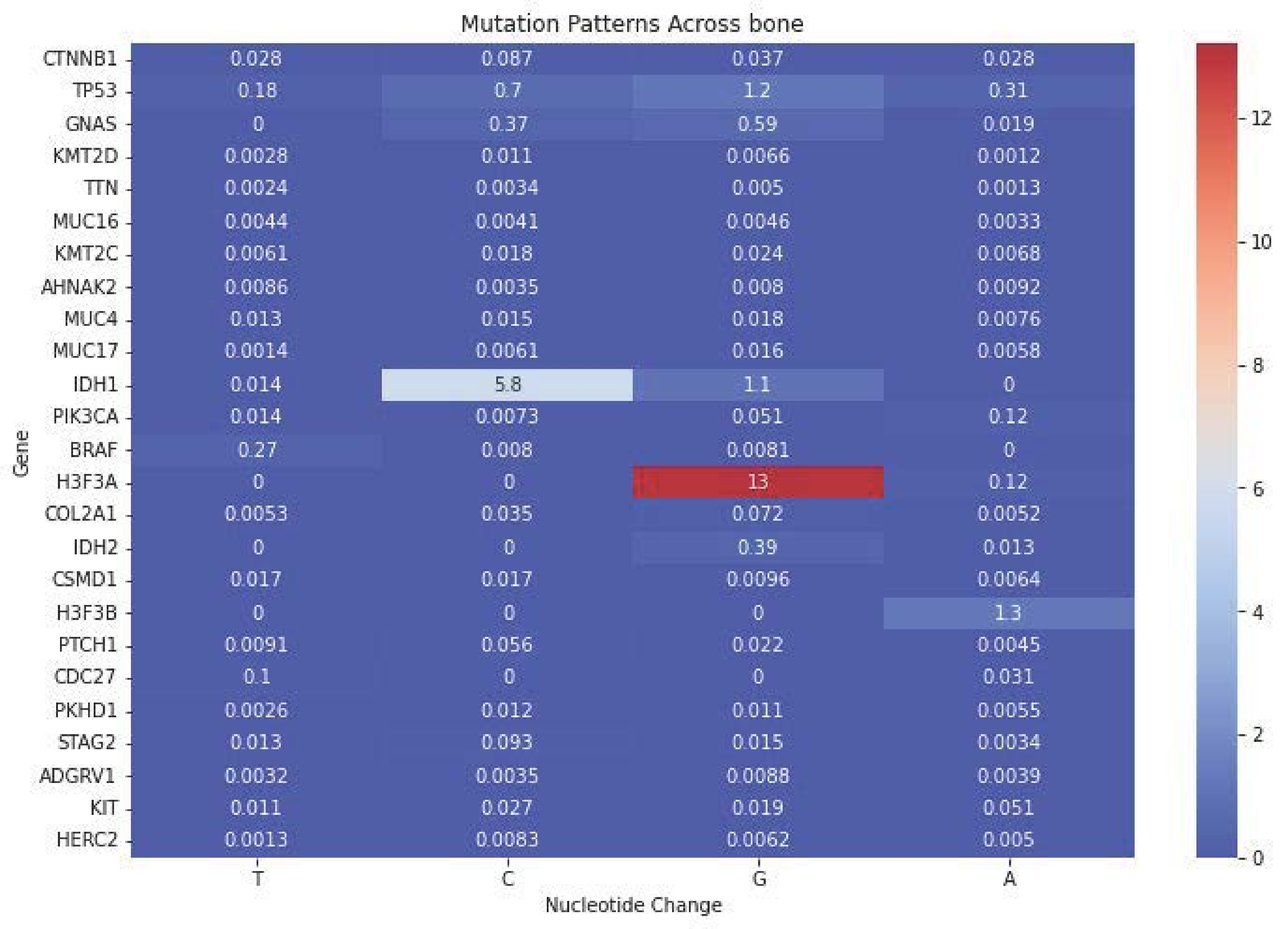
Mutation Patterns Across bone
Gene
Nucleotide Change
T
C
G
A
CTNNB1
TP53
GNAS
KMT2D
TTN
MUC16
KMT2C
AHNAK2
MUC4
MUC17
IDH1
PIK3CA
BRAF
H3F3A
COL2A1
IDH2
CSMD1
H3F3B
PTCH1
CDC27
PKHD1
STAG2
ADGRV1
KIT
HERC2
0.028 0.087 0.037 0.028
0.18 0.7 1.2 0.31
0 0.37 0.59 0.019
0.0028 0.011 0.0066 0.0012
0.0024 0.0034 0.005 0.0013
0.0044 0.0041 0.0046 0.0033
0.0061 0.018 0.024 0.0068
0.0086 0.0035 0.008 0.0092
0.013 0.015 0.018 0.0076
0.0014 0.0061 0.016 0.0058
0.014 5.8 1.1 0
0.014 0.0073 0.051 0.12
0.27 0.008 0.0081 0
0 0 13 0.12
0.0053 0.035 0.072 0.0052
0 0 0.39 0.013
0.017 0.017 0.0096 0.0064
0 0 0 1.3
0.0091 0.056 0.022 0.0045
0.1 0 0 0.031
0.0026 0.012 0.011 0.0055
0.013 0.093 0.015 0.0034
0.0032 0.0035 0.0088 0.0039
0.011 0.027 0.019 0.051
0.0013 0.0083 0.0062 0.005
12
10
8
6
4
2
0

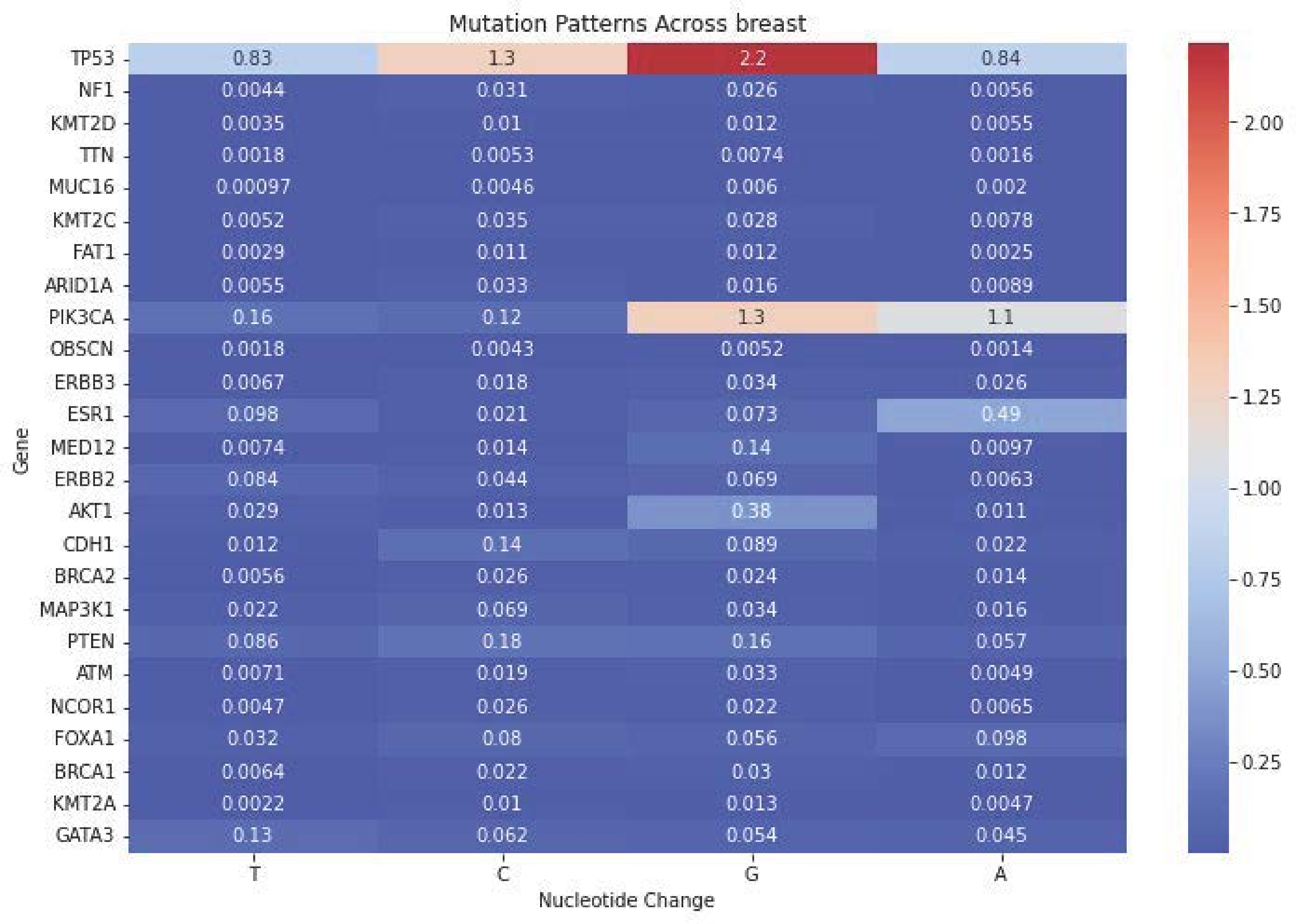
Mutation Patterns Across breast
Gene
Nucleotide Change
T
C
G
A
TP53 0.83 1.3 2.2 0.84
NF1 0.0044 0.031 0.026 0.0056
KMT2D 0.0035 0.01 0.012 0.0055
TTN 0.0018 0.0053 0.0074 0.0016
MUC16 0.00097 0.0046 0.006 0.002
KMT2C 0.0052 0.035 0.028 0.0078
FAT1 0.0029 0.011 0.012 0.0025
ARID1A 0.0055 0.033 0.016 0.0089
PIK3CA 0.16 0.12 1.3 1.1
OBSCN 0.0018 0.0043 0.0052 0.0014
ERBB3 0.0067 0.018 0.034 0.026
ESR1 0.098 0.021 0.073 0.49
MED12 0.0074 0.014 0.14 0.0097
ERBB2 0.084 0.044 0.069 0.0063
AKT1 0.029 0.013 0.38 0.011
CDH1 0.012 0.14 0.089 0.022
BRCA2 0.0056 0.026 0.024 0.014
MAP3K1 0.022 0.069 0.034 0.016
PTEN 0.086 0.18 0.16 0.057
ATM 0.0071 0.019 0.033 0.0049
NCOR1 0.0047 0.026 0.022 0.0065
FOXA1 0.032 0.08 0.056 0.098
BRCA1 0.0064 0.022 0.03 0.012
KMT2A 0.0022 0.01 0.013 0.0047
GATA3 0.13 0.062 0.054 0.045
2.00
1.75
1.50
1.25
1.00
0.75
0.50
0.25

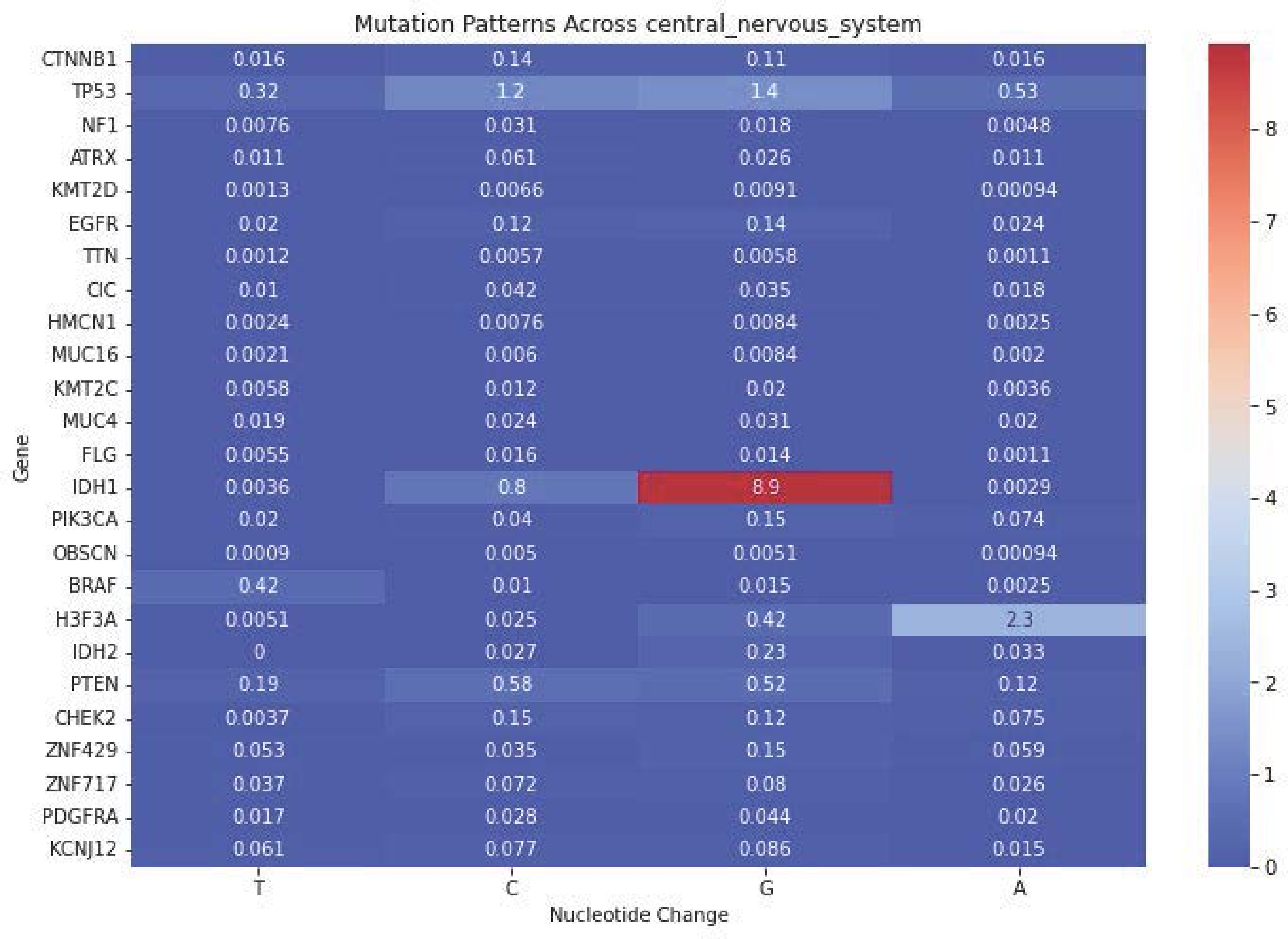
Mutation Patterns Across central_nervous_system
Gene
Nucleotide Change
T
C
G
A
CTNNB1 0.016 0.14 0.11 0.016
TP53 0.32 1.2 1.4 0.53
NF1 0.0076 0.031 0.018 0.0048
ATRX 0.011 0.061 0.026 0.011
KMT2D 0.0013 0.0066 0.0091 0.00094
EGFR 0.02 0.12 0.14 0.024
TTN 0.0012 0.0057 0.0058 0.0011
CIC 0.01 0.042 0.035 0.018
HMCN1 0.0024 0.0076 0.0084 0.0025
MUC16 0.0021 0.006 0.0084 0.002
KMT2C 0.0058 0.012 0.02 0.0036
MUC4 0.019 0.024 0.031 0.02
FLG 0.0055 0.016 0.014 0.0011
IDH1 0.0036 0.8 8.9 0.0029
PIK3CA 0.02 0.04 0.15 0.074
OBSCN 0.0009 0.005 0.0051 0.00094
BRAF 0.42 0.01 0.015 0.0025
H3F3A 0.0051 0.025 0.42 2.3
IDH2 0 0.027 0.23 0.033
PTEN 0.19 0.58 0.52 0.12
CHEK2 0.0037 0.15 0.12 0.075
ZNF429 0.053 0.035 0.15 0.059
ZNF717 0.037 0.072 0.08 0.026
PDGFRA 0.017 0.028 0.044 0.02
KCNJ12 0.061 0.077 0.086 0.015
0
1
2
3
4
5
6
7
8

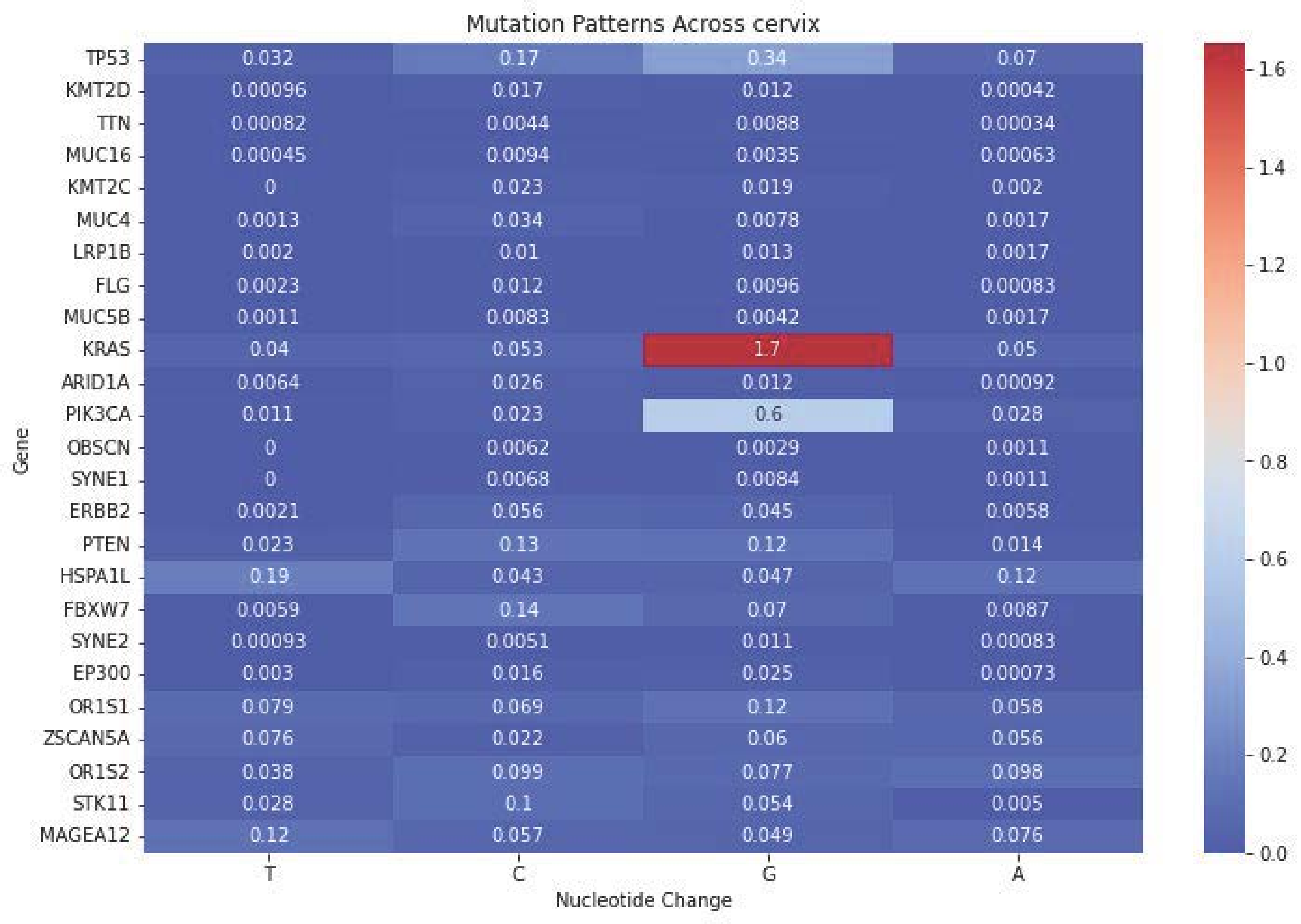
Mutation Patterns Across cervix
Gene
Nucleotide Change
T
C
G
A
TP53 0.032 0.17 0.34 0.07
KMT2D 0.00096 0.017 0.012 0.00042
TTN 0.00082 0.0044 0.0088 0.00034
MUC16 0.00045 0.0094 0.0035 0.00063
KMT2C 0 0.023 0.019 0.002
MUC4 0.0013 0.034 0.0078 0.0017
LRP1B 0.002 0.01 0.013 0.0017
FLG 0.0023 0.012 0.0096 0.00083
MUC5B 0.0011 0.0083 0.0042 0.0017
KRAS 0.04 0.053 1.7 0.05
ARID1A 0.0064 0.026 0.012 0.00092
PIK3CA 0.011 0.023 0.6 0.028
OBSCN 0 0.0062 0.0029 0.0011
SYNE1 0 0.0068 0.0084 0.0011
ERBB2 0.0021 0.056 0.045 0.0058
PTEN 0.023 0.13 0.12 0.014
HSPA1L 0.19 0.043 0.047 0.12
FBXW7 0.0059 0.14 0.07 0.0087
SYNE2 0.00093 0.0051 0.011 0.00083
EP300 0.003 0.016 0.025 0.00073
OR1S1 0.079 0.069 0.12 0.058
ZSCAN5A 0.076 0.022 0.06 0.056
OR1S2 0.038 0.099 0.077 0.098
STK11 0.028 0.1 0.054 0.005
MAGEA12 0.12 0.057 0.049 0.076
1.6
1.4
1.2
1.0
0.8
0.6
0.4
0.2
0.0

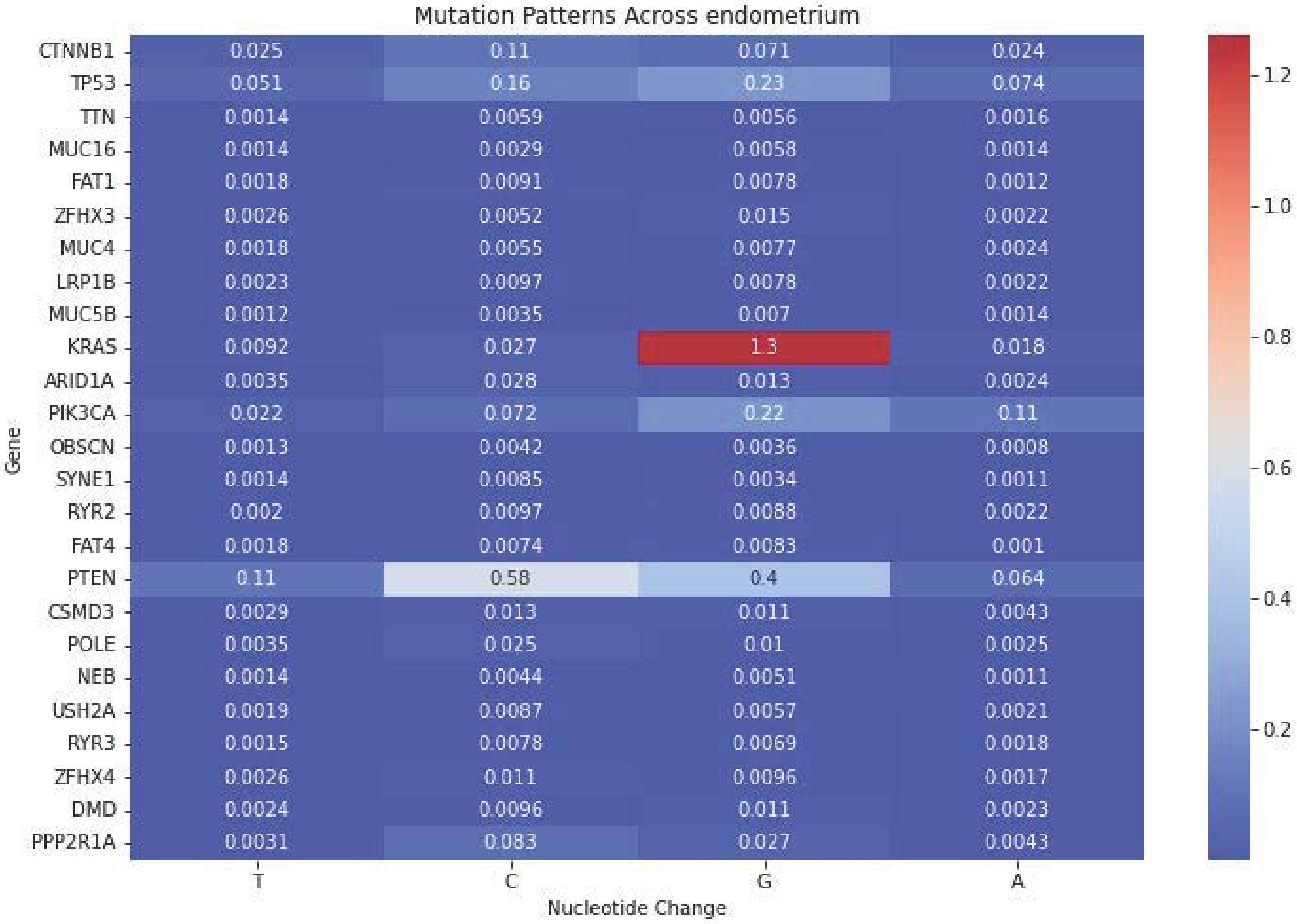
Mutation Patterns Across endometrium
Gene
CTNNB1
TP53
TTN
MUC16
FAT1
ZFHX3
MUC4
LRP1B
MUC5B
KRAS
ARID1A
PIK3CA
OBSCN
SYNE1
RYR2
FAT4
PTEN
CSMD3
POLE
NEB
USH2A
RYR3
ZFHX4
DMD
PPP2R1A
T
C
G
A
Nucleotide Change
0.025 0.11 0.071 0.024
0.051 0.16 0.23 0.074
0.0014 0.0059 0.0056 0.0016
0.0014 0.0029 0.0058 0.0014
0.0018 0.0091 0.0078 0.0012
0.0026 0.0052 0.015 0.0022
0.0018 0.0055 0.0077 0.0024
0.0023 0.0097 0.0078 0.0022
0.0012 0.0035 0.007 0.0014
0.0092 0.027 1.3 0.018
0.0035 0.028 0.013 0.0024
0.022 0.072 0.22 0.11
0.0013 0.0042 0.0036 0.0008
0.0014 0.0085 0.0034 0.0011
0.002 0.0097 0.0088 0.0022
0.0018 0.0074 0.0083 0.001
0.11 0.58 0.4 0.064
0.0029 0.013 0.011 0.0043
0.0035 0.025 0.01 0.0025
0.0014 0.0044 0.0051 0.0011
0.0019 0.0087 0.0057 0.0021
0.0015 0.0078 0.0069 0.0018
0.0026 0.011 0.0096 0.0017
0.0024 0.0096 0.011 0.0023
0.0031 0.083 0.027 0.0043
1.2
1.0
0.8
0.6
0.4
0.2

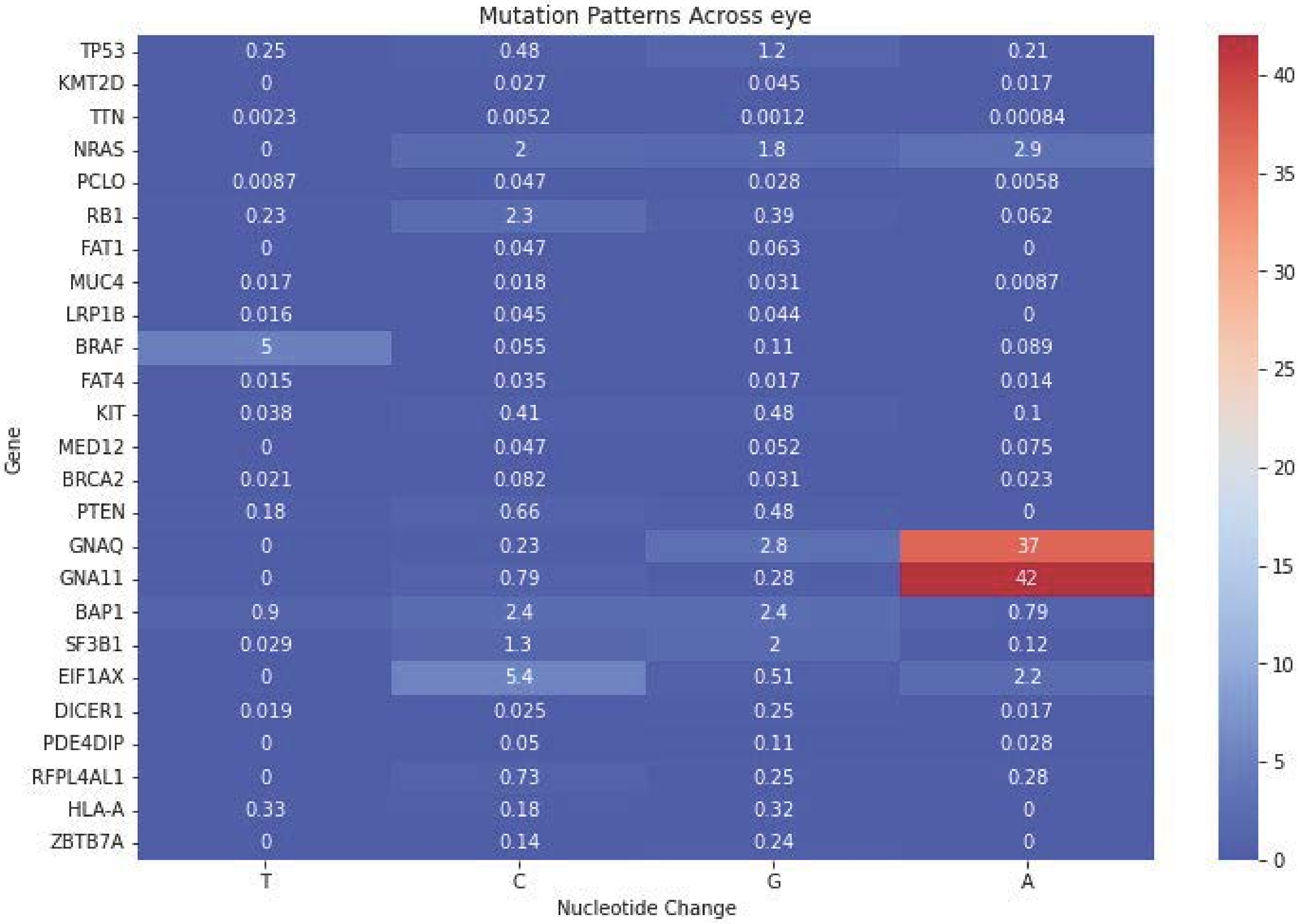

Mutation Patterns Across eye
Gene
Nucleotide Change
T
C
G
A
TP53
KMT2D
TTN
NRAS
PCLO
RB1
FAT1
MUC4
LRP1B
BRAF
FAT4
KIT
MED12
BRCA2
PTEN
GNAQ
GNA11
BAP1
SF3B1
EIF1AX
DICER1
PDE4DIP
RFPL4AL1
HLA-A
ZBTB7A
0.25 0.48 1.2 0.21
0 0.027 0.045 0.017
0.0023 0.0052 0.0012 0.00084
0 2 1.8 2.9
0.0087 0.047 0.028 0.0058
0.23 2.3 0.39 0.062
0 0.047 0.063 0
0.017 0.018 0.031 0.0087
0.016 0.045 0.044 0
5 0.055 0.11 0.089
0.015 0.035 0.017 0.014
0.038 0.41 0.48 0.1
0 0.047 0.052 0.075
0.021 0.082 0.031 0.023
0.18 0.66 0.48 0
0 0.23 2.8 37
0 0.79 0.28 42
0.9 2.4 2.4 0.79
0.029 1.3 2 0.12
0 5.4 0.51 2.2
0.019 0.025 0.25 0.017
0 0.05 0.11 0.028
0 0.73 0.25 0.28
0.33 0.18 0.32 0
0 0.14 0.24 0
0
5
10
15
20
25
30
35
40

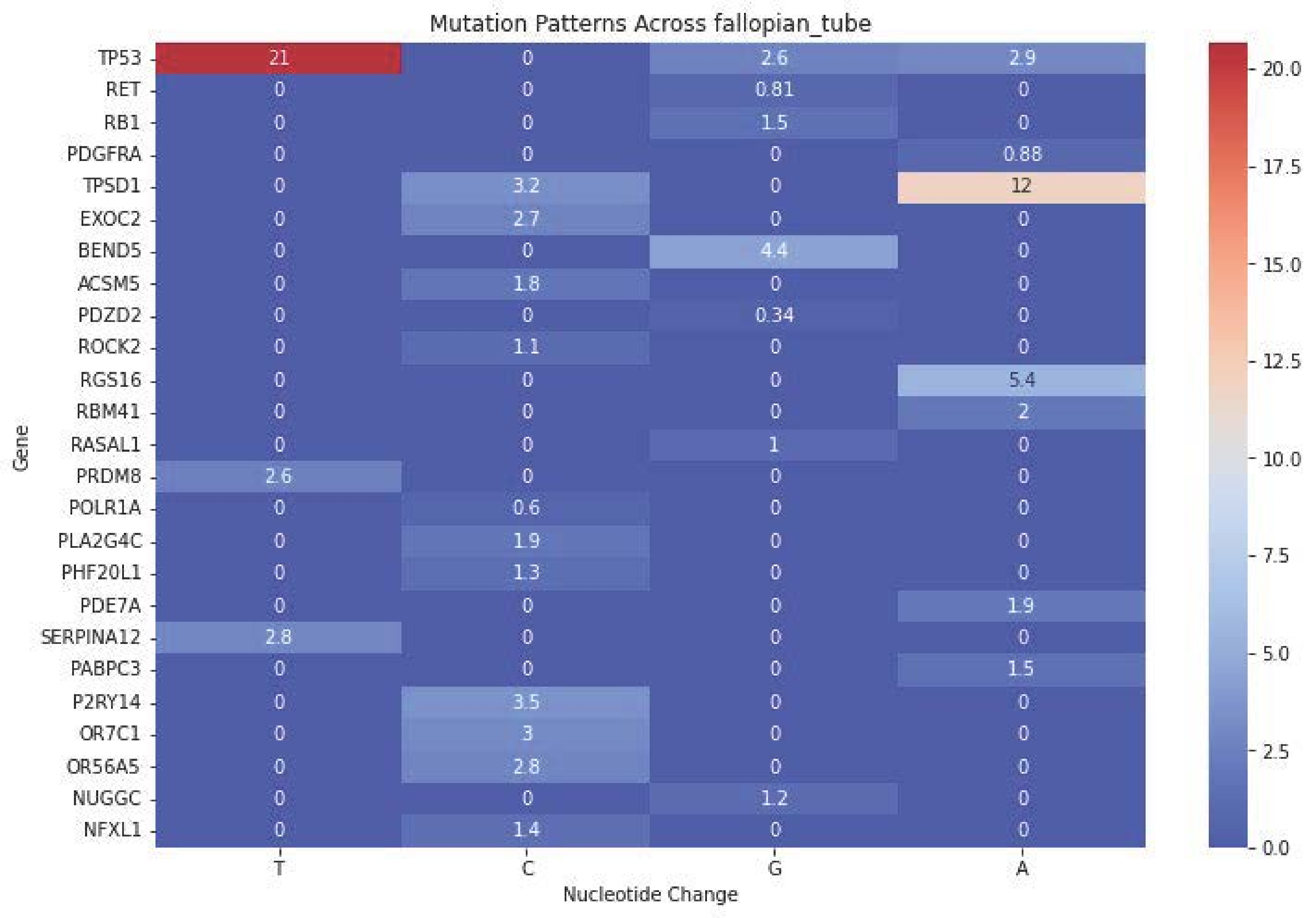
Mutation Patterns Across fallopian_tube
Gene
Nucleotide Change
T
C
G
A
TP53
RET
RB1
PDGFRA
TPSD1
EXOC2
BEND5
ACSM5
PDZD2
ROCK2
RGS16
RBM41
RASAL1
PRDM8
POLR1A
PLA2G4C
PHF20L1
PDE7A
SERPINA12
PABPC3
P2RY14
OR7C1
OR56A5
NUGGC
NFXL1
21
2.6
2.9
0.81
1.5
0.88
3.2
12
2.7
4.4
1.8
0.34
1.1
5.4
2
1
2.6
0.6
1.9
1.3
1.9
2.8
1.5
3.5
3
2.8
1.2
1.4
0.0
2.5
5.0
7.5
10.0
12.5
15.0
17.5
20.0

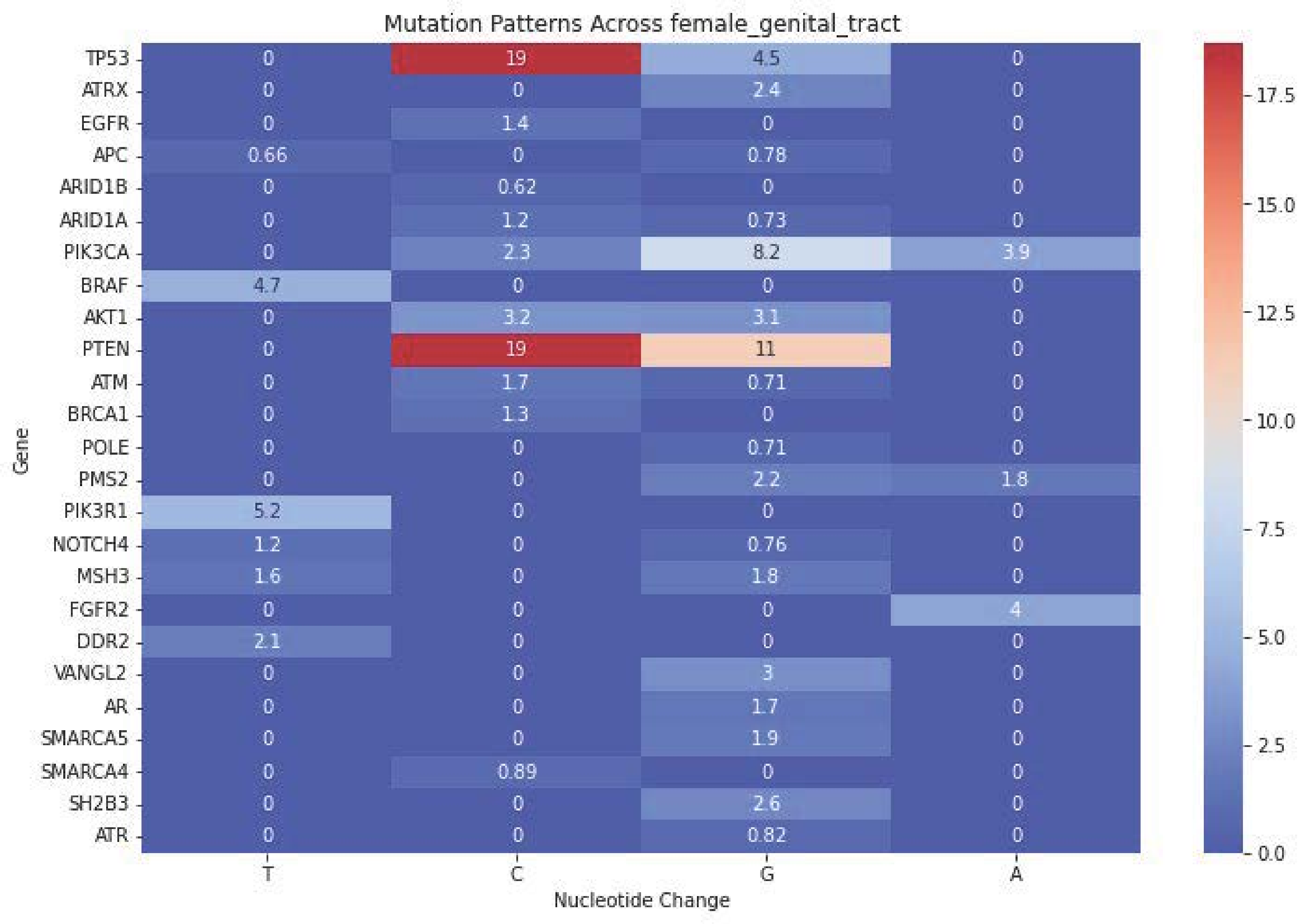

Mutation Patterns Across female_genital_tract
Gene
TP53
ATRX
EGFR
APC
ARID1B
ARID1A
PIK3CA
BRAF
AKT1
PTEN
ATM
BRCA1
POLE
PMS2
PIK3R1
NOTCH4
MSH3
FGFR2
DDR2
VANGL2
AR
SMARCA5
SMARCA4
SH2B3
ATR
Nucleotide Change
T
C
G
A
0 19 4.5 0
0 0 2.4 0
0 1.4 0 0
0.66 0 0.78 0
0 0.62 0 0
0 1.2 0.73 0
0 2.3 8.2 3.9
4.7 0 0 0
0 3.2 3.1 0
0 19 11 0
0 1.7 0.71 0
0 1.3 0 0
0 0 0.71 0
0 0 2.2 1.8
5.2 0 0 0
1.2 0 0.76 0
1.6 0 1.8 0
0 0 0 4
2.1 0 0 0
0 0 3 0
0 0 1.7 0
0 0 1.9 0
0 0.89 0 0
0 0 2.6 0
0 0 0.82 0
17.5
15.0
12.5
10.0
7.5
5.0
2.5
0.0

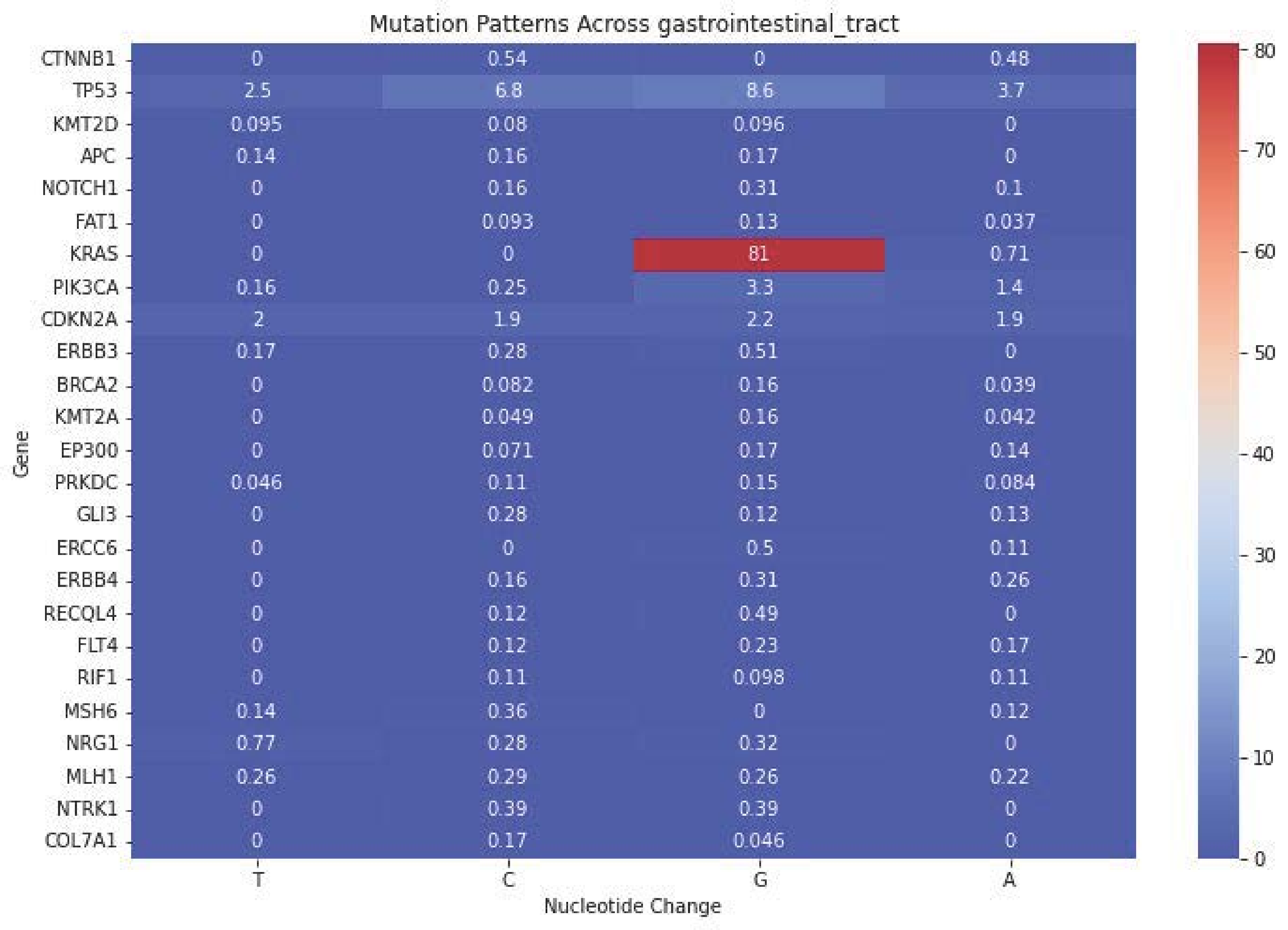
Mutation Patterns Across gastrointestinal_tract
Gene
Nucleotide Change
T
C
G
A
CTNNB1
TP53
KMT2D
APC
NOTCH1
FAT1
KRAS
PIK3CA
CDKN2A
ERBB3
BRCA2
KMT2A
EP300
PRKDC
GLI3
ERCC6
ERBB4
RECQL4
FLT4
RIF1
MSH6
NRG1
MLH1
NTRK1
COL7A1
0 0.54 0 0.48
2.5 6.8 8.6 3.7
0.095 0.08 0.096 0
0.14 0.16 0.17 0
0 0.16 0.31 0.1
0 0.093 0.13 0.037
0 0 81 0.71
0.16 0.25 3.3 1.4
2 1.9 2.2 1.9
0.17 0.28 0.51 0
0 0.082 0.16 0.039
0 0.049 0.16 0.042
0 0.071 0.17 0.14
0.046 0.11 0.15 0.084
0 0.28 0.12 0.13
0 0 0.5 0.11
0 0.16 0.31 0.26
0 0.12 0.49 0
0 0.12 0.23 0.17
0 0.11 0.098 0.11
0.14 0.36 0 0.12
0.77 0.28 0.32 0
0.26 0.29 0.26 0.22
0 0.39 0.39 0
0 0.17 0.046 0
80
70
60
50
40
30
20
10
0

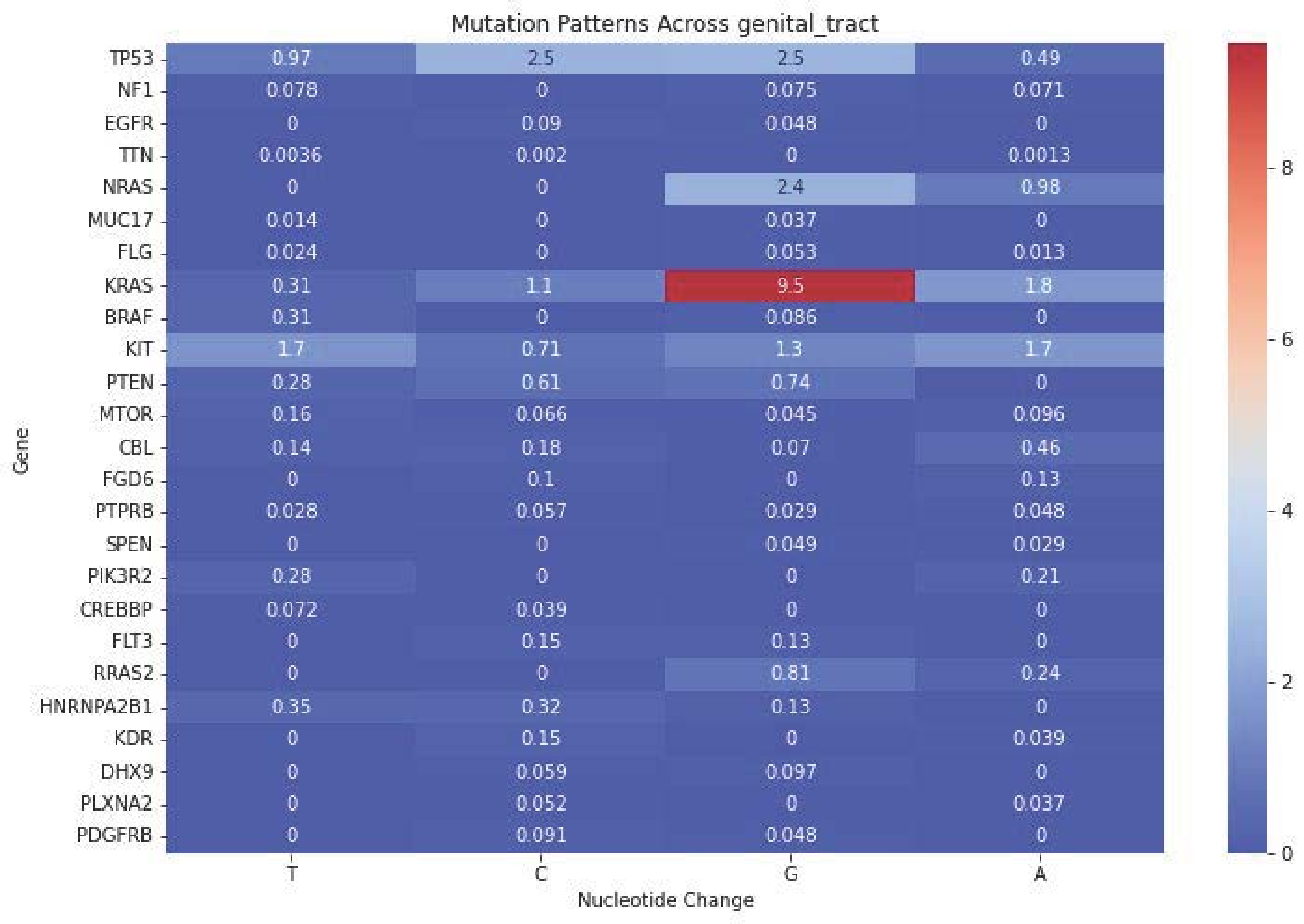
Mutation Patterns Across genital_tract
Gene
Nucleotide Change
T
C
G
A
TP53
NF1
EGFR
TTN
NRAS
MUC17
FLG
KRAS
BRAF
KIT
PTEN
MTOR
CBL
FGD6
PTPRB
SPEN
PIK3R2
CREBBP
FLT3
RRAS2
HNRNPA2B1
KDR
DHX9
PLXNA2
PDGFRB
0.97 2.5 2.5 0.49
0.078 0 0.075 0.071
0 0.09 0.048 0
0.0036 0.002 0 0.0013
0 0 2.4 0.98
0.014 0 0.037 0
0.024 0 0.053 0.013
0.31 1.1 9.5 1.8
0.31 0 0.086 0
1.7 0.71 1.3 1.7
0.28 0.61 0.74 0
0.16 0.066 0.045 0.096
0.14 0.18 0.07 0.46
0 0.1 0 0.13
0.028 0.057 0.029 0.048
0 0 0.049 0.029
0.28 0 0 0.21
0.072 0.039 0 0
0 0.15 0.13 0
0 0 0.81 0.24
0.35 0.32 0.13 0
0 0.15 0 0.039
0 0.059 0.097 0
0 0.052 0 0.037
0 0.091 0.048 0
8
6
4
2
0

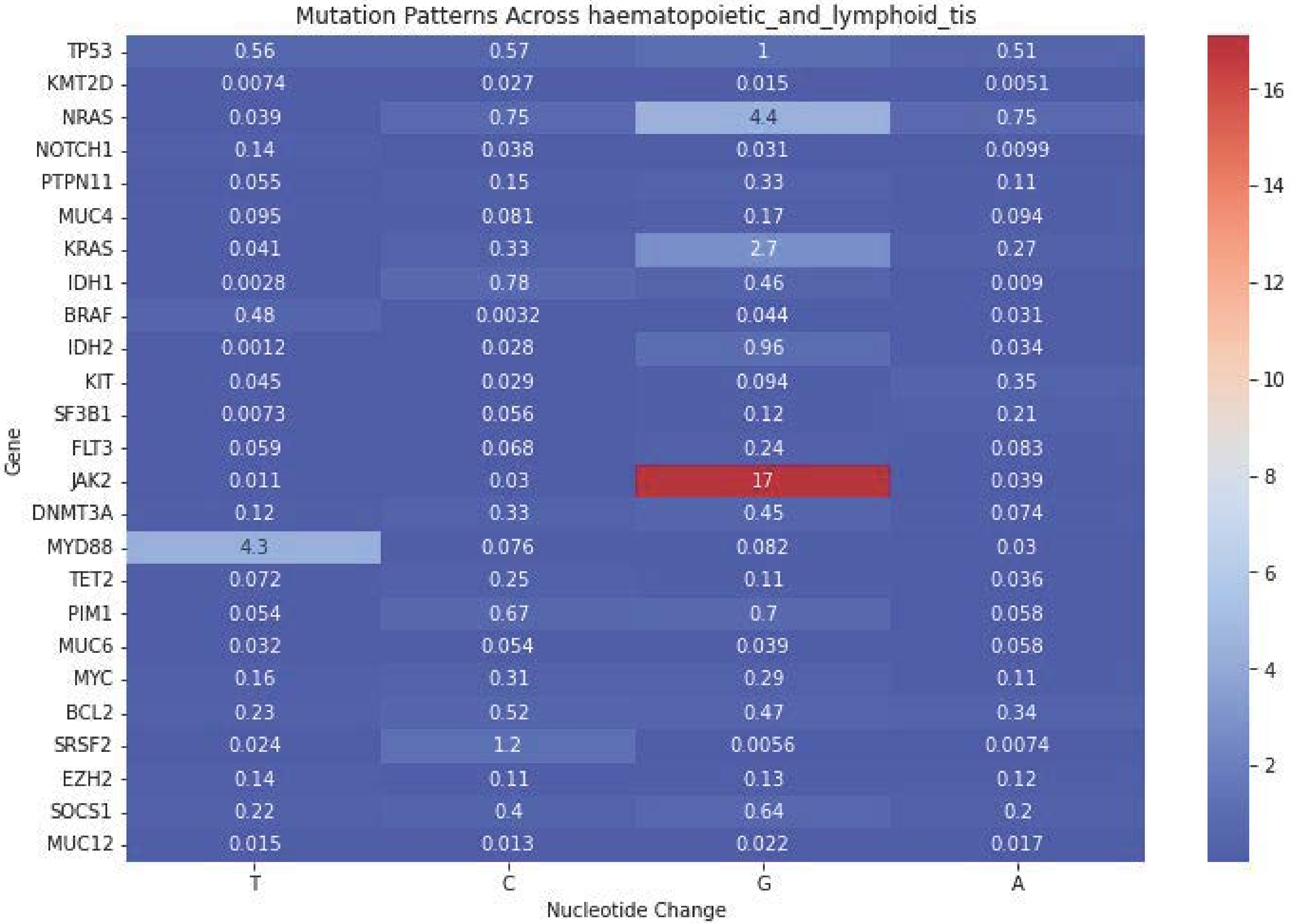

Mutation Patterns Across haematopoietic_and_lymphoid_tis
Gene
Nucleotide Change
T
C
G
A
TP53 0.56 0.57 1 0.51
KMT2D 0.0074 0.027 0.015 0.0051
NRAS 0.039 0.75 4.4 0.75
NOTCH1 0.14 0.038 0.031 0.0099
PTPN11 0.055 0.15 0.33 0.11
MUC4 0.095 0.081 0.17 0.094
KRAS 0.041 0.33 2.7 0.27
IDH1 0.0028 0.78 0.46 0.009
BRAF 0.48 0.0032 0.044 0.031
IDH2 0.0012 0.028 0.96 0.034
KIT 0.045 0.029 0.094 0.35
SF3B1 0.0073 0.056 0.12 0.21
FLT3 0.059 0.068 0.24 0.083
JAK2 0.011 0.03 17 0.039
DNMT3A 0.12 0.33 0.45 0.074
MYD88 4.3 0.076 0.082 0.03
TET2 0.072 0.25 0.11 0.036
PIM1 0.054 0.67 0.7 0.058
MUC6 0.032 0.054 0.039 0.058
MYC 0.16 0.31 0.29 0.11
BCL2 0.23 0.52 0.47 0.34
SRSF2 0.024 1.2 0.0056 0.0074
EZH2 0.14 0.11 0.13 0.12
SOCS1 0.22 0.4 0.64 0.2
MUC12 0.015 0.013 0.022 0.017
16
14
12
10
8
6
4
2

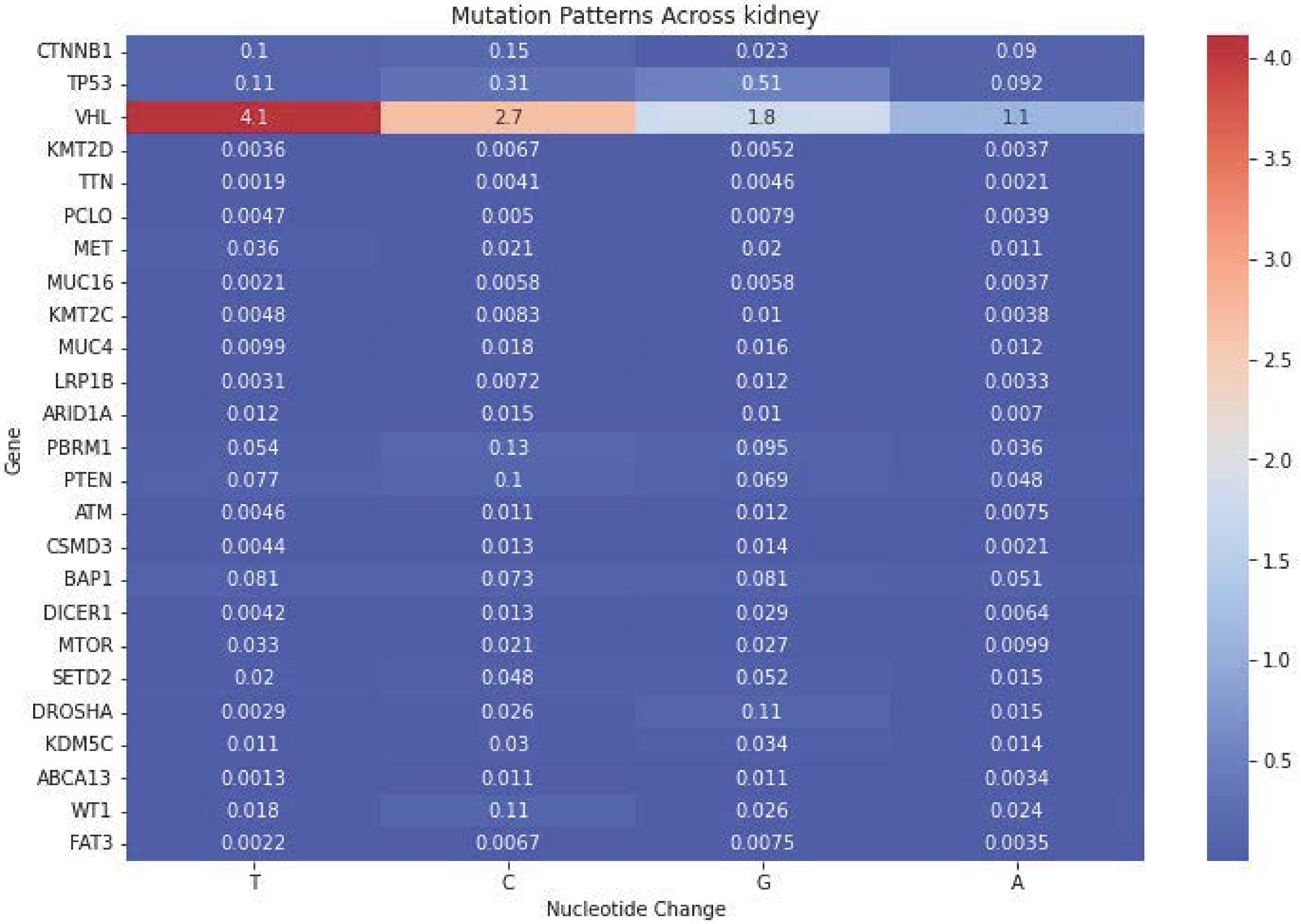
Mutation Patterns Across kidney
Gene
CTNNB1 0.1 0.15 0.023 0.09
TP53 0.11 0.31 0.51 0.092
VHL 4.1 2.7 1.8 1.1
KMT2D 0.0036 0.0067 0.0052 0.0037
TTN 0.0019 0.0041 0.0046 0.0021
PCLO 0.0047 0.005 0.0079 0.0039
MET 0.036 0.021 0.02 0.011
MUC16 0.0021 0.0058 0.0058 0.0037
KMT2C 0.0048 0.0083 0.01 0.0038
MUC4 0.0099 0.018 0.016 0.012
LRP1B 0.0031 0.0072 0.012 0.0033
ARID1A 0.012 0.015 0.01 0.007
PBRM1 0.054 0.13 0.095 0.036
PTEN 0.077 0.1 0.069 0.048
ATM 0.0046 0.011 0.012 0.0075
CSMD3 0.0044 0.013 0.014 0.0021
BAP1 0.081 0.073 0.081 0.051
DICER1 0.0042 0.013 0.029 0.0064
MTOR 0.033 0.021 0.027 0.0099
SETD2 0.02 0.048 0.052 0.015
DROSHA 0.0029 0.026 0.11 0.015
KDM5C 0.011 0.03 0.034 0.014
ABCA13 0.0013 0.011 0.011 0.0034
WT1 0.018 0.11 0.026 0.024
FAT3 0.0022 0.0067 0.0075 0.0035
T C G A
Nucleotide Change
4.0 3.5 3.0 2.5 2.0 1.5 1.0 0.5

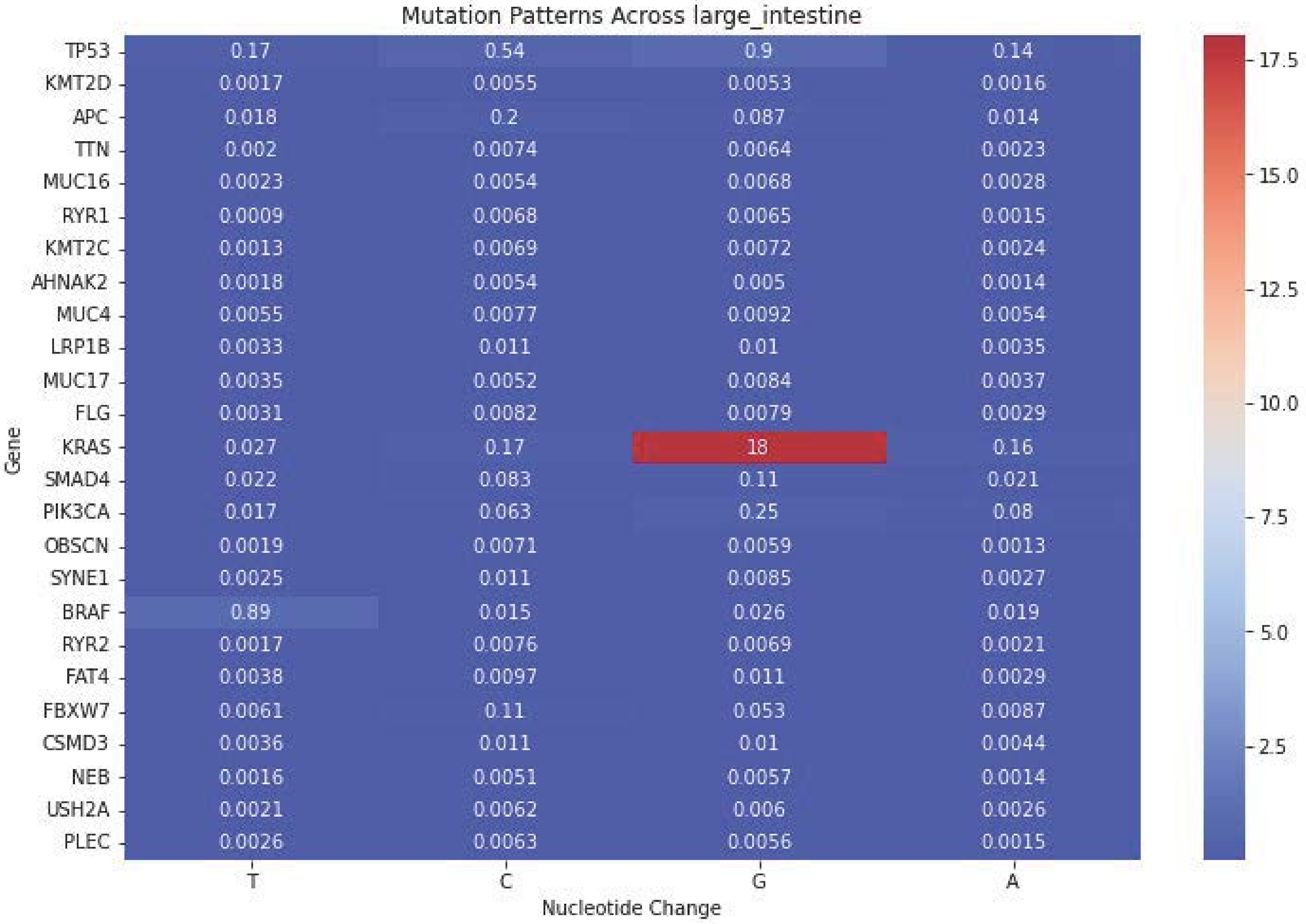
Mutation Patterns Across large_intestine
Gene
Nucleotide Change
T
C
G
A
TP53 0.17 0.54 0.9 0.14
KMT2D 0.0017 0.0055 0.0053 0.0016
APC 0.018 0.2 0.087 0.014
TTN 0.002 0.0074 0.0064 0.0023
MUC16 0.0023 0.0054 0.0068 0.0028
RYR1 0.0009 0.0068 0.0065 0.0015
KMT2C 0.0013 0.0069 0.0072 0.0024
AHNAK2 0.0018 0.0054 0.005 0.0014
MUC4 0.0055 0.0077 0.0092 0.0054
LRP1B 0.0033 0.011 0.01 0.0035
MUC17 0.0035 0.0052 0.0084 0.0037
FLG 0.0031 0.0082 0.0079 0.0029
KRAS 0.027 0.17 18 0.16
SMAD4 0.022 0.083 0.11 0.021
PIK3CA 0.017 0.063 0.25 0.08
OBSCN 0.0019 0.0071 0.0059 0.0013
SYNE1 0.0025 0.011 0.0085 0.0027
BRAF 0.89 0.015 0.026 0.019
RYR2 0.0017 0.0076 0.0069 0.0021
FAT4 0.0038 0.0097 0.011 0.0029
FBXW7 0.0061 0.11 0.053 0.0087
CSMD3 0.0036 0.011 0.01 0.0044
NEB 0.0016 0.0051 0.0057 0.0014
USH2A 0.0021 0.0062 0.006 0.0026
PLEC 0.0026 0.0063 0.0056 0.0015
17.5
15.0
12.5
10.0
7.5
5.0
2.5

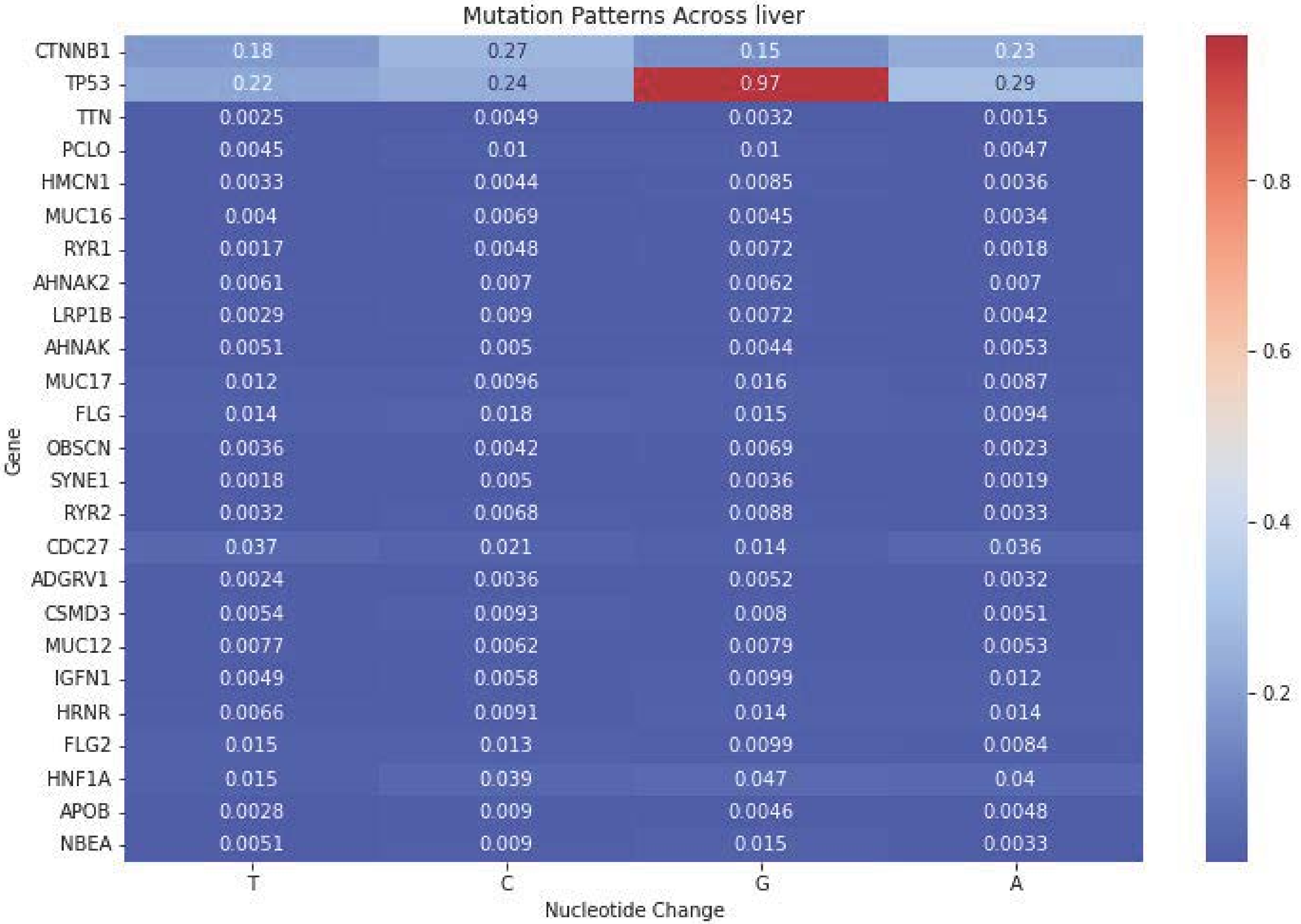
Mutation Patterns Across liver
Gene
Nucleotide Change
T
C
G
A
CTNNB1 0.18 0.27 0.15 0.23
TP53 0.22 0.24 0.97 0.29
TTN 0.0025 0.0049 0.0032 0.0015
PCLO 0.0045 0.01 0.01 0.0047
HMCN1 0.0033 0.0044 0.0085 0.0036
MUC16 0.004 0.0069 0.0045 0.0034
RYR1 0.0017 0.0048 0.0072 0.0018
AHNAK2 0.0061 0.007 0.0062 0.007
LRP1B 0.0029 0.009 0.0072 0.0042
AHNAK 0.0051 0.005 0.0044 0.0053
MUC17 0.012 0.0096 0.016 0.0087
FLG 0.014 0.018 0.015 0.0094
OBSCN 0.0036 0.0042 0.0069 0.0023
SYNE1 0.0018 0.005 0.0036 0.0019
RYR2 0.0032 0.0068 0.0088 0.0033
CDC27 0.037 0.021 0.014 0.036
ADGRV1 0.0024 0.0036 0.0052 0.0032
CSMD3 0.0054 0.0093 0.008 0.0051
MUC12 0.0077 0.0062 0.0079 0.0053
IGFN1 0.0049 0.0058 0.0099 0.012
HRNR 0.0066 0.0091 0.014 0.014
FLG2 0.015 0.013 0.0099 0.0084
HNF1A 0.015 0.039 0.047 0.04
APOB 0.0028 0.009 0.0046 0.0048
NBEA 0.0051 0.009 0.015 0.0033
0.8
0.6
0.4
0.2

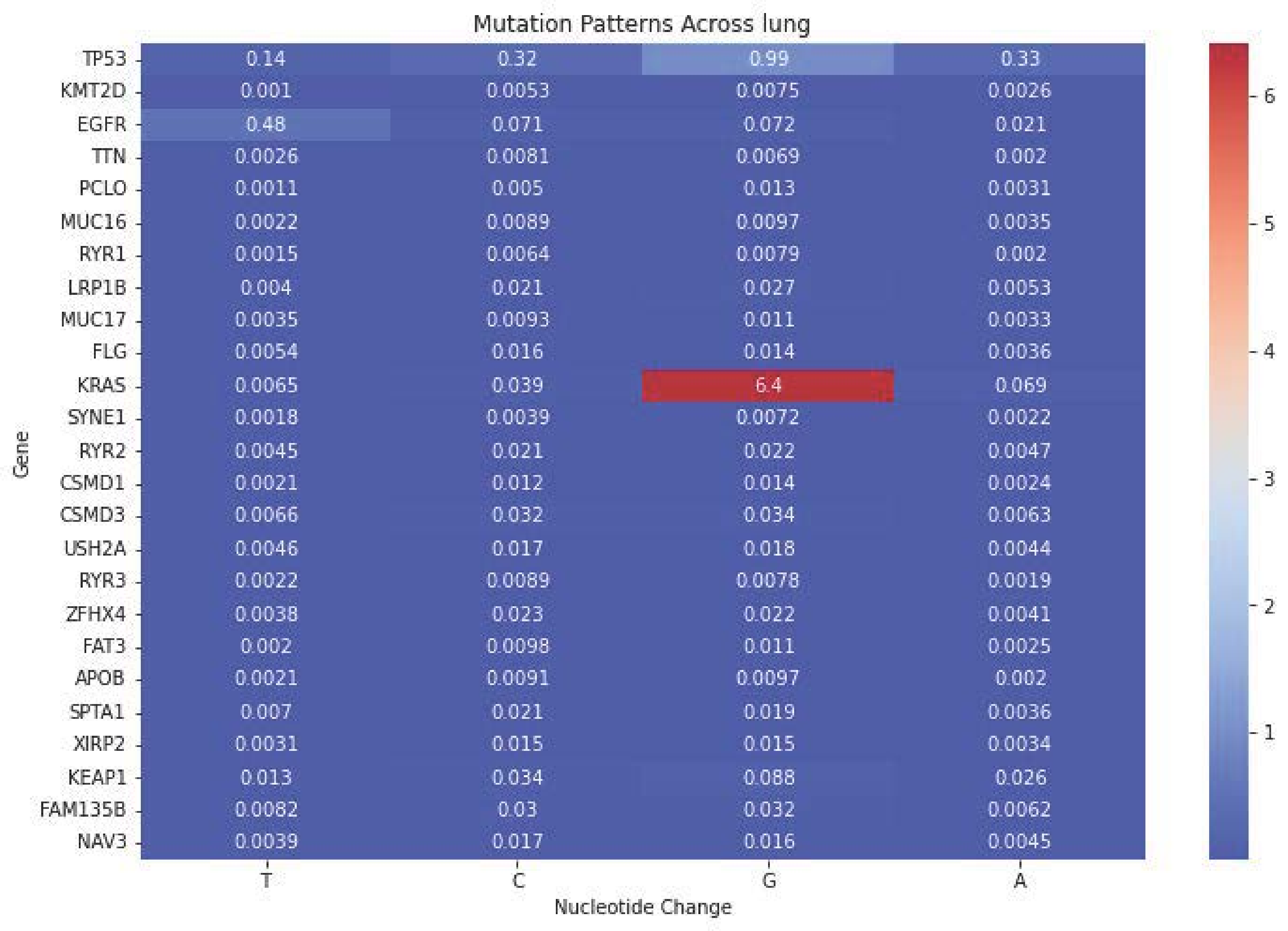

Mutation Patterns Across lung
Gene
Nucleotide Change
T
C
G
A
TP53 0.14 0.32 0.99 0.33
KMT2D 0.001 0.0053 0.0075 0.0026
EGFR 0.48 0.071 0.072 0.021
TTN 0.0026 0.0081 0.0069 0.002
PCLO 0.0011 0.005 0.013 0.0031
MUC16 0.0022 0.0089 0.0097 0.0035
RYR1 0.0015 0.0064 0.0079 0.002
LRP1B 0.004 0.021 0.027 0.0053
MUC17 0.0035 0.0093 0.011 0.0033
FLG 0.0054 0.016 0.014 0.0036
KRAS 0.0065 0.039 6.4 0.069
SYNE1 0.0018 0.0039 0.0072 0.0022
RYR2 0.0045 0.021 0.022 0.0047
CSMD1 0.0021 0.012 0.014 0.0024
CSMD3 0.0066 0.032 0.034 0.0063
USH2A 0.0046 0.017 0.018 0.0044
RYR3 0.0022 0.0089 0.0078 0.0019
ZFHX4 0.0038 0.023 0.022 0.0041
FAT3 0.002 0.0098 0.011 0.0025
APOB 0.0021 0.0091 0.0097 0.002
SPTA1 0.007 0.021 0.019 0.0036
XIRP2 0.0031 0.015 0.015 0.0034
KEAP1 0.013 0.034 0.088 0.026
FAM135B 0.0082 0.03 0.032 0.0062
NAV3 0.0039 0.017 0.016 0.0045
1
2
3
4
5
6

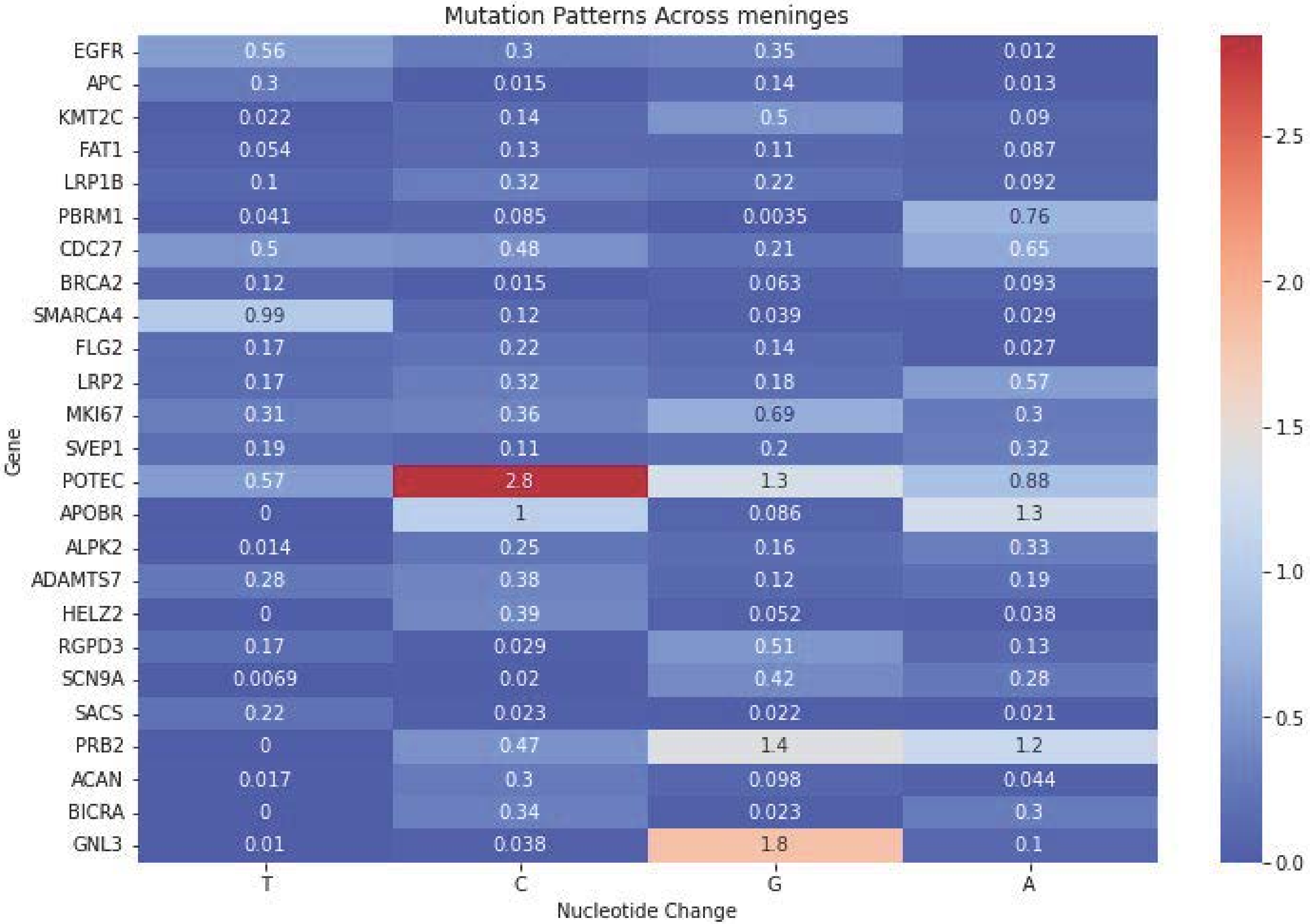

Mutation Patterns Across meninges
EGFR
APC
KMT2C
FAT1
LRP1B
PBRM1
CDC27
BRCA2
SMARCA4
FLG2
LRP2
MKI67
SVEP1
POTEC
APOBR
ALPK2
ADAMTS7
HELZ2
RGPD3
SCN9A
SACS
PRB2
ACAN
BICRA
GNL3
Gene
T
C
G
A
Nucleotide Change
0.0
0.5
1.0
1.5
2.0
2.5

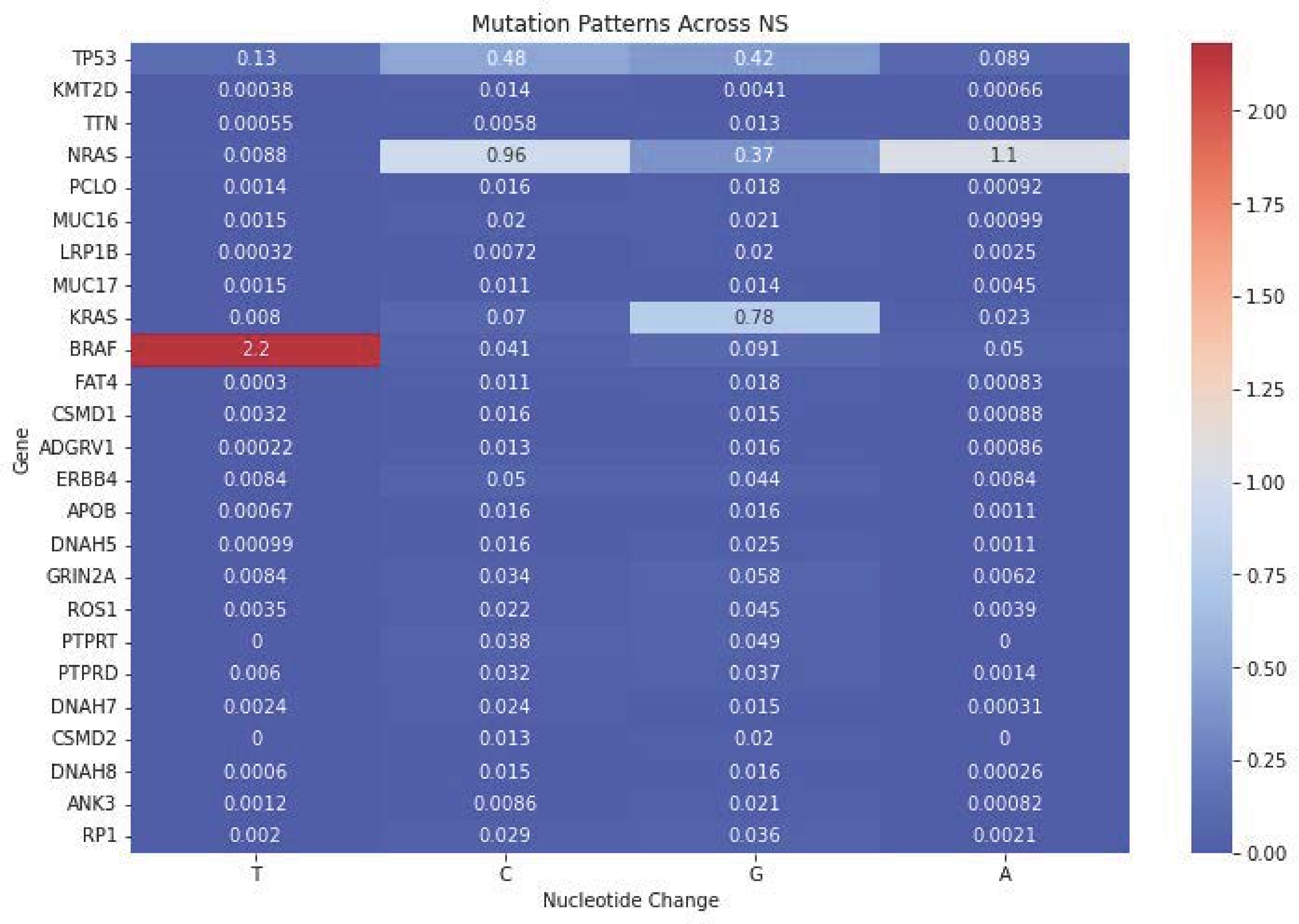
Mutation Patterns Across NS
Gene
Nucleotide Change
T
C
G
A
TP53 0.13 0.48 0.42 0.089
KMT2D 0.00038 0.014 0.0041 0.00066
TTN 0.00055 0.0058 0.013 0.00083
NRAS 0.0088 0.96 0.37 1.1
PCLO 0.0014 0.016 0.018 0.00092
MUC16 0.0015 0.02 0.021 0.00099
LRP1B 0.00032 0.0072 0.02 0.0025
MUC17 0.0015 0.011 0.014 0.0045
KRAS 0.008 0.07 0.78 0.023
BRAF 2.2 0.041 0.091 0.05
FAT4 0.0003 0.011 0.018 0.00083
CSMD1 0.0032 0.016 0.015 0.00088
ADGRV1 0.00022 0.013 0.016 0.00086
ERBB4 0.0084 0.05 0.044 0.0084
APOB 0.00067 0.016 0.016 0.0011
DNAH5 0.00099 0.016 0.025 0.0011
GRIN2A 0.0084 0.034 0.058 0.0062
ROS1 0.0035 0.022 0.045 0.0039
PTPRT 0 0.038 0.049 0
PTPRD 0.006 0.032 0.037 0.0014
DNAH7 0.0024 0.024 0.015 0.00031
CSMD2 0 0.013 0.02 0
DNAH8 0.0006 0.015 0.016 0.00026
ANK3 0.0012 0.0086 0.021 0.00082
RP1 0.002 0.029 0.036 0.0021
2.00
1.75
1.50
1.25
1.00
0.75
0.50
0.25
0.00

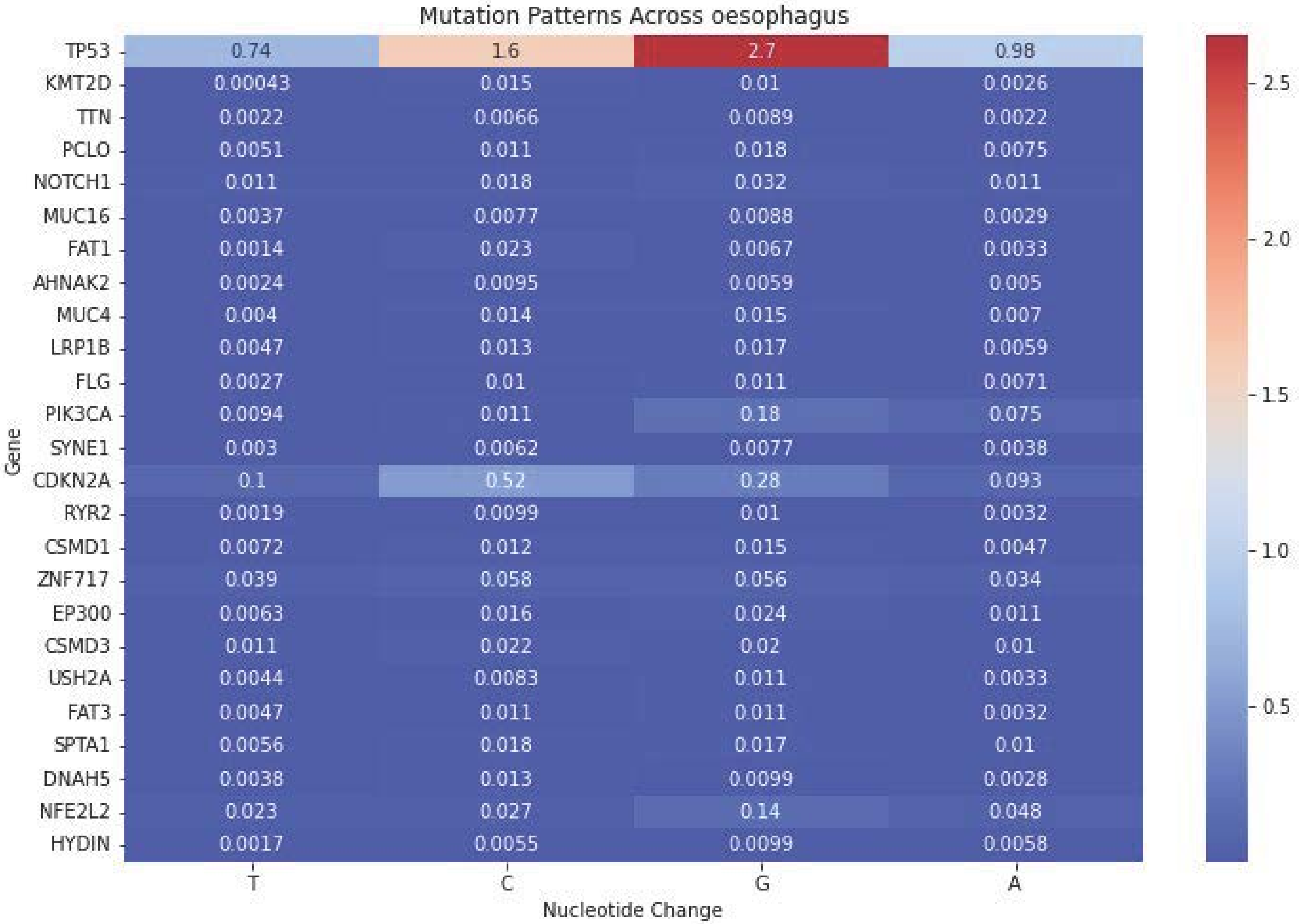
Mutation Patterns Across oesophagus
Gene
TP53 0.74 1.6 2.7 0.98
KMT2D 0.00043 0.015 0.01 0.0026
TTN 0.0022 0.0066 0.0089 0.0022
PCLO 0.0051 0.011 0.018 0.0075
NOTCH1 0.011 0.018 0.032 0.011
MUC16 0.0037 0.0077 0.0088 0.0029
FAT1 0.0014 0.023 0.0067 0.0033
AHNAK2 0.0024 0.0095 0.0059 0.005
MUC4 0.004 0.014 0.015 0.007
LRP1B 0.0047 0.013 0.017 0.0059
FLG 0.0027 0.01 0.011 0.0071
PIK3CA 0.0094 0.011 0.18 0.075
SYNE1 0.003 0.0062 0.0077 0.0038
CDKN2A 0.1 0.52 0.28 0.093
RYR2 0.0019 0.0099 0.01 0.0032
CSMD1 0.0072 0.012 0.015 0.0047
ZNF717 0.039 0.058 0.056 0.034
EP300 0.0063 0.016 0.024 0.011
CSMD3 0.011 0.022 0.02 0.01
USH2A 0.0044 0.0083 0.011 0.0033
FAT3 0.0047 0.011 0.011 0.0032
SPTA1 0.0056 0.018 0.017 0.01
DNAH5 0.0038 0.013 0.0099 0.0028
NFE2L2 0.023 0.027 0.14 0.048
HYDIN 0.0017 0.0055 0.0099 0.0058
T C G A
Nucleotide Change
2.5
2.0
1.5
1.0
0.5

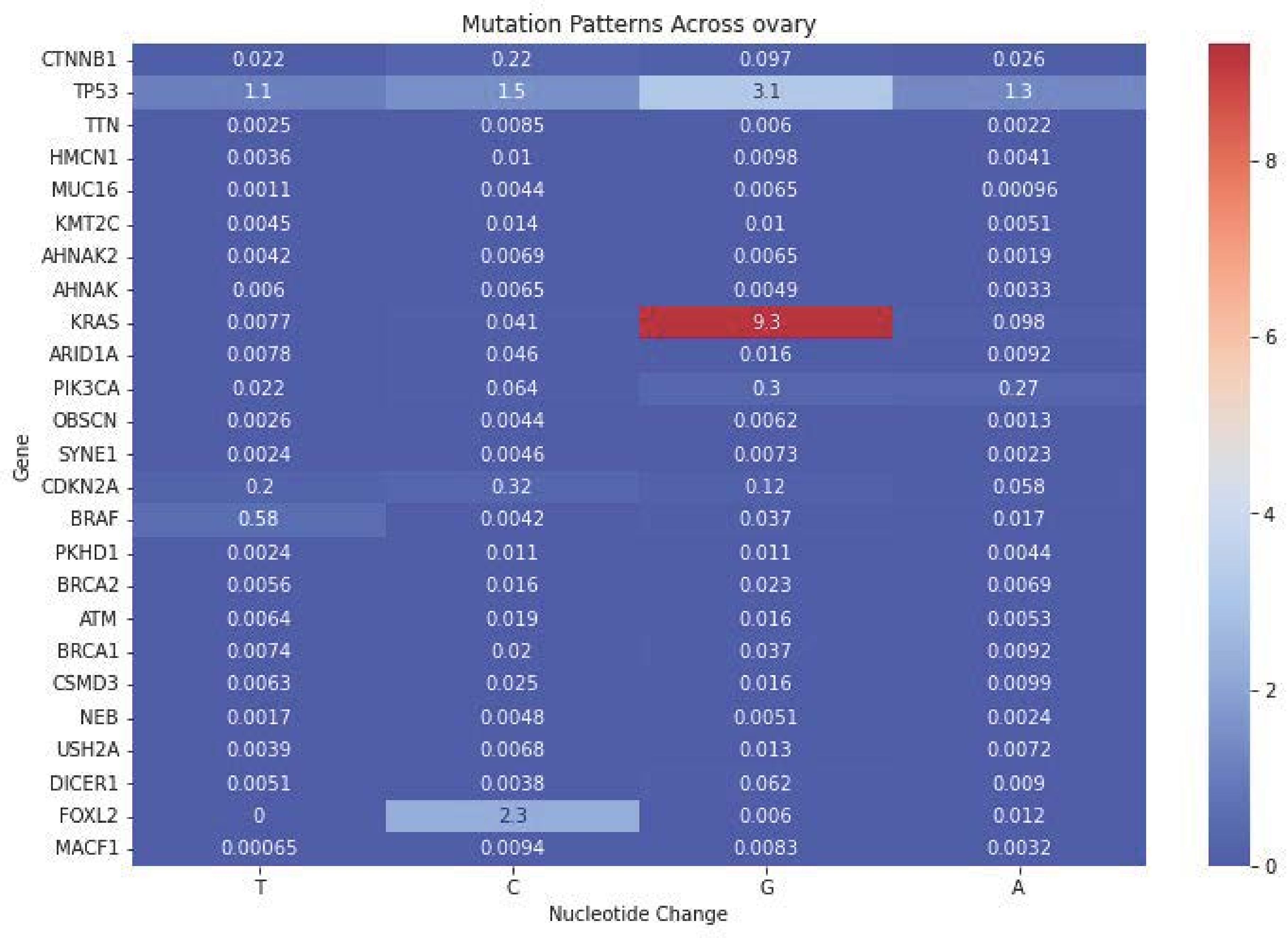

Mutation Patterns Across ovary
Gene
Nucleotide Change
T
C
G
A
CTNNB1 0.022 0.22 0.097 0.026
TP53 1.1 1.5 3.1 1.3
TTN 0.0025 0.0085 0.006 0.0022
HMCN1 0.0036 0.01 0.0098 0.0041
MUC16 0.0011 0.0044 0.0065 0.00096
KMT2C 0.0045 0.014 0.01 0.0051
AHNAK2 0.0042 0.0069 0.0065 0.0019
AHNAK 0.006 0.0065 0.0049 0.0033
KRAS 0.0077 0.041 9.3 0.098
ARID1A 0.0078 0.046 0.016 0.0092
PIK3CA 0.022 0.064 0.3 0.27
OBSCN 0.0026 0.0044 0.0062 0.0013
SYNE1 0.0024 0.0046 0.0073 0.0023
CDKN2A 0.2 0.32 0.12 0.058
BRAF 0.58 0.0042 0.037 0.017
PKHD1 0.0024 0.011 0.011 0.0044
BRCA2 0.0056 0.016 0.023 0.0069
ATM 0.0064 0.019 0.016 0.0053
BRCA1 0.0074 0.02 0.037 0.0092
CSMD3 0.0063 0.025 0.016 0.0099
NEB 0.0017 0.0048 0.0051 0.0024
USH2A 0.0039 0.0068 0.013 0.0072
DICER1 0.0051 0.0038 0.062 0.009
FOXL2 0 2.3 0.006 0.012
MACF1 0.00065 0.0094 0.0083 0.0032
0
2
4
6
8

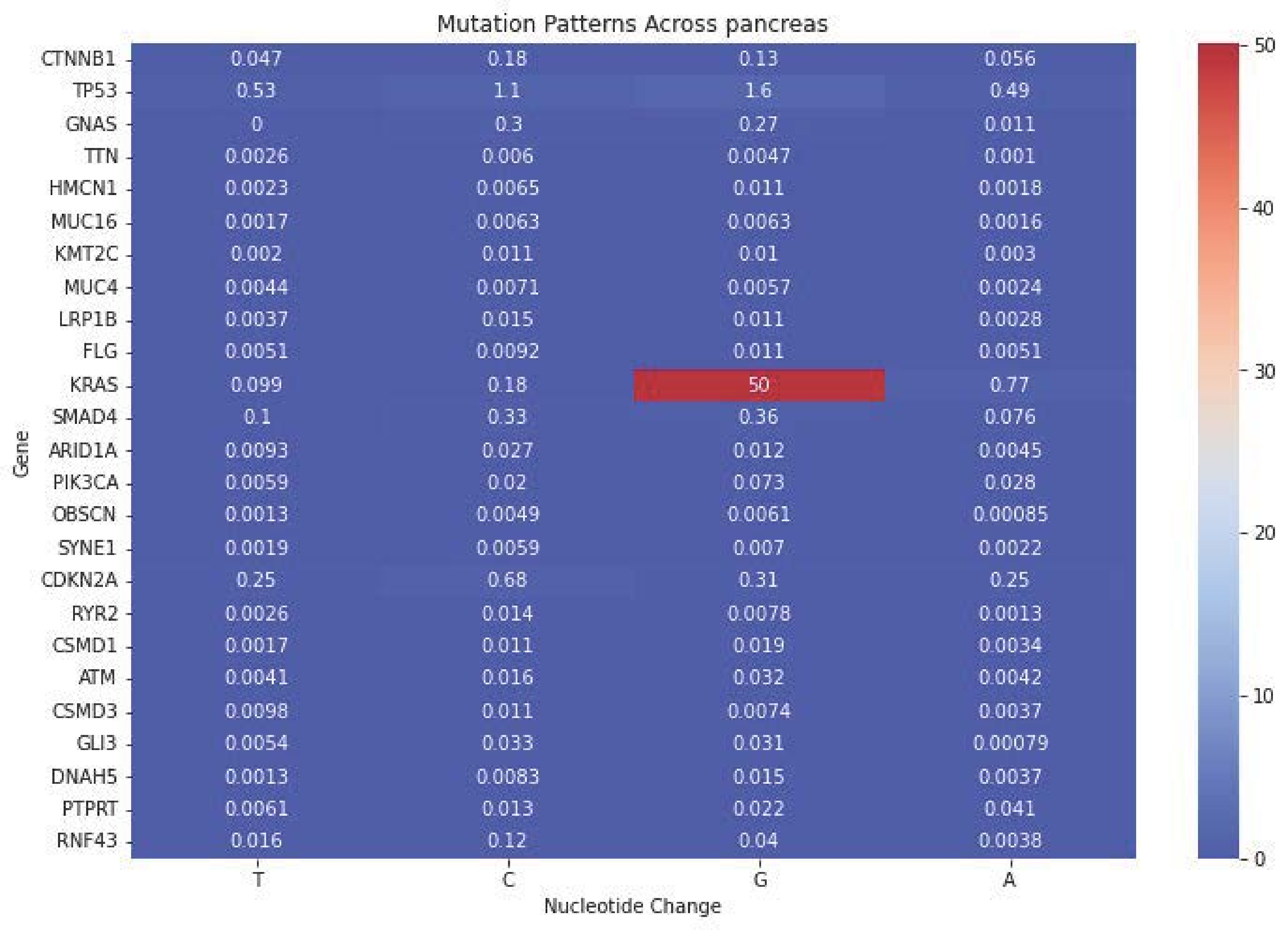

Mutation Patterns Across pancreas
Gene
Nucleotide Change
T
C
G
A
CTNNB1 0.047 0.18 0.13 0.056
TP53 0.53 1.1 1.6 0.49
GNAS 0 0.3 0.27 0.011
TTN 0.0026 0.006 0.0047 0.001
HMCN1 0.0023 0.0065 0.011 0.0018
MUC16 0.0017 0.0063 0.0063 0.0016
KMT2C 0.002 0.011 0.01 0.003
MUC4 0.0044 0.0071 0.0057 0.0024
LRP1B 0.0037 0.015 0.011 0.0028
FLG 0.0051 0.0092 0.011 0.0051
KRAS 0.099 0.18 50 0.77
SMAD4 0.1 0.33 0.36 0.076
ARID1A 0.0093 0.027 0.012 0.0045
PIK3CA 0.0059 0.02 0.073 0.028
OBSCN 0.0013 0.0049 0.0061 0.00085
SYNE1 0.0019 0.0059 0.007 0.0022
CDKN2A 0.25 0.68 0.31 0.25
RYR2 0.0026 0.014 0.0078 0.0013
CSMD1 0.0017 0.011 0.019 0.0034
ATM 0.0041 0.016 0.032 0.0042
CSMD3 0.0098 0.011 0.0074 0.0037
GLI3 0.0054 0.033 0.031 0.00079
DNAH5 0.0013 0.0083 0.015 0.0037
PTPRT 0.0061 0.013 0.022 0.041
RNF43 0.016 0.12 0.04 0.0038
50
40
30
20
10
0

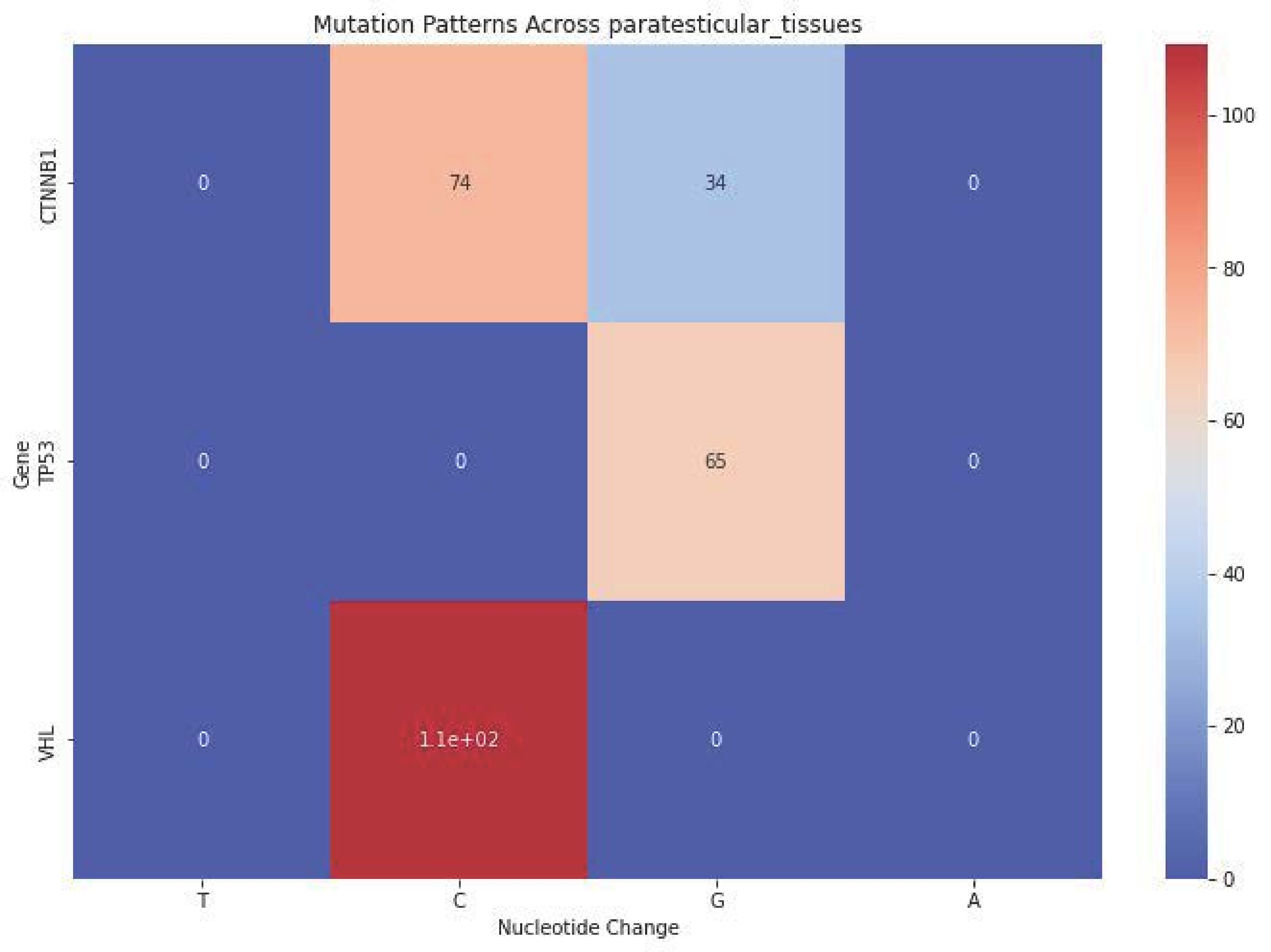
Mutation Patterns Across paratesticular_tissues
CTNNB1
TP53
VHL
Gene
0
74
34
0
0
0
65
0
0
1.1e+02
0
0
T
C
G
A
Nucleotide Change
100
80
60
40
20
0

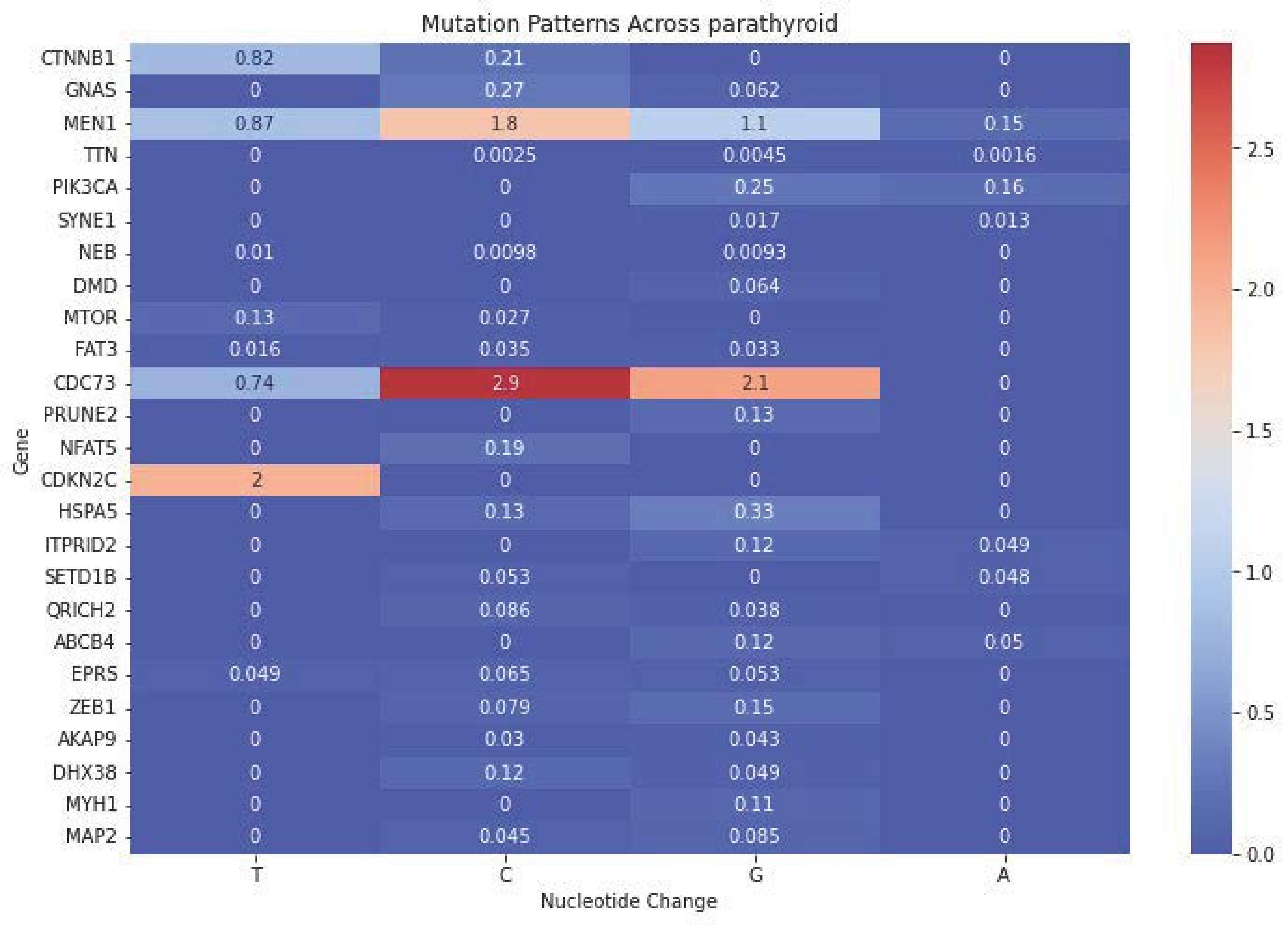

Mutation Patterns Across parathyroid
Gene
CTNNB1
GNAS
MEN1
TTN
PIK3CA
SYNE1
NEB
DMD
MTOR
FAT3
CDC73
PRUNE2
NFAT5
CDKN2C
HSPA5
ITPRID2
SETD1B
QRICH2
ABCB4
EPRS
ZEB1
AKAP9
DHX38
MYH1
MAP2
T
C
G
A
Nucleotide Change
0.82 0.21 0 0
0 0.27 0.062 0
0.87 1.8 1.1 0.15
0 0.0025 0.0045 0.0016
0 0 0.25 0.16
0 0 0.017 0.013
0.01 0.0098 0.0093 0
0 0 0.064 0
0.13 0.027 0 0
0.016 0.035 0.033 0
0.74 2.9 2.1 0
0 0 0.13 0
0 0.19 0 0
2 0 0 0
0 0.13 0.33 0
0 0 0.12 0.049
0 0.053 0 0.048
0 0.086 0.038 0
0 0 0.12 0.05
0.049 0.065 0.053 0
0 0.079 0.15 0
0 0.03 0.043 0
0 0.12 0.049 0
0 0 0.11 0
0 0.045 0.085 0
2.5
2.0
1.5
1.0
0.5
0.0

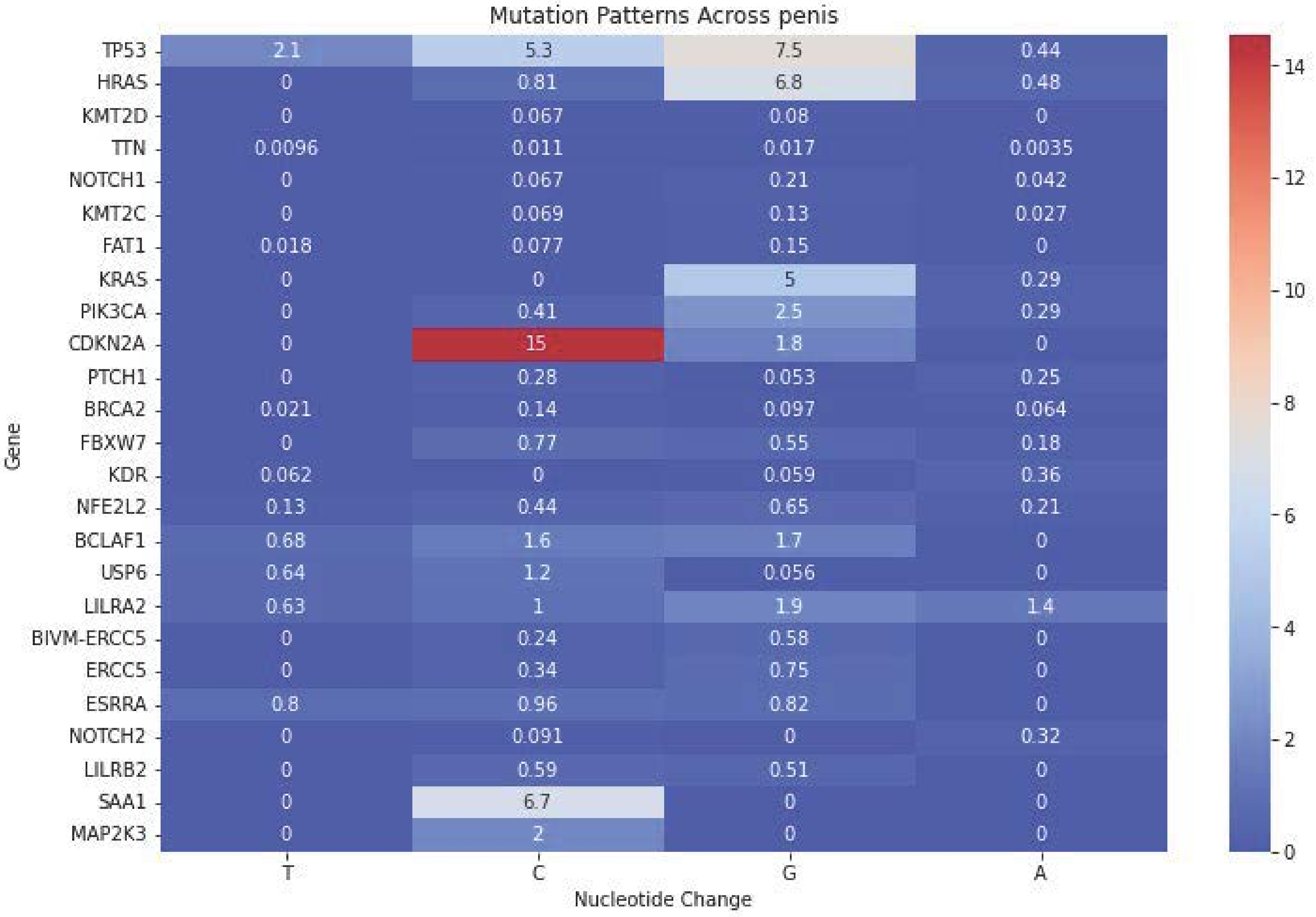
Mutation Patterns Across penis
Gene
Nucleotide Change
T
C
G
A
TP53
HRAS
KMT2D
TTN
NOTCH1
KMT2C
FAT1
KRAS
PIK3CA
CDKN2A
PTCH1
BRCA2
FBXW7
KDR
NFE2L2
BCLAF1
USP6
LILRA2
BIVM-ERCC5
ERCC5
ESRRA
NOTCH2
LILRB2
SAA1
MAP2K3
2.1 5.3 7.5 0.44
0 0.81 6.8 0.48
0 0.067 0.08 0
0.0096 0.011 0.017 0.0035
0 0.067 0.21 0.042
0 0.069 0.13 0.027
0.018 0.077 0.15 0
0 0 5 0.29
0 0.41 2.5 0.29
0 15 1.8 0
0 0.28 0.053 0.25
0.021 0.14 0.097 0.064
0 0.77 0.55 0.18
0.062 0 0.059 0.36
0.13 0.44 0.65 0.21
0.68 1.6 1.7 0
0.64 1.2 0.056 0
0.63 1 1.9 1.4
0 0.24 0.58 0
0 0.34 0.75 0
0.8 0.96 0.82 0
0 0.091 0 0.32
0 0.59 0.51 0
0 6.7 0 0
0 2 0 0
0
2
4
6
8
10
12
14

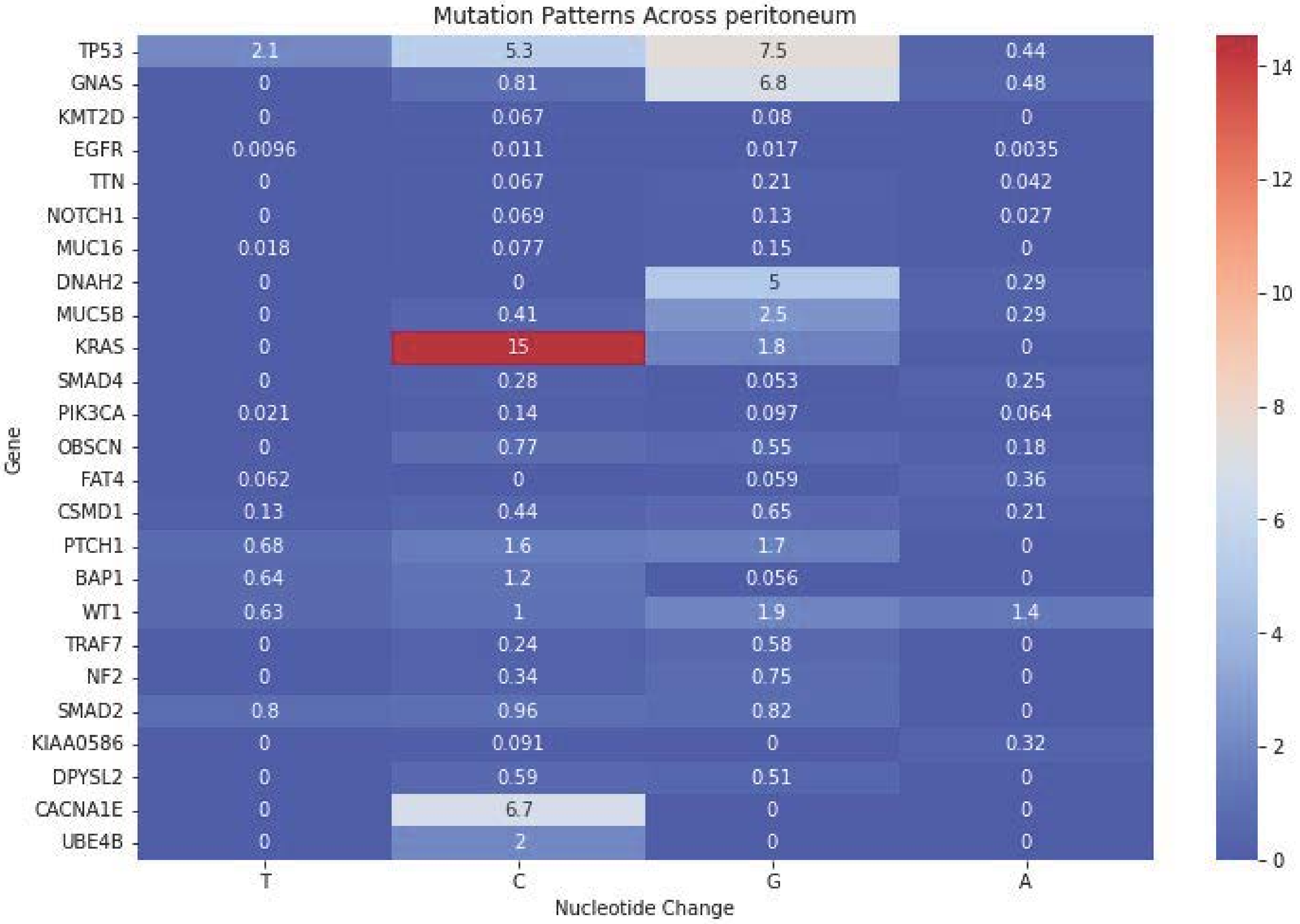

Mutation Patterns Across peritoneum
Gene
Nucleotide Change
T
C
G
A
TP53
GNAS
KMT2D
EGFR
TTN
NOTCH1
MUC16
DNAH2
MUC5B
KRAS
SMAD4
PIK3CA
OBSCN
FAT4
CSMD1
PTCH1
BAP1
WT1
TRAF7
NF2
SMAD2
KIAA0586
DPYSL2
CACNA1E
UBE4B
2.1
5.3
7.5
0.44
0
0.81
6.8
0.48
0
0.067
0.08
0
0.0096
0.011
0.017
0.0035
0
0.067
0.21
0.042
0
0.069
0.13
0.027
0.018
0.077
0.15
0
0
0
5
0.29
0
0.41
2.5
0.29
0
15
1.8
0
0
0.28
0.053
0.25
0.021
0.14
0.097
0.064
0
0.77
0.55
0.18
0.062
0
0.059
0.36
0.13
0.44
0.65
0.21
0.68
1.6
1.7
0
0.64
1.2
0.056
0
0.63
1
1.9
1.4
0
0.24
0.58
0
0
0.34
0.75
0
0.8
0.96
0.82
0
0
0.091
0
0.32
0
0.59
0.51
0
0
6.7
0
0
0
2
0
0
14
12
10
8
6
4
2
0

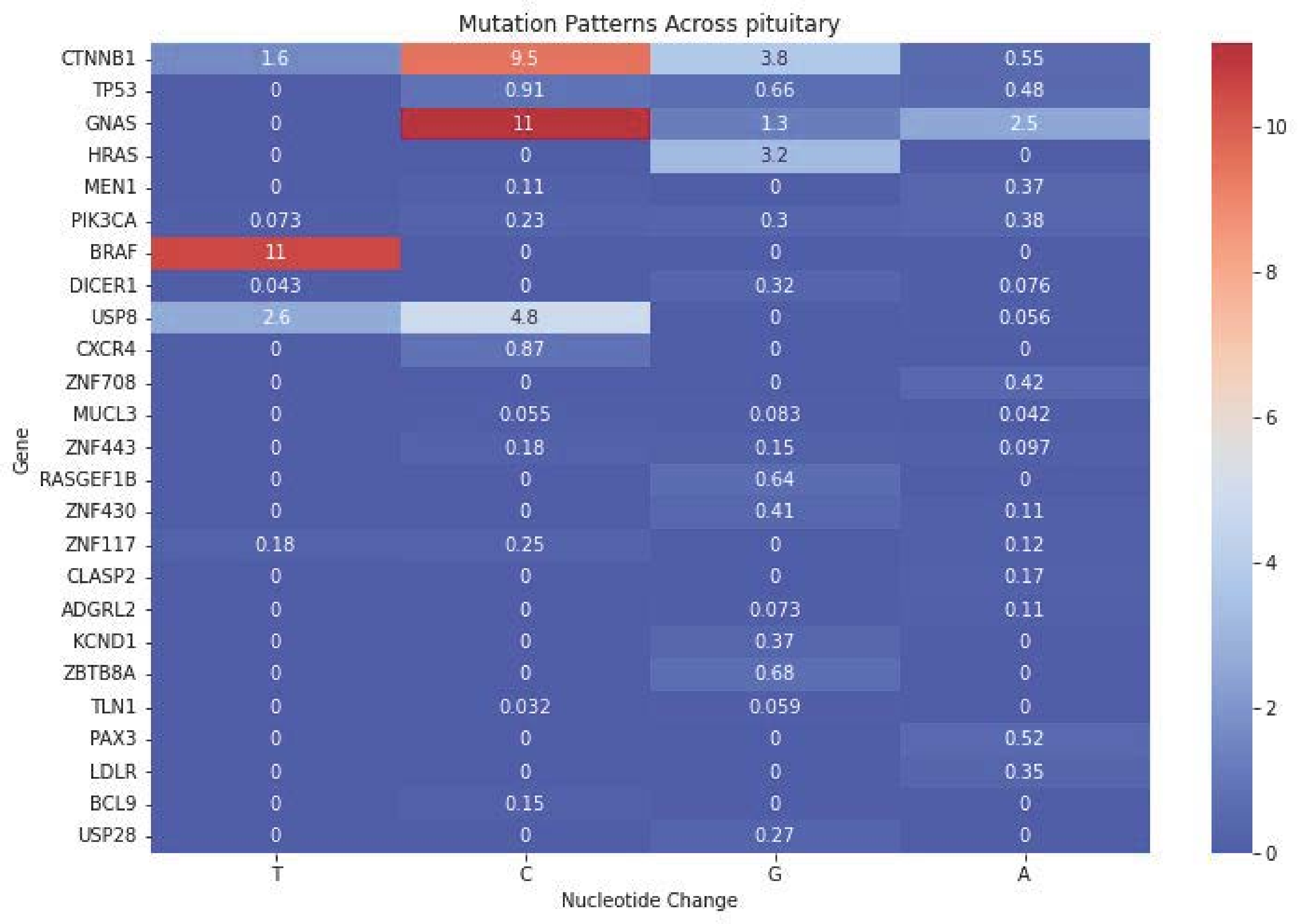

Mutation Patterns Across pituitary
Gene
Nucleotide Change
T
C
G
A
CTNNB1
TP53
GNAS
HRAS
MEN1
PIK3CA
BRAF
DICER1
USP8
CXCR4
ZNF708
MUCL3
ZNF443
RASGEF1B
ZNF430
ZNF117
CLASP2
ADGRL2
KCND1
ZBTB8A
TLN1
PAX3
LDLR
BCL9
USP28
1.6 9.5 3.8 0.55
0 0.91 0.66 0.48
0 11 1.3 2.5
0 0 3.2 0
0 0.11 0 0.37
0.073 0.23 0.3 0.38
11 0 0 0
0.043 0 0.32 0.076
2.6 4.8 0 0.056
0 0.87 0 0
0 0 0 0.42
0 0.055 0.083 0.042
0 0.18 0.15 0.097
0 0 0.64 0
0 0 0.41 0.11
0.18 0.25 0 0.12
0 0 0 0.17
0 0 0.073 0.11
0 0 0.37 0
0 0 0.68 0
0 0.032 0.059 0
0 0 0 0.52
0 0 0 0.35
0 0.15 0 0
0 0 0.27 0
0
2
4
6
8
10

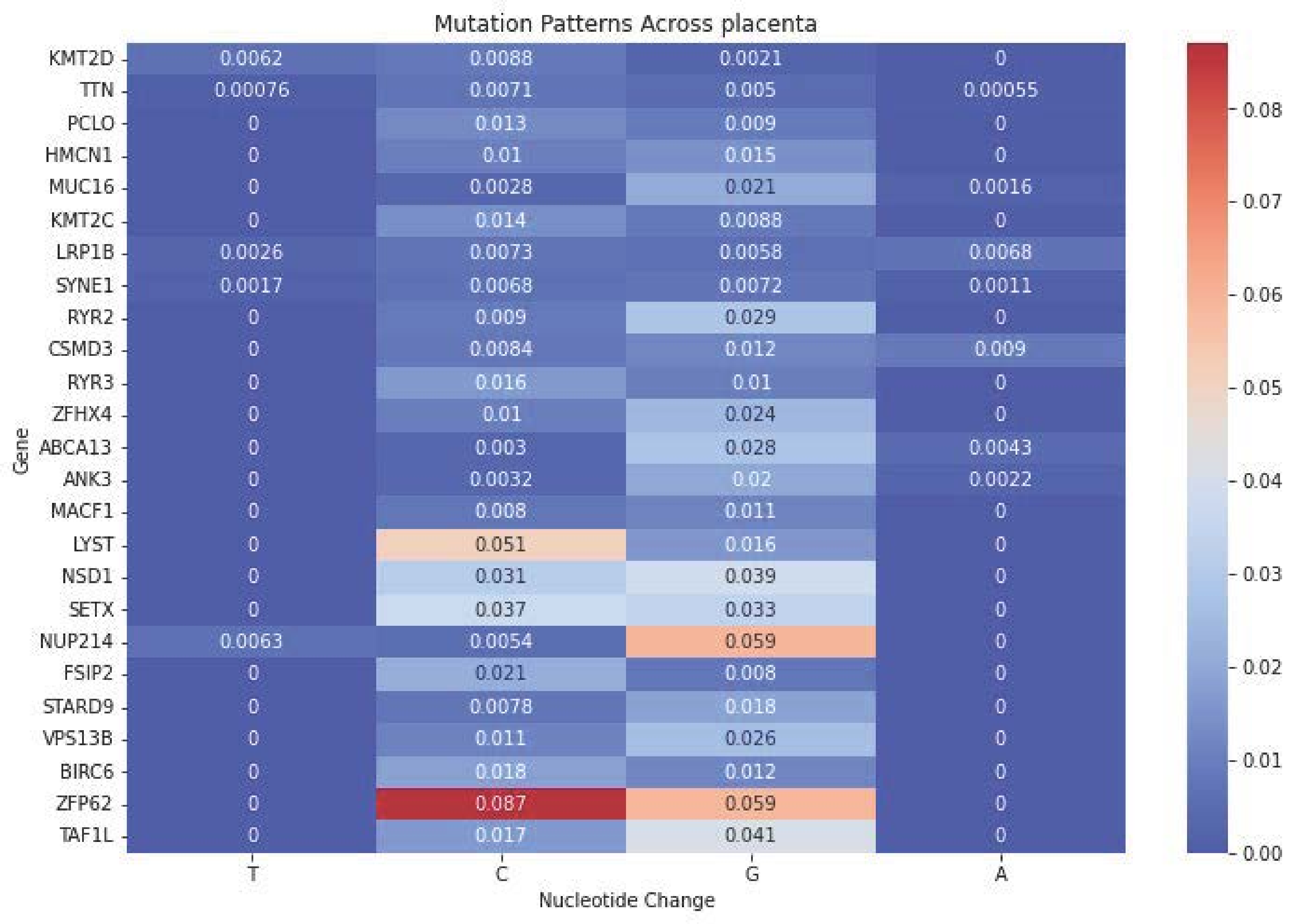

Mutation Patterns Across placenta
Gene
KMT2D
TTN
PCLO
HMCN1
MUC16
KMT2C
LRP1B
SYNE1
RYR2
CSMD3
RYR3
ZFHX4
ABCA13
ANK3
MACF1
LYST
NSD1
SETX
NUP214
FSIP2
STARD9
VPS13B
BIRC6
ZFP62
TAF1L
T
C
G
A
Nucleotide Change
0.0062 0.0088 0.0021 0
0.00076 0.0071 0.005 0.00055
0 0.013 0.009 0
0 0.01 0.015 0
0 0.0028 0.021 0.0016
0 0.014 0.0088 0
0.0026 0.0073 0.0058 0.0068
0.0017 0.0068 0.0072 0.0011
0 0.009 0.029 0
0 0.0084 0.012 0.009
0 0.016 0.01 0
0 0.01 0.024 0
0 0.003 0.028 0.0043
0 0.0032 0.02 0.0022
0 0.008 0.011 0
0 0.051 0.016 0
0 0.031 0.039 0
0 0.037 0.033 0
0.0063 0.0054 0.059 0
0 0.021 0.008 0
0 0.0078 0.018 0
0 0.011 0.026 0
0 0.018 0.012 0
0 0.087 0.059 0
0 0.017 0.041 0
0.08
0.07
0.06
0.05
0.04
0.03
0.02
0.01
0.00

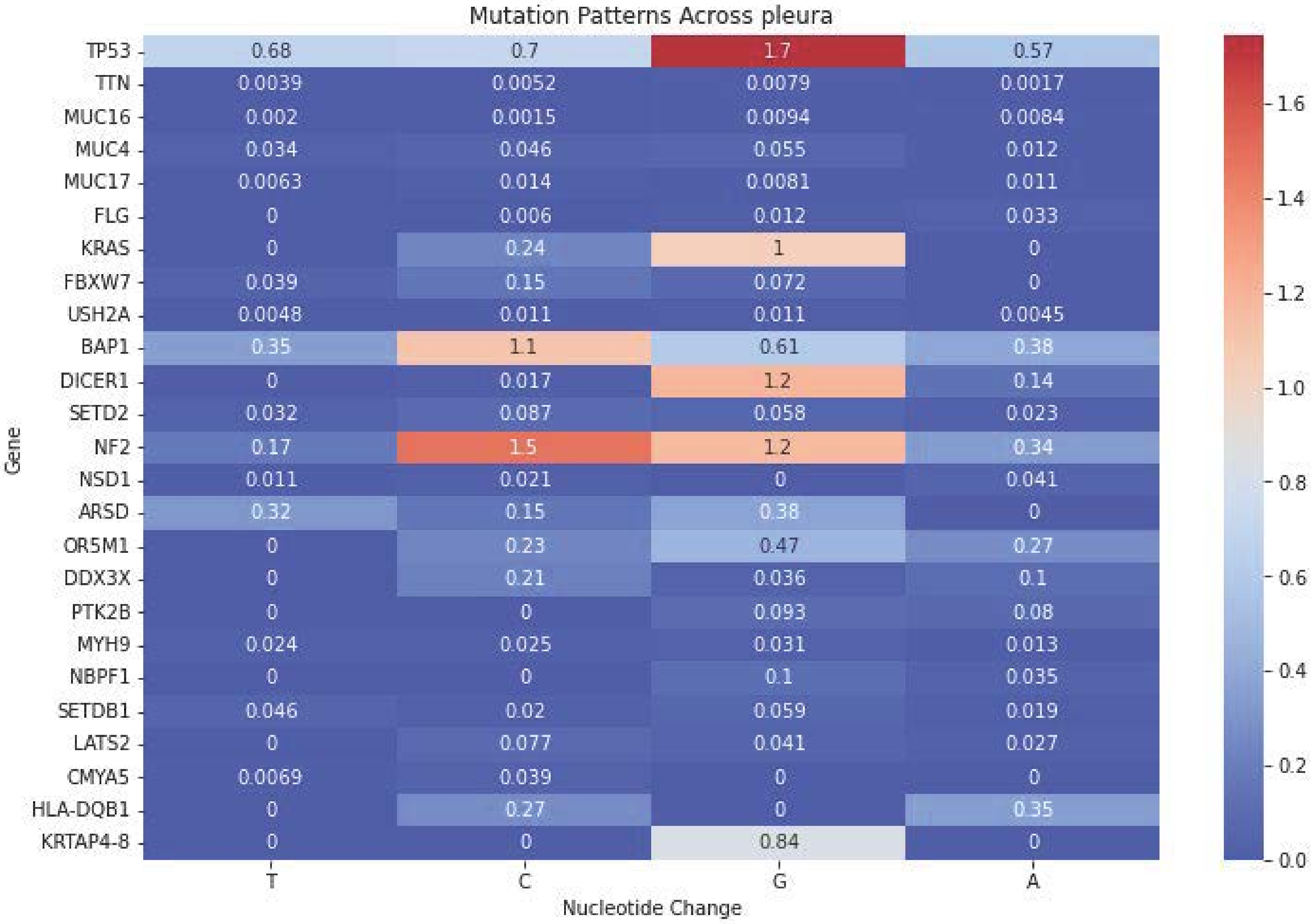

Mutation Patterns Across pleura
Gene
TP53
TTN
MUC16
MUC4
MUC17
FLG
KRAS
FBXW7
USH2A
BAP1
DICER1
SETD2
NF2
NSD1
ARSD
OR5M1
DDX3X
PTK2B
MYH9
NBPF1
SETDB1
LATS2
CMYA5
HLA-DQB1
KRTAP4-8
Nucleotide Change
T
C
G
A
0.68 0.7 1.7 0.57
0.0039 0.0052 0.0079 0.0017
0.002 0.0015 0.0094 0.0084
0.034 0.046 0.055 0.012
0.0063 0.014 0.0081 0.011
0 0.006 0.012 0.033
0 0.24 1 0
0.039 0.15 0.072 0
0.0048 0.011 0.011 0.0045
0.35 1.1 0.61 0.38
0 0.017 1.2 0.14
0.032 0.087 0.058 0.023
0.17 1.5 1.2 0.34
0.011 0.021 0 0.041
0.32 0.15 0.38 0
0 0.23 0.47 0.27
0 0.21 0.036 0.1
0 0 0.093 0.08
0.024 0.025 0.031 0.013
0 0 0.1 0.035
0.046 0.02 0.059 0.019
0 0.077 0.041 0.027
0.0069 0.039 0 0
0 0.27 0 0.35
0 0 0.84 0
1.6
1.4
1.2
1.0
0.8
0.6
0.4
0.2
0.0

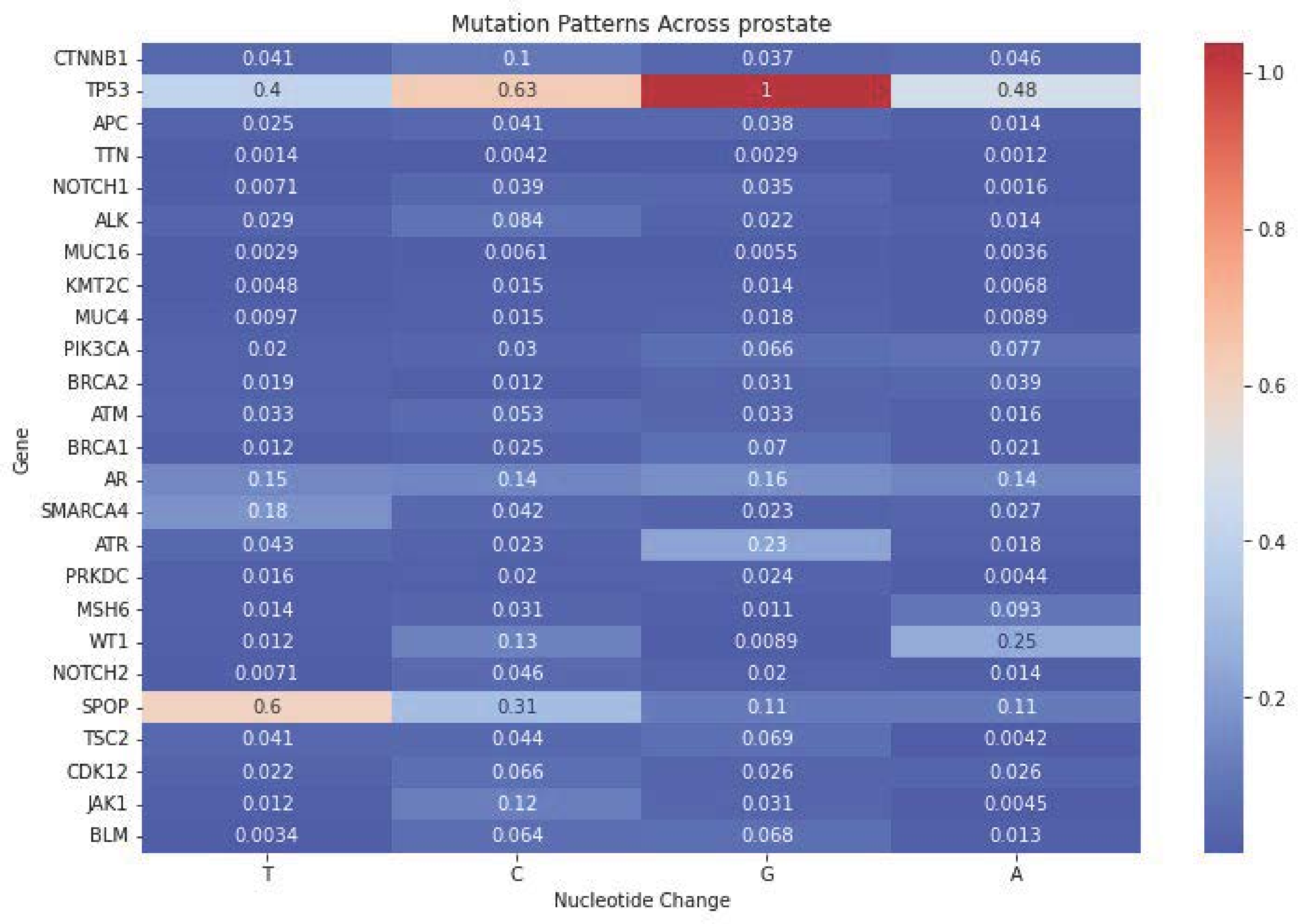
Mutation Patterns Across prostate
Gene
Nucleotide Change
T
C
G
A
CTNNB1 0.041 0.1 0.037 0.046
TP53 0.4 0.63 1 0.48
APC 0.025 0.041 0.038 0.014
TTN 0.0014 0.0042 0.0029 0.0012
NOTCH1 0.0071 0.039 0.035 0.0016
ALK 0.029 0.084 0.022 0.014
MUC16 0.0029 0.0061 0.0055 0.0036
KMT2C 0.0048 0.015 0.014 0.0068
MUC4 0.0097 0.015 0.018 0.0089
PIK3CA 0.02 0.03 0.066 0.077
BRCA2 0.019 0.012 0.031 0.039
ATM 0.033 0.053 0.033 0.016
BRCA1 0.012 0.025 0.07 0.021
AR 0.15 0.14 0.16 0.14
SMARCA4 0.18 0.042 0.023 0.027
ATR 0.043 0.023 0.23 0.018
PRKDC 0.016 0.02 0.024 0.0044
MSH6 0.014 0.031 0.011 0.093
WT1 0.012 0.13 0.0089 0.25
NOTCH2 0.0071 0.046 0.02 0.014
SPOP 0.6 0.31 0.11 0.11
TSC2 0.041 0.044 0.069 0.0042
CDK12 0.022 0.066 0.026 0.026
JAK1 0.012 0.12 0.031 0.0045
BLM 0.0034 0.064 0.068 0.013
1.0
0.8
0.6
0.4
0.2

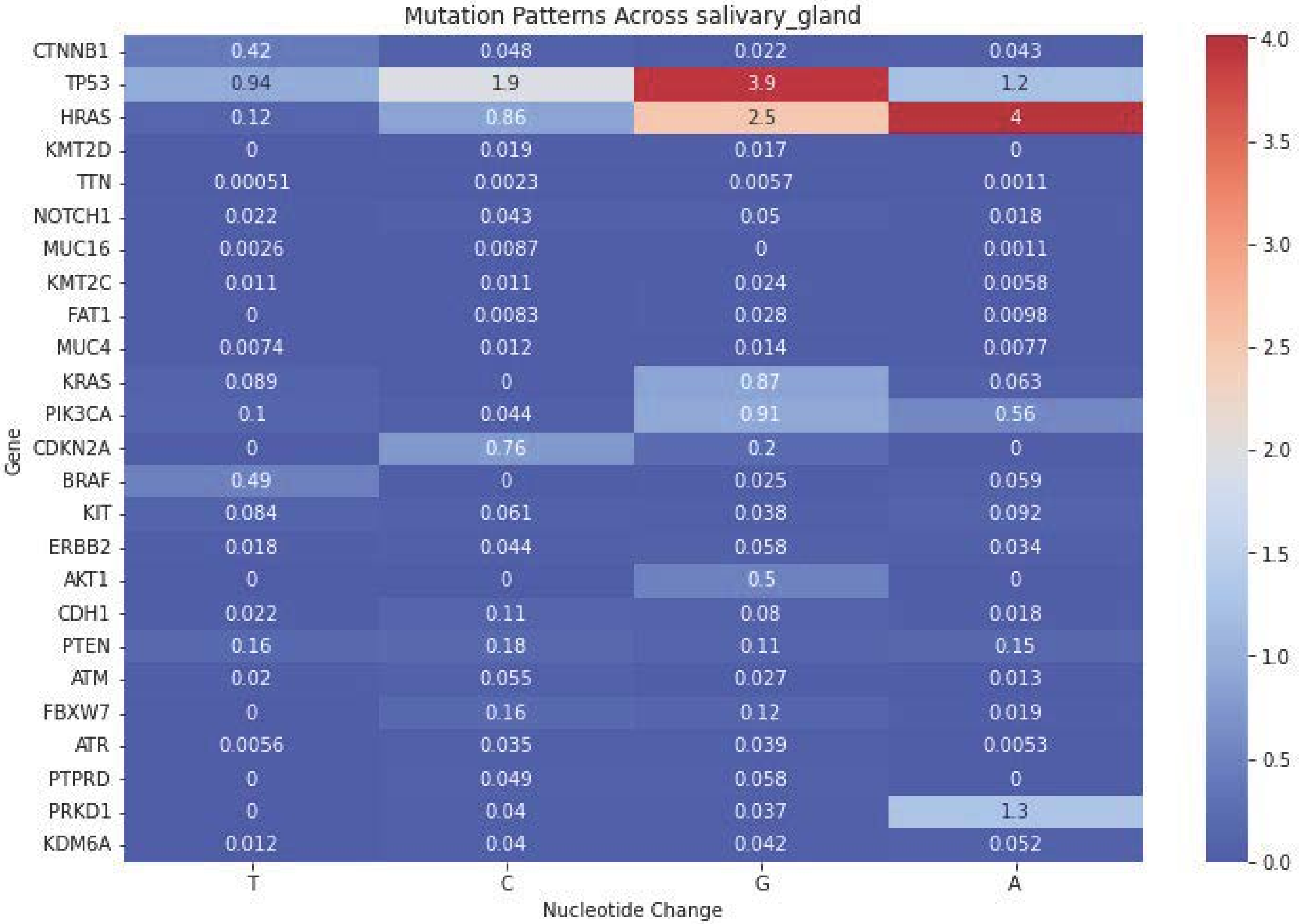

Mutation Patterns Across salivary_gland
Gene
CTNNB1
TP53
HRAS
KMT2D
TTN
NOTCH1
MUC16
KMT2C
FAT1
MUC4
KRAS
PIK3CA
CDKN2A
BRAF
KIT
ERBB2
AKT1
CDH1
PTEN
ATM
FBXW7
ATR
PTPRD
PRKD1
KDM6A
T
C
G
A
Nucleotide Change
0.42 0.048 0.022 0.043
0.94 1.9 3.9 1.2
0.12 0.86 2.5 4
0 0.019 0.017 0
0.00051 0.0023 0.0057 0.0011
0.022 0.043 0.05 0.018
0.0026 0.0087 0 0.0011
0.011 0.011 0.024 0.0058
0 0.0083 0.028 0.0098
0.0074 0.012 0.014 0.0077
0.089 0 0.87 0.063
0.1 0.044 0.91 0.56
0 0.76 0.2 0
0.49 0 0.025 0.059
0.084 0.061 0.038 0.092
0.018 0.044 0.058 0.034
0 0 0.5 0
0.022 0.11 0.08 0.018
0.16 0.18 0.11 0.15
0.02 0.055 0.027 0.013
0 0.16 0.12 0.019
0.0056 0.035 0.039 0.0053
0 0.049 0.058 0
0 0.04 0.037 1.3
0.012 0.04 0.042 0.052
4.0
3.5
3.0
2.5
2.0
1.5
1.0
0.5
0.0

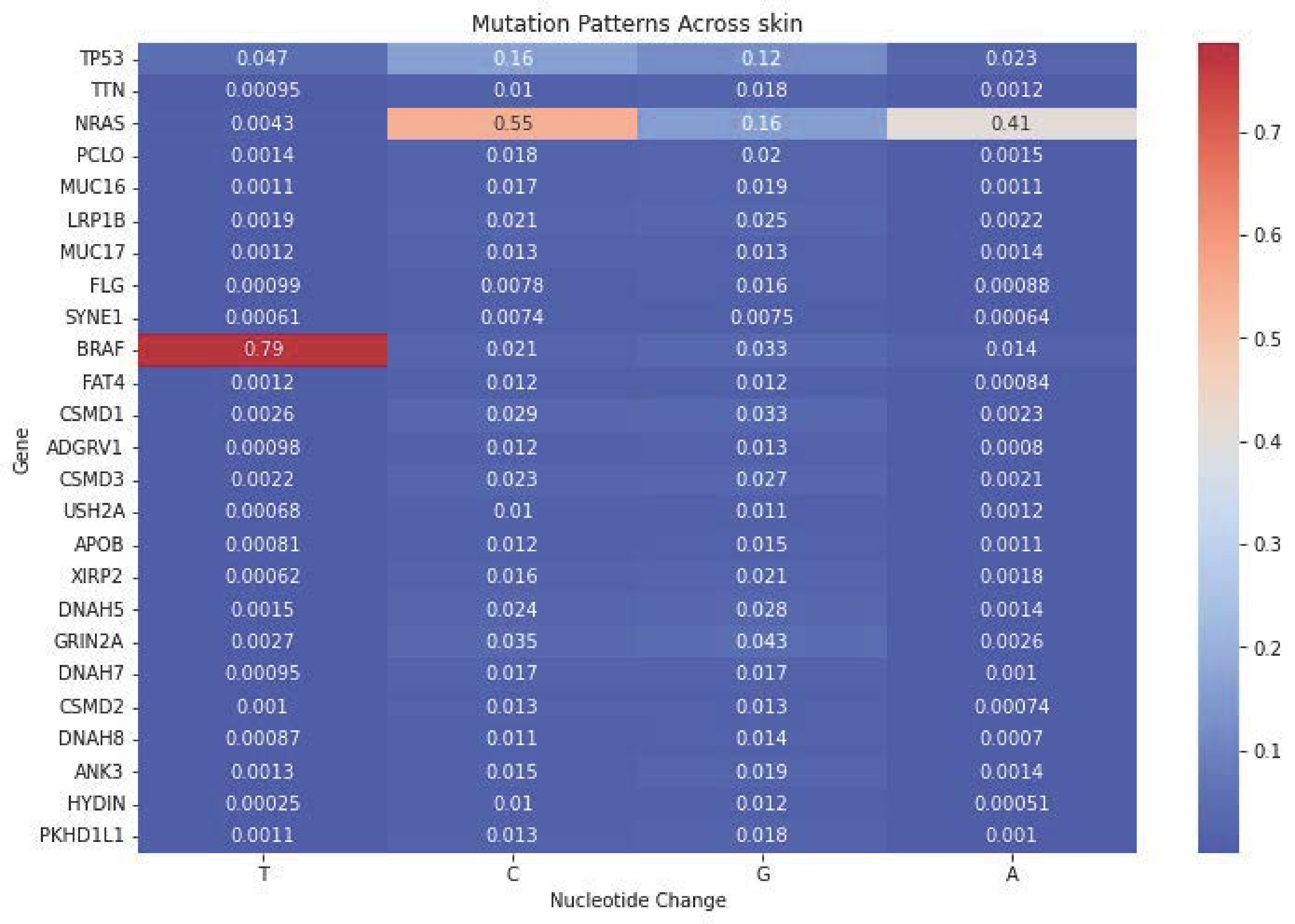
Mutation Patterns Across skin
Gene
Nucleotide Change
T
C
G
A
TP53
TTN
NRAS
PCLO
MUC16
LRP1B
MUC17
FLG
SYNE1
BRAF
FAT4
CSMD1
ADGRV1
CSMD3
USH2A
APOB
XIRP2
DNAH5
GRIN2A
DNAH7
CSMD2
DNAH8
ANK3
HYDIN
PKHD1L1
0.047 0.16 0.12 0.023
0.00095 0.01 0.018 0.0012
0.0043 0.55 0.16 0.41
0.0014 0.018 0.02 0.0015
0.0011 0.017 0.019 0.0011
0.0019 0.021 0.025 0.0022
0.0012 0.013 0.013 0.0014
0.00099 0.0078 0.016 0.00088
0.00061 0.0074 0.0075 0.00064
0.79 0.021 0.033 0.014
0.0012 0.012 0.012 0.00084
0.0026 0.029 0.033 0.0023
0.00098 0.012 0.013 0.0008
0.0022 0.023 0.027 0.0021
0.00068 0.01 0.011 0.0012
0.00081 0.012 0.015 0.0011
0.00062 0.016 0.021 0.0018
0.0015 0.024 0.028 0.0014
0.0027 0.035 0.043 0.0026
0.00095 0.017 0.017 0.001
0.001 0.013 0.013 0.00074
0.00087 0.011 0.014 0.0007
0.0013 0.015 0.019 0.0014
0.00025 0.01 0.012 0.00051
0.0011 0.013 0.018 0.001
0.7
0.6
0.5
0.4
0.3
0.2
0.1

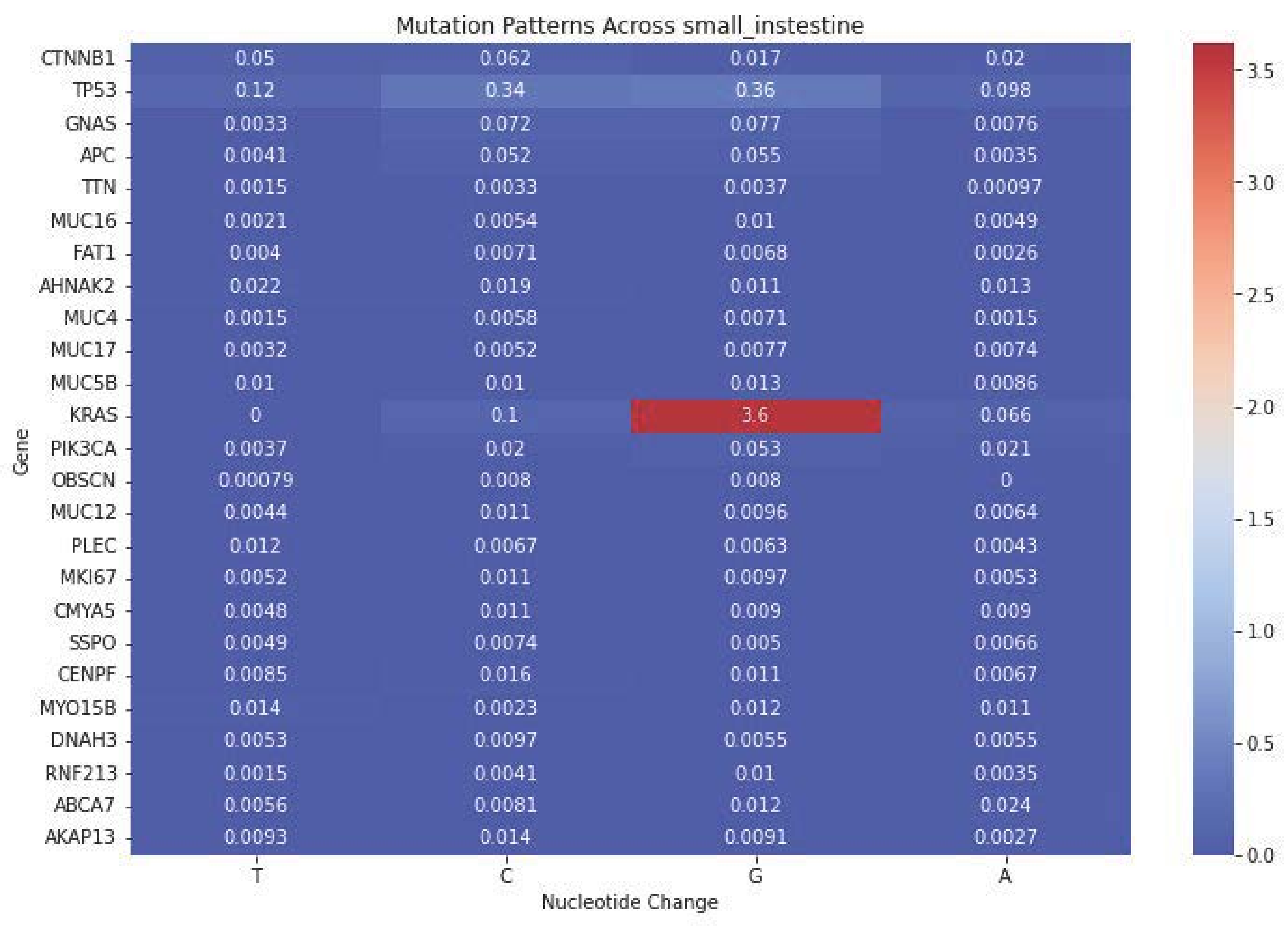

Mutation Patterns Across small_instestine
Gene
Nucleotide Change
T
C
G
A
CTNNB1 0.05 0.062 0.017 0.02
TP53 0.12 0.34 0.36 0.098
GNAS 0.0033 0.072 0.077 0.0076
APC 0.0041 0.052 0.055 0.0035
TTN 0.0015 0.0033 0.0037 0.00097
MUC16 0.0021 0.0054 0.01 0.0049
FAT1 0.004 0.0071 0.0068 0.0026
AHNAK2 0.022 0.019 0.011 0.013
MUC4 0.0015 0.0058 0.0071 0.0015
MUC17 0.0032 0.0052 0.0077 0.0074
MUC5B 0.01 0.01 0.013 0.0086
KRAS 0 0.1 3.6 0.066
PIK3CA 0.0037 0.02 0.053 0.021
OBSCN 0.00079 0.008 0.008 0
MUC12 0.0044 0.011 0.0096 0.0064
PLEC 0.012 0.0067 0.0063 0.0043
MKI67 0.0052 0.011 0.0097 0.0053
CMYA5 0.0048 0.011 0.009 0.009
SSPO 0.0049 0.0074 0.005 0.0066
CENPF 0.0085 0.016 0.011 0.0067
MYO15B 0.014 0.0023 0.012 0.011
DNAH3 0.0053 0.0097 0.0055 0.0055
RNF213 0.0015 0.0041 0.01 0.0035
ABCA7 0.0056 0.0081 0.012 0.024
AKAP13 0.0093 0.014 0.0091 0.0027
3.5
3.0
2.5
2.0
1.5
1.0
0.5
0.0

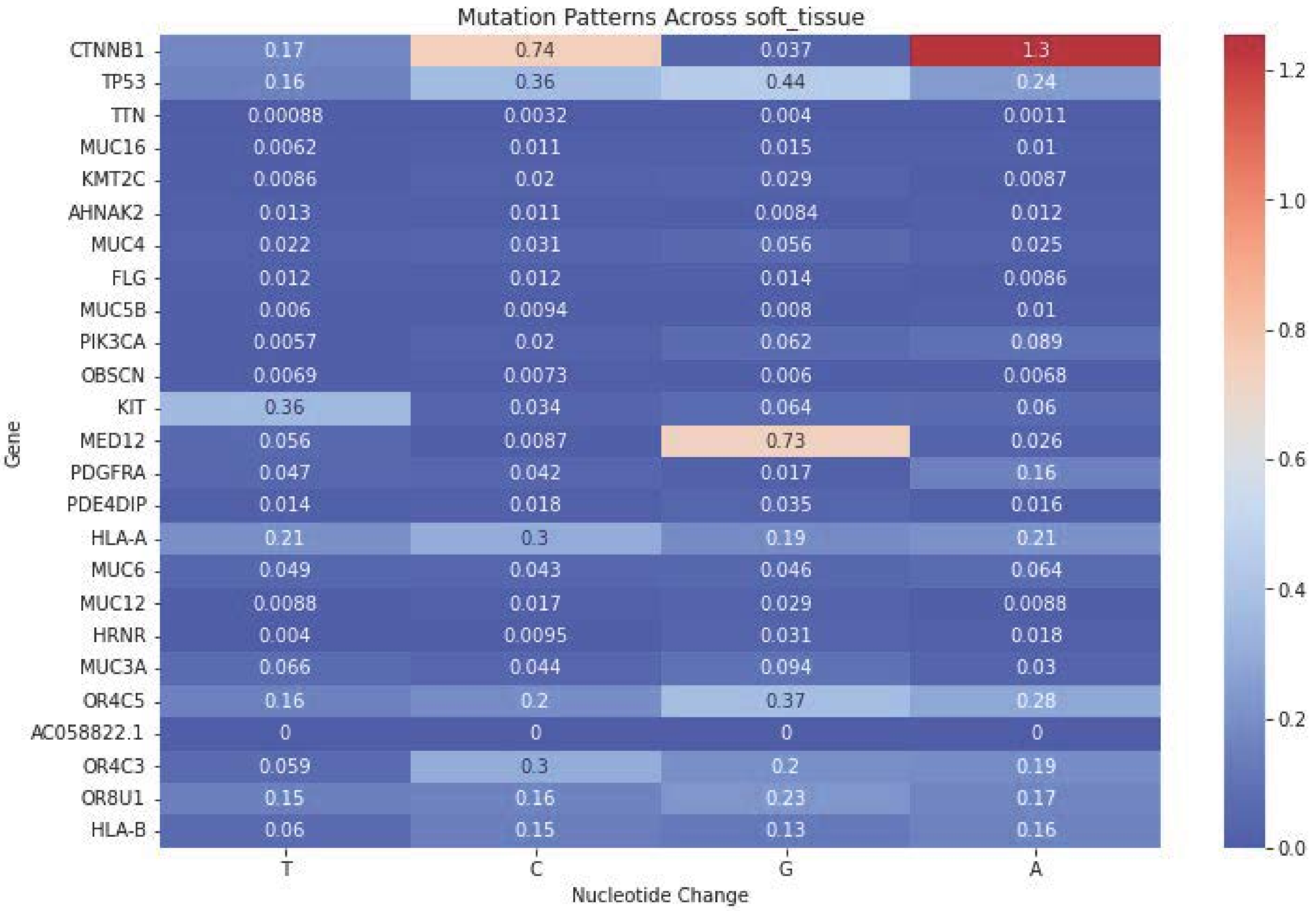
Mutation Patterns Across soft_tissue
Gene
Nucleotide Change
T
C
G
A
CTNNB1
TP53
TTN
MUC16
KMT2C
AHNAK2
MUC4
FLG
MUC5B
PIK3CA
OBSCN
KIT
MED12
PDGFRA
PDE4DIP
HLA-A
MUC6
MUC12
HRNR
MUC3A
OR4C5
AC058822.1
OR4C3
OR8U1
HLA-B
0.17 0.74 0.037 1.3
0.16 0.36 0.44 0.24
0.00088 0.0032 0.004 0.0011
0.0062 0.011 0.015 0.01
0.0086 0.02 0.029 0.0087
0.013 0.011 0.0084 0.012
0.022 0.031 0.056 0.025
0.012 0.012 0.014 0.0086
0.006 0.0094 0.008 0.01
0.0057 0.02 0.062 0.089
0.0069 0.0073 0.006 0.0068
0.36 0.034 0.064 0.06
0.056 0.0087 0.73 0.026
0.047 0.042 0.017 0.16
0.014 0.018 0.035 0.016
0.21 0.3 0.19 0.21
0.049 0.043 0.046 0.064
0.0088 0.017 0.029 0.0088
0.004 0.0095 0.031 0.018
0.066 0.044 0.094 0.03
0.16 0.2 0.37 0.28
0 0 0 0
0.059 0.3 0.2 0.19
0.15 0.16 0.23 0.17
0.06 0.15 0.13 0.16
1.2
1.0
0.8
0.6
0.4
0.2
0.0

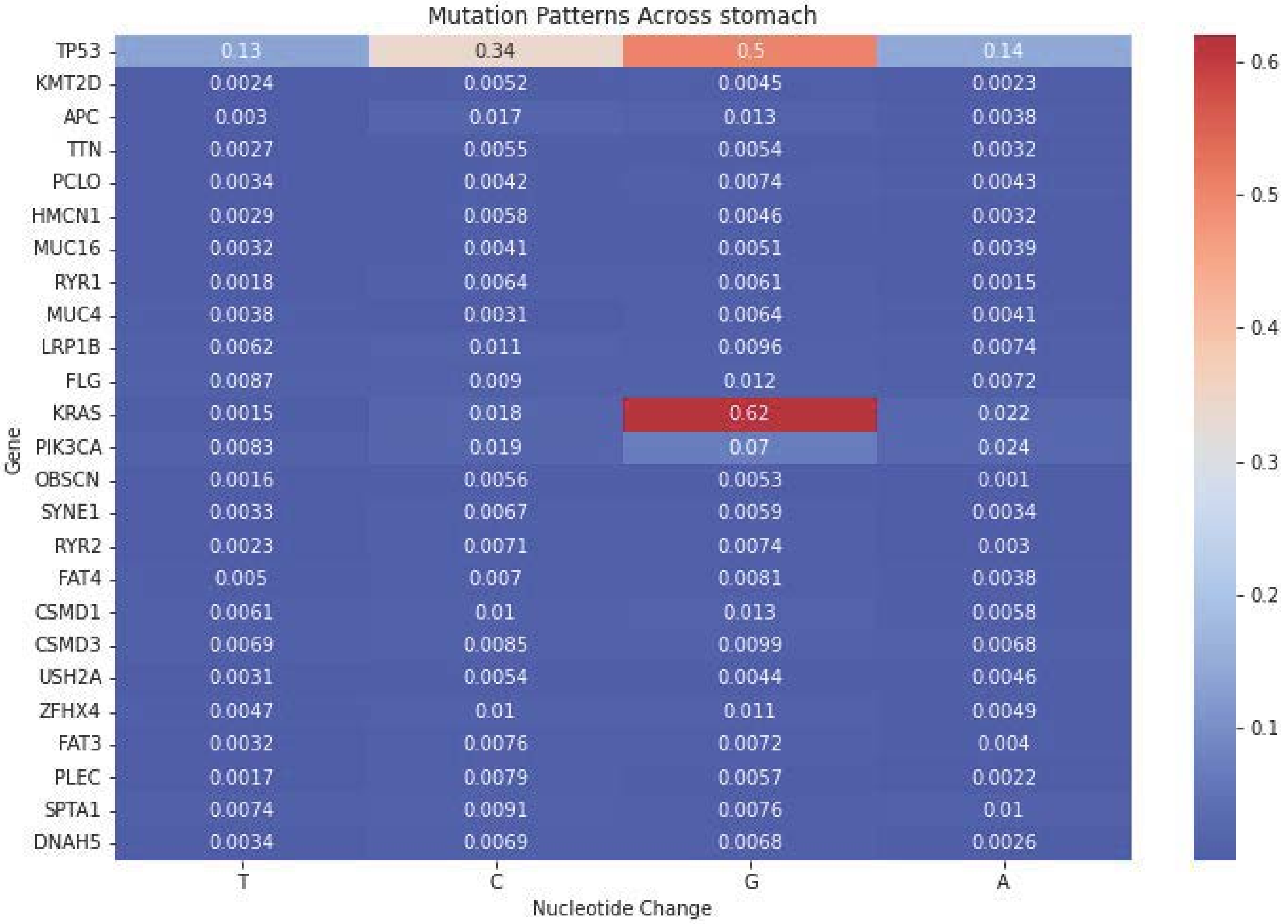

Mutation Patterns Across stomach
Gene
Nucleotide Change
T
C
G
A
TP53 0.13 0.34 0.5 0.14
KMT2D 0.0024 0.0052 0.0045 0.0023
APC 0.003 0.017 0.013 0.0038
TTN 0.0027 0.0055 0.0054 0.0032
PCLO 0.0034 0.0042 0.0074 0.0043
HMCN1 0.0029 0.0058 0.0046 0.0032
MUC16 0.0032 0.0041 0.0051 0.0039
RYR1 0.0018 0.0064 0.0061 0.0015
MUC4 0.0038 0.0031 0.0064 0.0041
LRP1B 0.0062 0.011 0.0096 0.0074
FLG 0.0087 0.009 0.012 0.0072
KRAS 0.0015 0.018 0.62 0.022
PIK3CA 0.0083 0.019 0.07 0.024
OBSCN 0.0016 0.0056 0.0053 0.001
SYNE1 0.0033 0.0067 0.0059 0.0034
RYR2 0.0023 0.0071 0.0074 0.003
FAT4 0.005 0.007 0.0081 0.0038
CSMD1 0.0061 0.01 0.013 0.0058
CSMD3 0.0069 0.0085 0.0099 0.0068
USH2A 0.0031 0.0054 0.0044 0.0046
ZFHX4 0.0047 0.01 0.011 0.0049
FAT3 0.0032 0.0076 0.0072 0.004
PLEC 0.0017 0.0079 0.0057 0.0022
SPTA1 0.0074 0.0091 0.0076 0.01
DNAH5 0.0034 0.0069 0.0068 0.0026
0.6
0.5
0.4
0.3
0.2
0.1

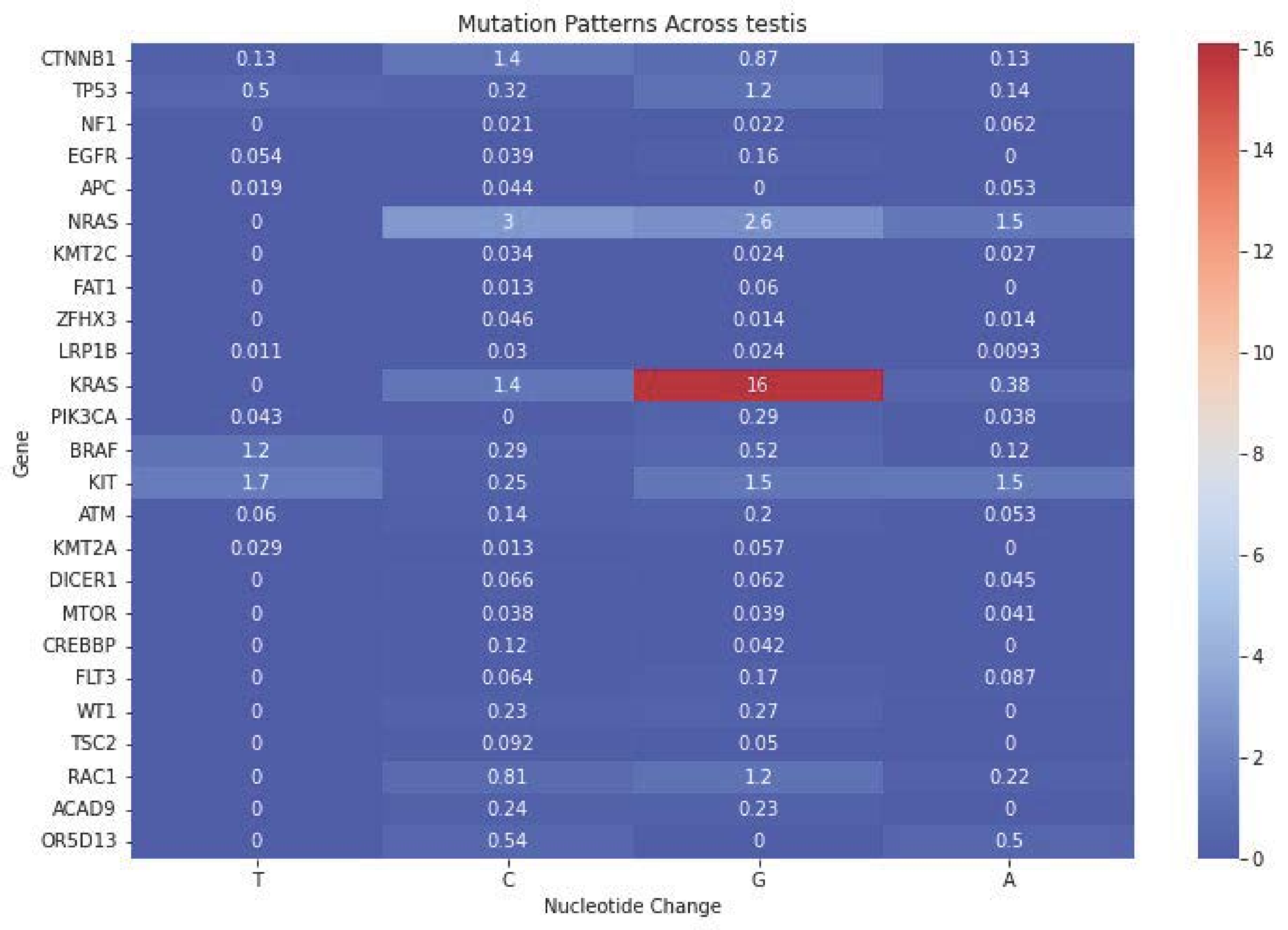
Mutation Patterns Across testis
Gene
Nucleotide Change
CTNNB1
TP53
NF1
EGFR
APC
NRAS
KMT2C
FAT1
ZFHX3
LRP1B
KRAS
PIK3CA
BRAF
KIT
ATM
KMT2A
DICER1
MTOR
CREBBP
FLT3
WT1
TSC2
RAC1
ACAD9
OR5D13
T
C
G
A
0.13 1.4 0.87 0.13
0.5 0.32 1.2 0.14
0 0.021 0.022 0.062
0.054 0.039 0.16 0
0.019 0.044 0 0.053
0 3 2.6 1.5
0 0.034 0.024 0.027
0 0.013 0.06 0
0 0.046 0.014 0.014
0.011 0.03 0.024 0.0093
0 1.4 16 0.38
0.043 0 0.29 0.038
1.2 0.29 0.52 0.12
1.7 0.25 1.5 1.5
0.06 0.14 0.2 0.053
0.029 0.013 0.057 0
0 0.066 0.062 0.045
0 0.038 0.039 0.041
0 0.12 0.042 0
0 0.064 0.17 0.087
0 0.23 0.27 0
0 0.092 0.05 0
0 0.81 1.2 0.22
0 0.24 0.23 0
0 0.54 0 0.5
16
14
12
10
8
6
4
2
0

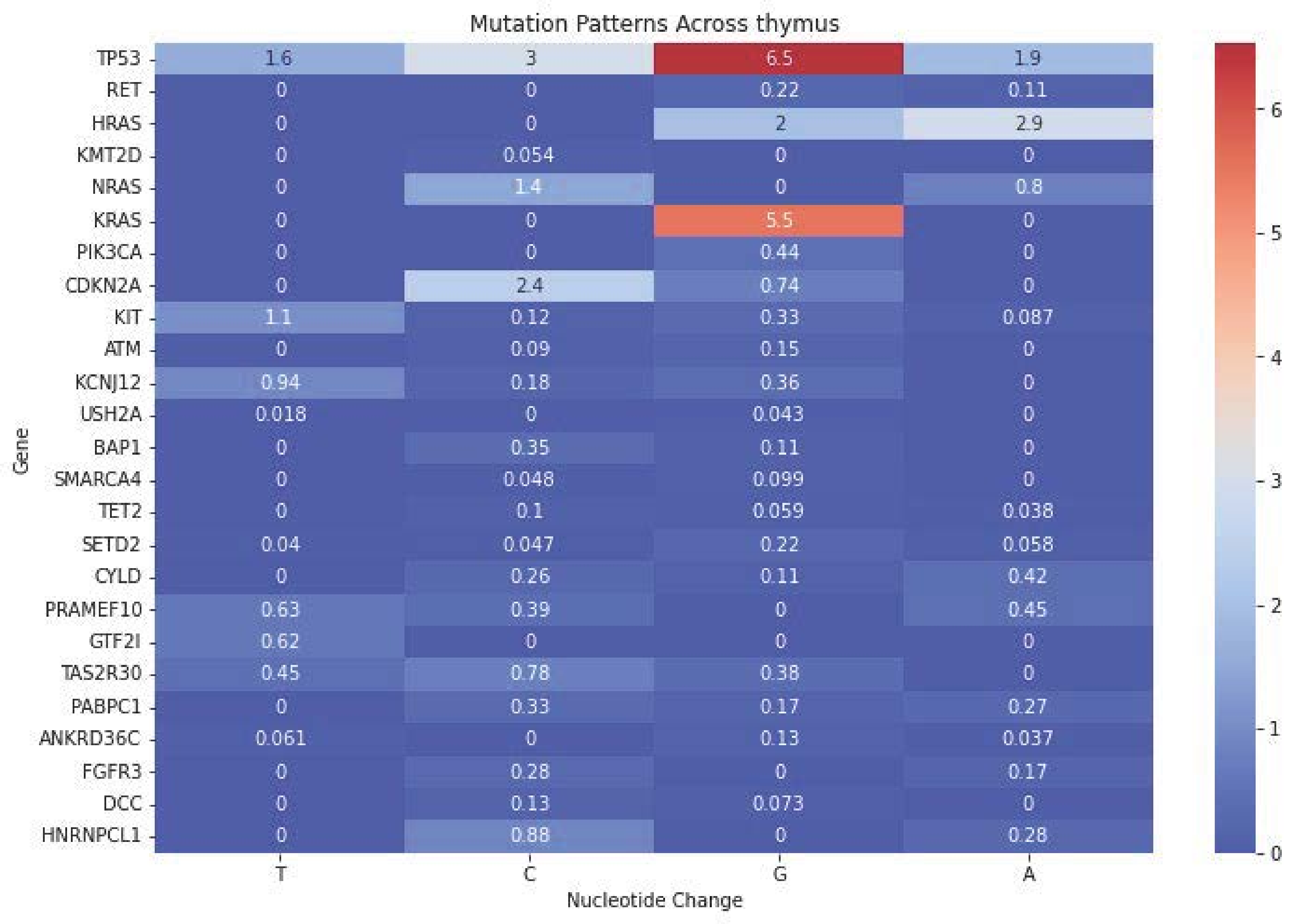
Mutation Patterns Across thymus
Gene
Nucleotide Change
T
C
G
A
TP53
RET
HRAS
KMT2D
NRAS
KRAS
PIK3CA
CDKN2A
KIT
ATM
KCNJ12
USH2A
BAP1
SMARCA4
TET2
SETD2
CYLD
PRAMEF10
GTF2I
TAS2R30
PABPC1
ANKRD36C
FGFR3
DCC
HNRNPCL1
1.6
3
6.5
1.9
0
0
0.22
0.11
0
0
2
2.9
0
0.054
0
0
0
1.4
0
0.8
0
0
5.5
0
0
0
0.44
0
0
2.4
0.74
0
1.1
0.12
0.33
0.087
0
0.09
0.15
0
0.94
0.18
0.36
0
0.018
0
0.043
0
0
0.35
0.11
0
0
0.048
0.099
0
0
0.1
0.059
0.038
0.04
0.047
0.22
0.058
0
0.26
0.11
0.42
0.63
0.39
0
0.45
0.62
0
0
0
0.45
0.78
0.38
0
0
0.33
0.17
0.27
0.061
0
0.13
0.037
0
0.28
0
0.17
0
0.13
0.073
0
0
0.88
0
0.28
0
1
2
3
4
5
6

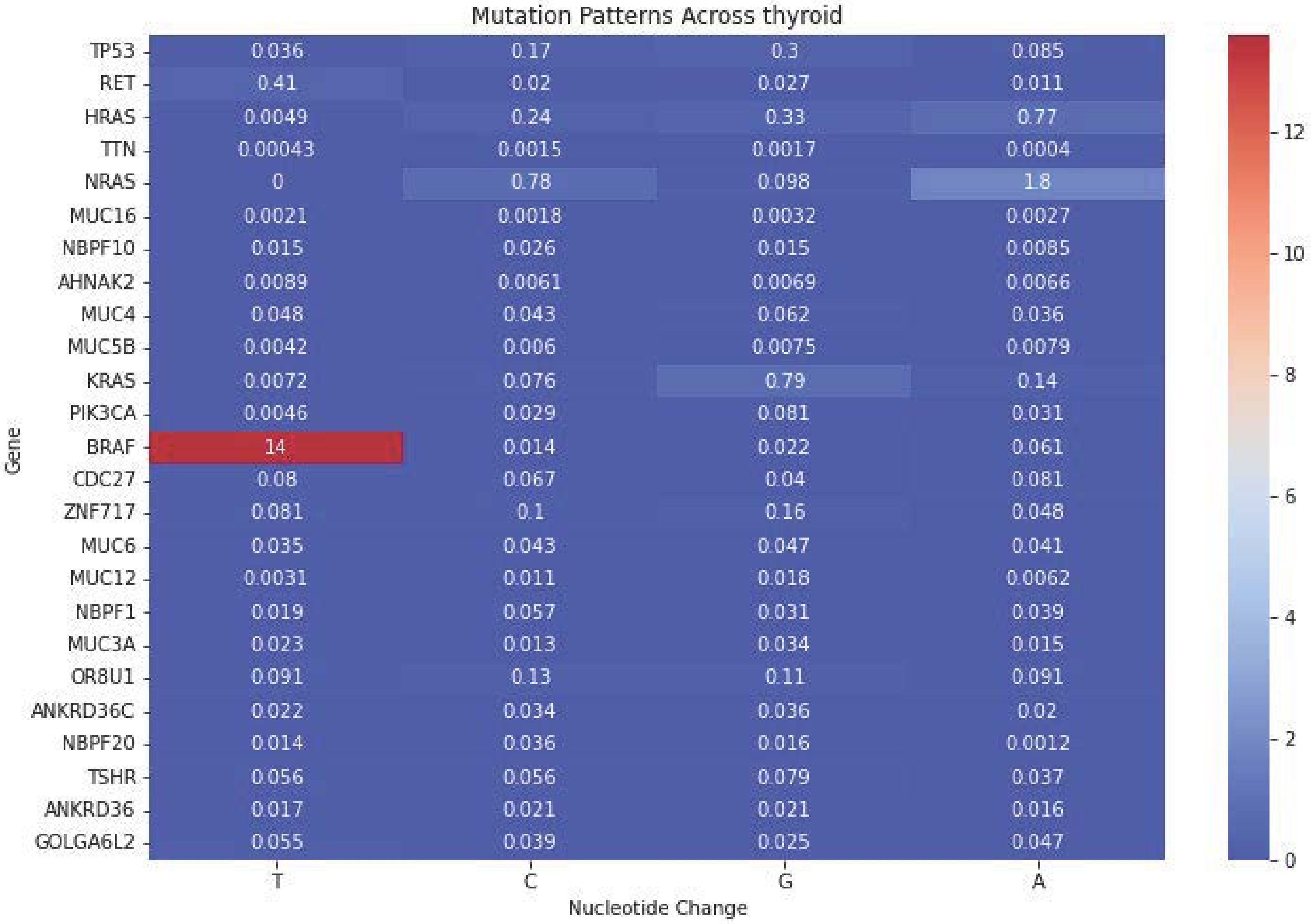

Mutation Patterns Across thyroid
Gene
Nucleotide Change
T
C
G
A
TP53
RET
HRAS
TTN
NRAS
MUC16
NBPF10
AHNAK2
MUC4
MUC5B
KRAS
PIK3CA
BRAF
CDC27
ZNF717
MUC6
MUC12
NBPF1
MUC3A
OR8U1
ANKRD36C
NBPF20
TSHR
ANKRD36
GOLGA6L2
0.036 0.17 0.3 0.085
0.41 0.02 0.027 0.011
0.0049 0.24 0.33 0.77
0.00043 0.0015 0.0017 0.0004
0 0.78 0.098 1.8
0.0021 0.0018 0.0032 0.0027
0.015 0.026 0.015 0.0085
0.0089 0.0061 0.0069 0.0066
0.048 0.043 0.062 0.036
0.0042 0.006 0.0075 0.0079
0.0072 0.076 0.79 0.14
0.0046 0.029 0.081 0.031
14 0.014 0.022 0.061
0.08 0.067 0.04 0.081
0.081 0.1 0.16 0.048
0.035 0.043 0.047 0.041
0.0031 0.011 0.018 0.0062
0.019 0.057 0.031 0.039
0.023 0.013 0.034 0.015
0.091 0.13 0.11 0.091
0.022 0.034 0.036 0.02
0.014 0.036 0.016 0.0012
0.056 0.056 0.079 0.037
0.017 0.021 0.021 0.016
0.055 0.039 0.025 0.047
12
10
8
6
4
2
0

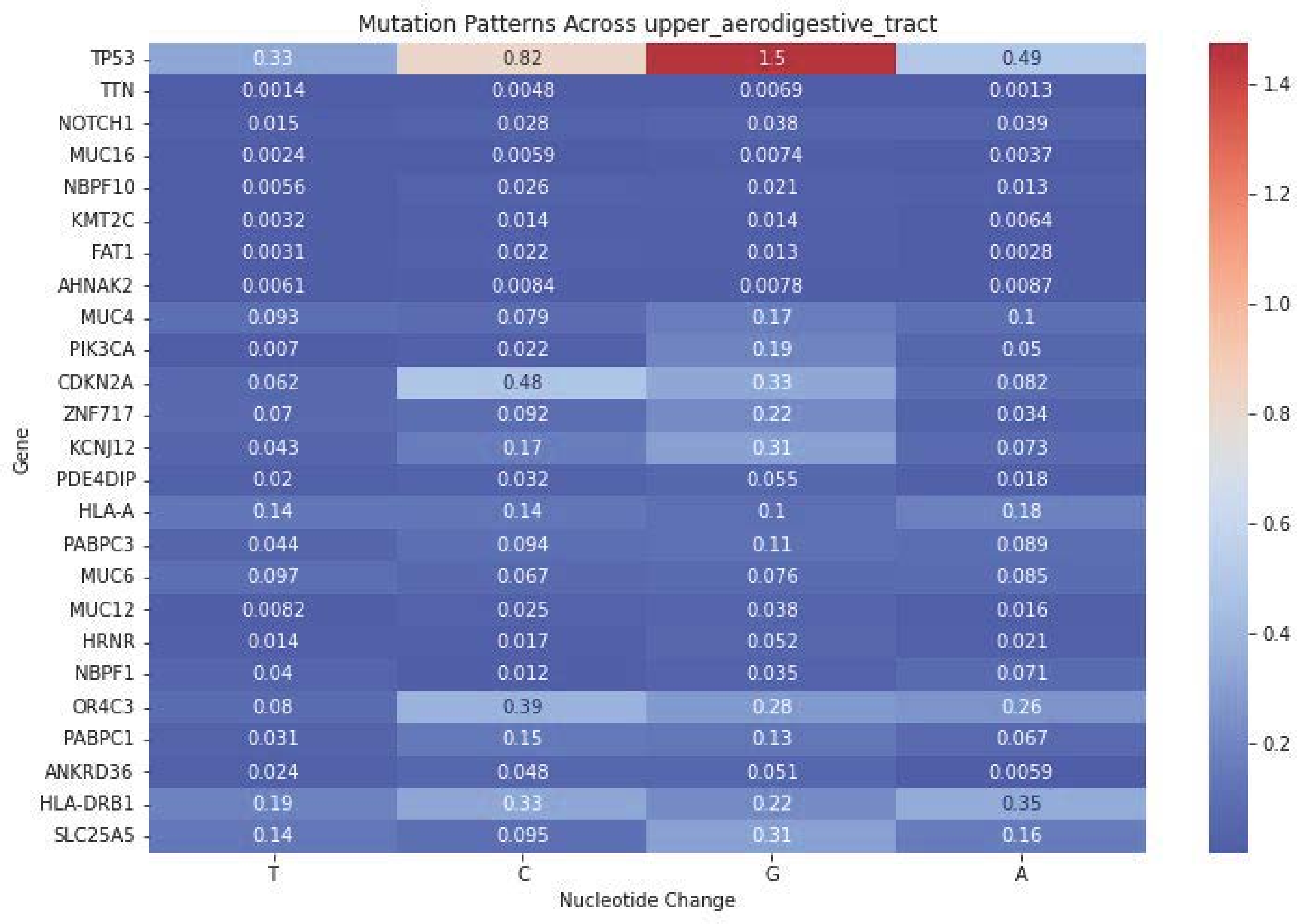
Mutation Patterns Across upper_aerodigestive_tract
Gene
Nucleotide Change
T
C
G
A
TP53 0.33 0.82 1.5 0.49
TTN 0.0014 0.0048 0.0069 0.0013
NOTCH1 0.015 0.028 0.038 0.039
MUC16 0.0024 0.0059 0.0074 0.0037
NBPF10 0.0056 0.026 0.021 0.013
KMT2C 0.0032 0.014 0.014 0.0064
FAT1 0.0031 0.022 0.013 0.0028
AHNAK2 0.0061 0.0084 0.0078 0.0087
MUC4 0.093 0.079 0.17 0.1
PIK3CA 0.007 0.022 0.19 0.05
CDKN2A 0.062 0.48 0.33 0.082
ZNF717 0.07 0.092 0.22 0.034
KCNJ12 0.043 0.17 0.31 0.073
PDE4DIP 0.02 0.032 0.055 0.018
HLA-A 0.14 0.14 0.1 0.18
PABPC3 0.044 0.094 0.11 0.089
MUC6 0.097 0.067 0.076 0.085
MUC12 0.0082 0.025 0.038 0.016
HRNR 0.014 0.017 0.052 0.021
NBPF1 0.04 0.012 0.035 0.071
OR4C3 0.08 0.39 0.28 0.26
PABPC1 0.031 0.15 0.13 0.067
ANKRD36 0.024 0.048 0.051 0.0059
HLA-DRB1 0.19 0.33 0.22 0.35
SLC25A5 0.14 0.095 0.31 0.16
1.4
1.2
1.0
0.8
0.6
0.4
0.2

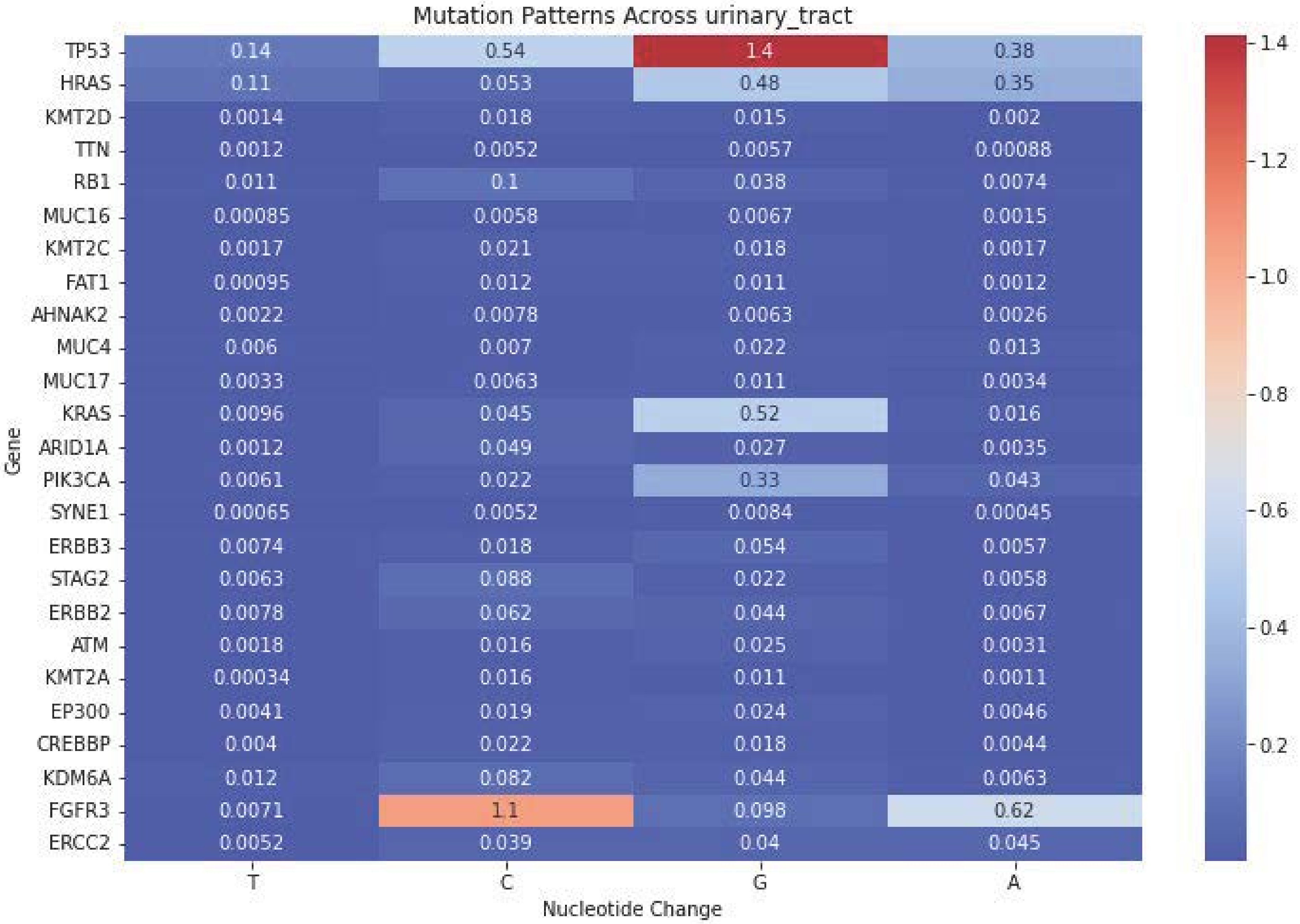
Mutation Patterns Across urinary_tract
Gene
Nucleotide Change
T
C
G
A
TP53 0.14 0.54 1.4 0.38
HRAS 0.11 0.053 0.48 0.35
KMT2D 0.0014 0.018 0.015 0.002
TTN 0.0012 0.0052 0.0057 0.00088
RB1 0.011 0.1 0.038 0.0074
MUC16 0.00085 0.0058 0.0067 0.0015
KMT2C 0.0017 0.021 0.018 0.0017
FAT1 0.00095 0.012 0.011 0.0012
AHNAK2 0.0022 0.0078 0.0063 0.0026
MUC4 0.006 0.007 0.022 0.013
MUC17 0.0033 0.0063 0.011 0.0034
KRAS 0.0096 0.045 0.52 0.016
ARID1A 0.0012 0.049 0.027 0.0035
PIK3CA 0.0061 0.022 0.33 0.043
SYNE1 0.00065 0.0052 0.0084 0.00045
ERBB3 0.0074 0.018 0.054 0.0057
STAG2 0.0063 0.088 0.022 0.0058
ERBB2 0.0078 0.062 0.044 0.0067
ATM 0.0018 0.016 0.025 0.0031
KMT2A 0.00034 0.016 0.011 0.0011
EP300 0.0041 0.019 0.024 0.0046
CREBBP 0.004 0.022 0.018 0.0044
KDM6A 0.012 0.082 0.044 0.0063
FGFR3 0.0071 1.1 0.098 0.62
ERCC2 0.0052 0.039 0.04 0.045
1.4
1.2
1.0
0.8
0.6
0.4
0.2

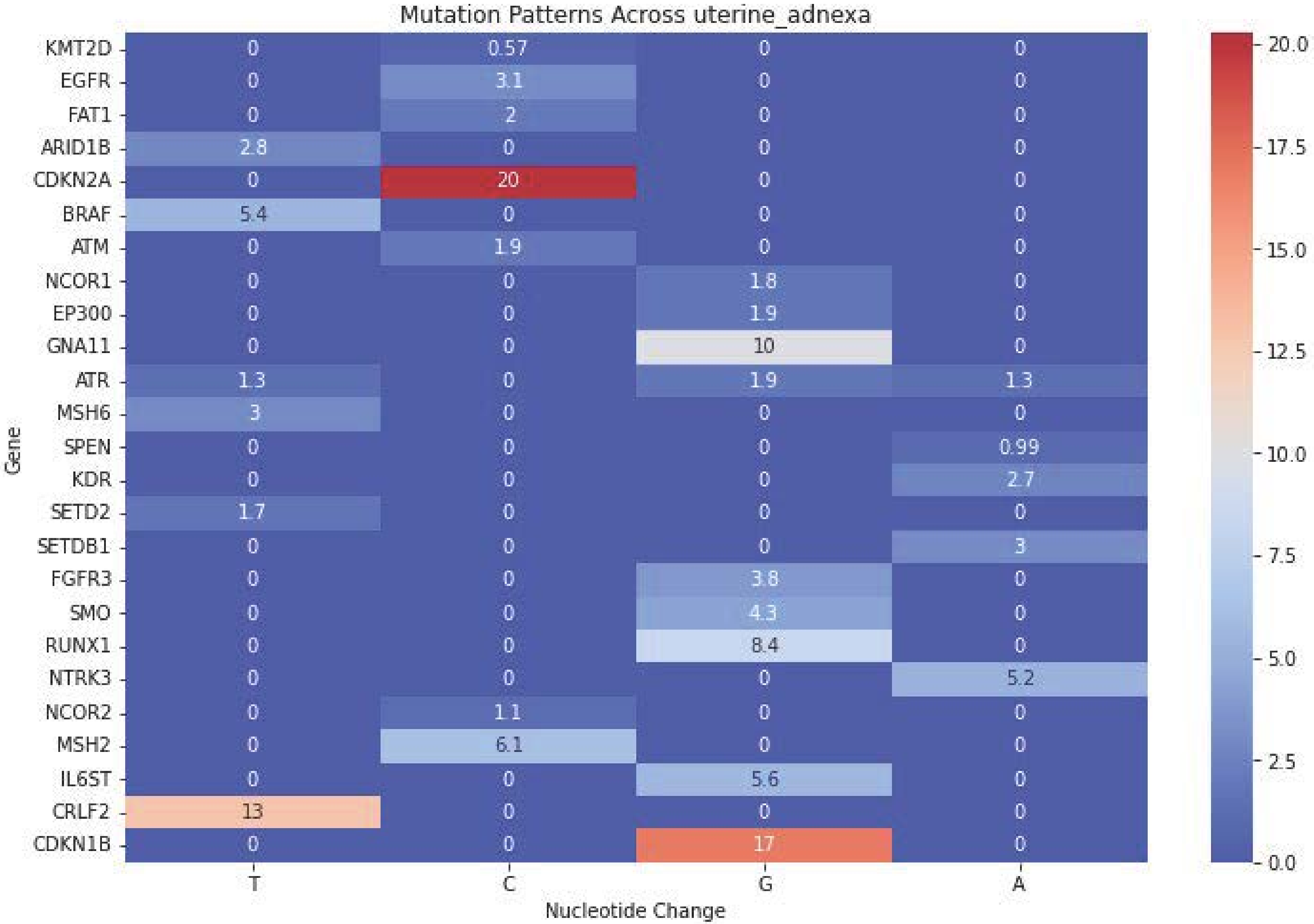
Mutation Patterns Across uterine_adnexa
Gene
KMT2D
EGFR
FAT1
ARID1B
CDKN2A
BRAF
ATM
NCOR1
EP300
GNA11
ATR
MSH6
SPEN
KDR
SETD2
SETDB1
FGFR3
SMO
RUNX1
NTRK3
NCOR2
MSH2
IL6ST
CRLF2
CDKN1B
0 0.57 0 0
0 3.1 0 0
0 2 0 0
2.8 0 0 0
0 20 0 0
5.4 0 0 0
0 1.9 0 0
0 0 1.8 0
0 0 1.9 0
0 0 10 0
1.3 0 1.9 1.3
3 0 0 0
0 0 0 0.99
0 0 0 2.7
1.7 0 0 0
0 0 0 3
0 0 3.8 0
0 0 4.3 0
0 0 8.4 0
0 0 0 5.2
0 1.1 0 0
0 6.1 0 0
0 0 5.6 0
13 0 0 0
0 0 17 0
T
C
G
A
Nucleotide Change
20.0
17.5
15.0
12.5
10.0
7.5
5.0
2.5
0.0

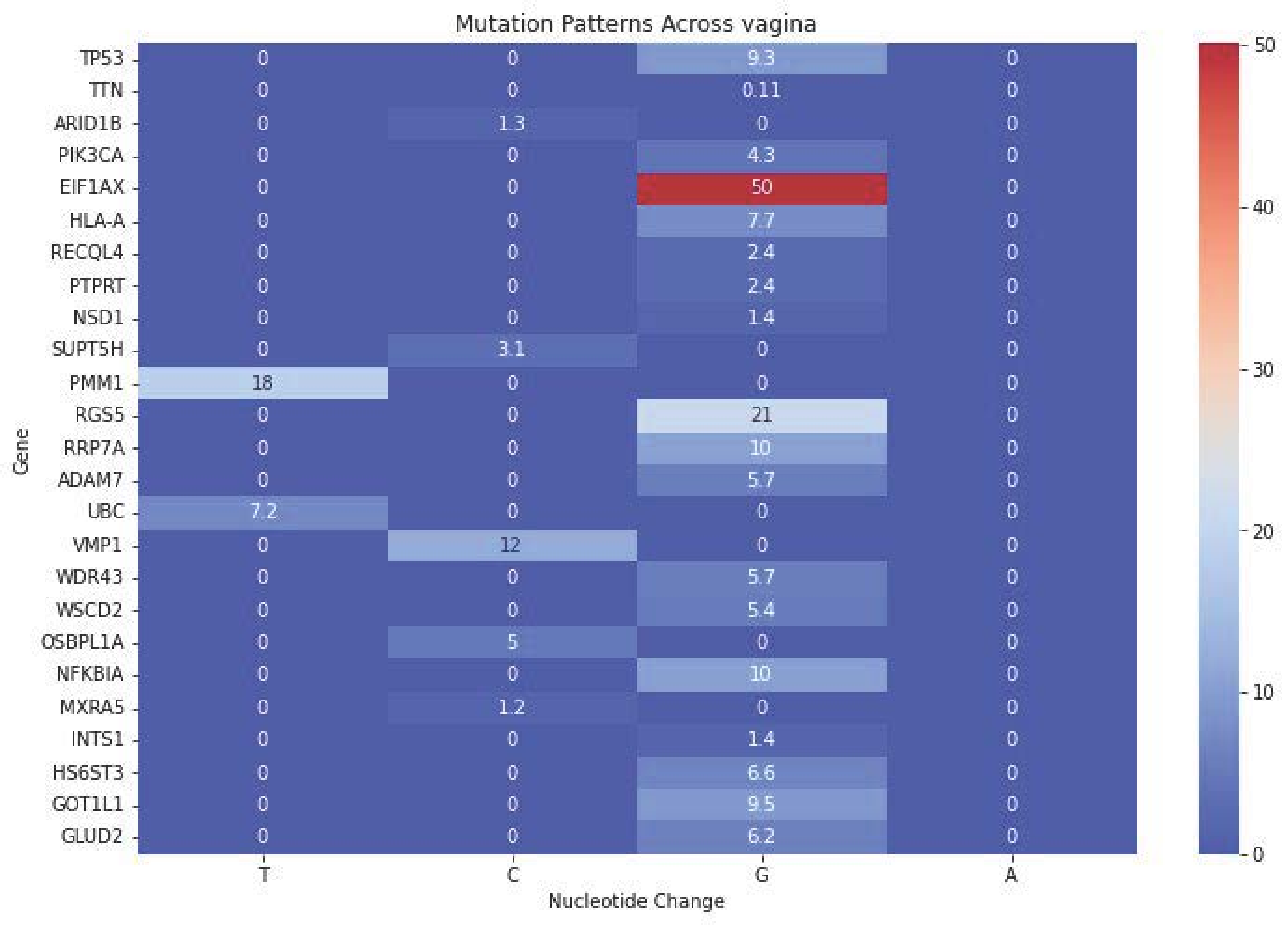
Mutation Patterns Across vagina
Gene
TP53
TTN
ARID1B
PIK3CA
EIF1AX
HLA-A
RECQL4
PTPRT
NSD1
SUPT5H
PMM1
RGS5
RRP7A
ADAM7
UBC
VMP1
WDR43
WSCD2
OSBPL1A
NFKBIA
MXRA5
INTS1
HS6ST3
GOT1L1
GLUD2
T
C
G
A
Nucleotide Change
0 0 9.3 0
0 0 0.11 0
0 1.3 0 0
0 0 4.3 0
0 0 50 0
0 0 7.7 0
0 0 2.4 0
0 0 2.4 0
0 0 1.4 0
0 3.1 0 0
18 0 0 0
0 0 21 0
0 0 10 0
0 0 5.7 0
7.2 0 0 0
0 12 0 0
0 0 5.7 0
0 0 5.4 0
0 5 0 0
0 0 10 0
0 1.2 0 0
0 0 1.4 0
0 0 6.6 0
0 0 9.5 0
0 0 6.2 0
50
40
30
20
10
0

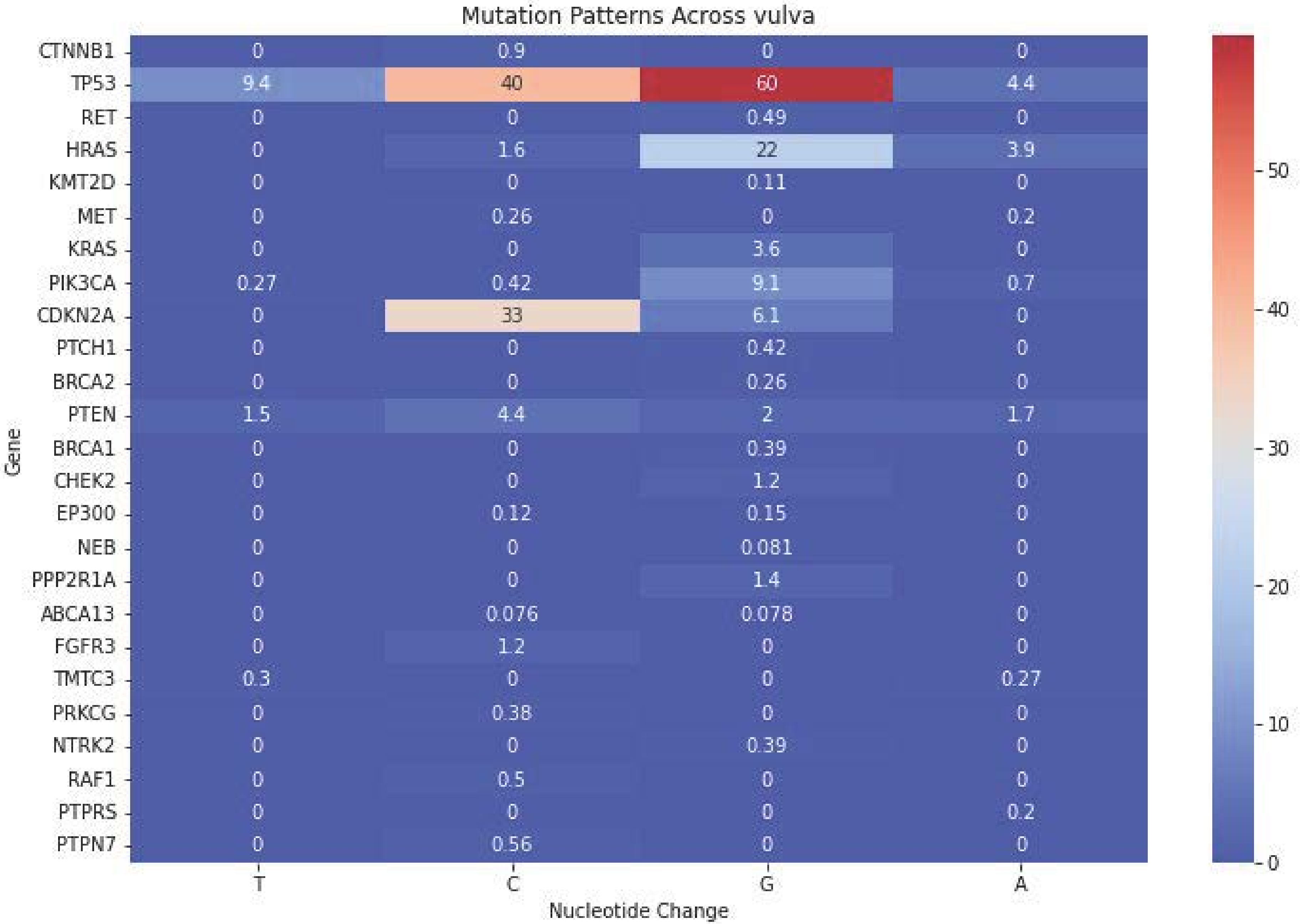

Mutation Patterns Across vulva
Gene
Nucleotide Change
T
C
G
A
CTNNB1
TP53
RET
HRAS
KMT2D
MET
KRAS
PIK3CA
CDKN2A
PTCH1
BRCA2
PTEN
BRCA1
CHEK2
EP300
NEB
PPP2R1A
ABCA13
FGFR3
TMTC3
PRKCG
NTRK2
RAF1
PTPRS
PTPN7
0
10
20
30
40
50
