# Supplementary material for: Nucleotide Substitution Biases in Related Cancer Driver Genes
Source: Int J Mol Sci. 2025 Dec 10;26(24):11903. doi: 10.3390/ijms262411903 (PMC12732676; doi:10.3390/ijms262411903)

Supplementary Figure S2. Cancer specific statistically significant mutations producing nucleotide substitution skews. **A**. Nucleotide type where high frequency mutations from genes in Fig.3B occur. B. Nucleotide type to which high frequency mutations from Fig. 3B substitute. C. Nucleotide substitution patterns of genes in Fig.3B and Fig.4.

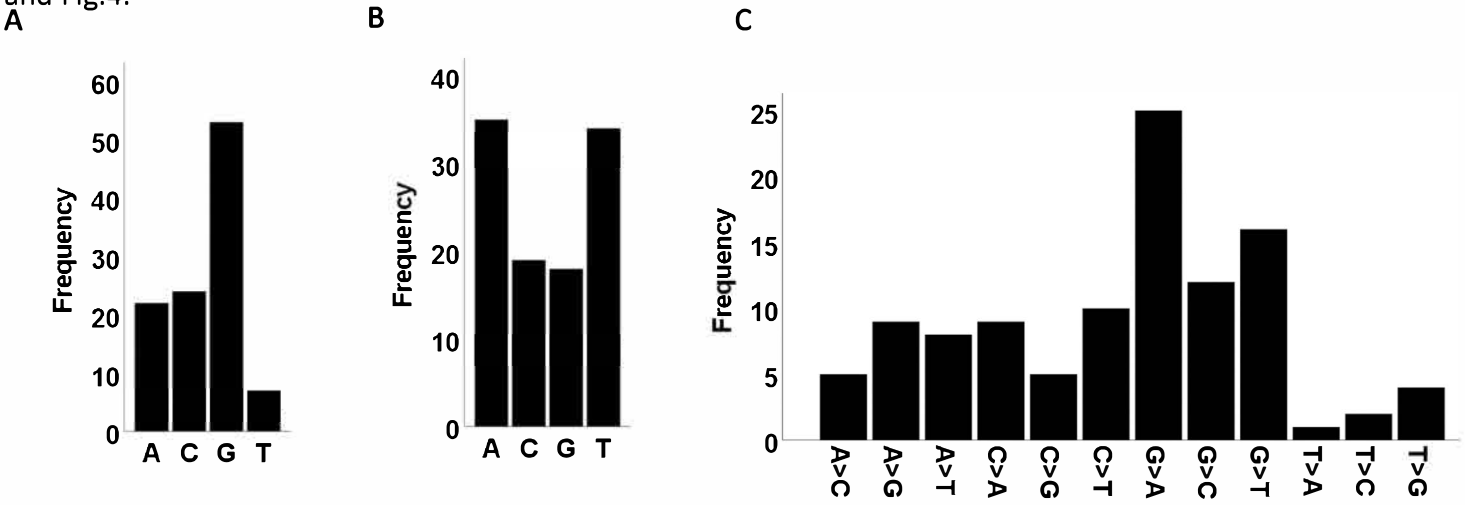

Supplement: Supplementary file 1 [file ijms-26-11903-s001.zip › Supplementary Figure S2.pdf]
